# Multifunctional Conductive and Elastic Matrices-Engineered Si Nanocomposite Anodes for Liquid and Solid-State Lithium Batteries

**DOI:** 10.1007/s40820-026-02258-w

**Published:** 2026-06-22

**Authors:** Young-Han Lee, Je-Hyeon Han, Deok-Gyu Kim, Jung-Woon Yoo, Yoon-Cheol Ha, Jae-Hun Kim, Cheol-Min Park

**Affiliations:** 1 Department of Advanced Materials Science and Engineering, Kumoh National Institute of Technology, Gumi, Gyeongbuk 39177 Republic of Korea; 2 Department of Energy Engineering Convergence, Kumoh National Institute of Technology, Gumi, Gyeongbuk 39177 Republic of Korea; 3 Battery Research Division, Korea Electrotechnology Research Institute (KERI), Changwon, Gyeongnam 51543 Republic of Korea; 4 Electric Energy Materials Engineering, KERI School, University of Science and Technology (UST), Daejeon, 34113 Republic of Korea; 5 School of Materials Science and Engineering, Kookmin University, Seoul, 02707 Republic of Korea

**Keywords:** Lithium-ion battery, All-solid-state lithium battery, Si-based anode, Sulfide solid electrolyte, Multifunctional matrices

## Abstract

Silicon anodes have intrinsically low electronic conductivity and severe volume changes, leading to nonuniform reaction kinetics and progressive structural degradation in both lithium-ion batteries (LIBs) and all-solid-state lithium batteries (ASSLBs). To overcome these limitations, we develop a silicon nanocomposite anode via a scalable and facile synthesis route. The nanocomposite (Si/a-Sn/CoSi_2_/G/C) consists of ultrafine Si nanocrystallites integrated with a well-deformable, electronically conductive amorphous Sn; a mechanically robust and elastic CoSi_2_ framework; a highly Li-reversible, electronically conductive, stress-mitigating graphite scaffold; and a highly elastic, electronically conductive PVC-pyrolyzed amorphous carbon shell. This hierarchical and synergistic architecture integrates uniform nanocrystalline Si dispersion, continuous electronic conduction, and mechanically rigid and elastically buffering matrices that accommodate volume expansion, thereby establishing a robust Si nanocomposite anode platform compatible with both LIBs and ASSLBs. The anode has a high reversible capacity, stable long-term cycling performance, high Coulombic efficiency, and improved rate capability. In LIB systems, a Si/a-Sn/CoSi_2_/G/C|NCM811 full-cell achieves an energy density of 434.4 Wh kg^–1^ with durable cycling stability. In sulfide-based ASSLB systems employing Li_6_PS_5_Cl, the full-cell has an energy density exceeding 300 Wh kg^–1^, with structural and electrochemical stability. Thus, Si/a-Sn/CoSi_2_/G/C is a practical and scalable Si-based anode platform for next-generation LIBs and ASSLBs.

**Supplementary Information:**

The online version contains supplementary material available at 10.1007/s40820-026-02258-w.

## Highlights

- A Si nanocomposite (Si/a-Sn/CoSi_2_/G/C) anode incorporating multifunctional conductive–elastic matrices was fabricated via a simple and scalable route.
- The multimatrix design sustains electronic percolation, facilitates Li^+^ transport, and provides multilevel stress buffering with elastic recovery to mitigate Si chemo-mechanical degradation.
- Si/a-Sn/CoSi_2_/G/C-based full-cells achieve high energy densities in both liquid and solid-state systems (434.4 Wh kg^–1^ in LIBs and >300 Wh kg^–1^ in all-solid-state lithium batteries) with durable cycling stability.

**Supplementary Information:**

The online version contains supplementary material available at 10.1007/s40820-026-02258-w.

## Graphical abstract

**Figure:**
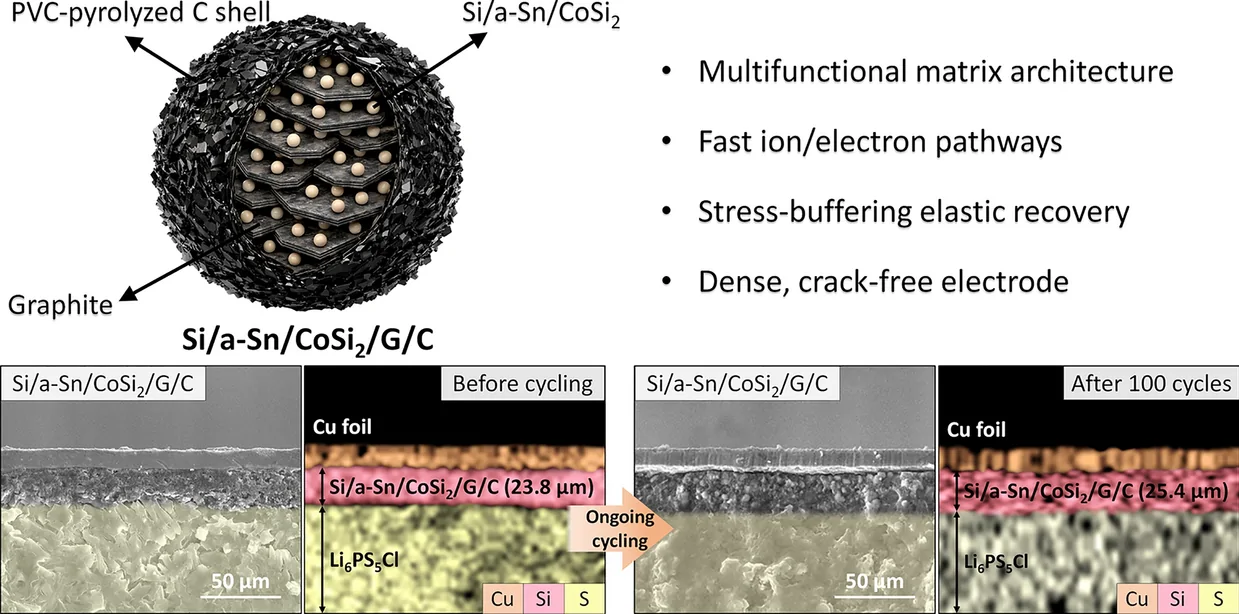

## Introduction

The rapid advancement of energy storage technologies for portable electronics, electric vehicles (EVs), and grid-scale renewable energy systems has intensified the demand for lithium-ion batteries (LIBs) and all-solid-state lithium batteries (ASSLBs) with higher energy density, longer cycle life, and improved safety. LIBs currently dominate commercial markets owing to their well-established balance between energy density and durability [1]. Meanwhile, ASSLBs have emerged as promising next-generation systems because nonflammable solid electrolytes (SEs) can improve their safety and potentially enable higher energy densities by expanding the usable electrode and facilitating compact cell designs [2, 3]. Achieving these targets in both LIBs and ASSLBs requires advanced anode materials beyond graphite, whose limited theoretical capacity (372 mAh g^–1^) constrains further increases in energy density and thus cannot meet the escalating demands for an extended driving range, prolonged device operation, and large-scale energy storage.

Silicon has emerged as a promising high-capacity anode material for both LIBs and ASSLBs owing to its high theoretical capacity (3579 mAh g^–1^, based on Li_3.75_Si), low lithiation potential (< 0.4 V *vs.* Li^+^/Li), and natural abundance [4]. However, its practical implementation is fundamentally limited by severe chemo-mechanical and interfacial instabilities. Extreme volume changes exceeding 300% during repeated lithiation and delithiation induce particle pulverization, electrical isolation, and the continuous formation of unstable interphases at both liquid and solid electrolyte interfaces [5, 6]. Moreover, the intrinsically low electronic conductivity of Si impedes the reaction kinetics, promotes heterogeneous lithiation, and intensifies localized stress accumulation. These challenges become more critical in ASSLBs, where rigid solid–solid interfaces are prone to stress-driven delamination, rapid impedance growth, and accelerated capacity fading. Diverse structural design strategies for Si anodes have been extensively explored in recent studies, including Si nanostructuring [7, 8], interface engineering [9], and low-dimensional or carbon-based architectures [10–16]. In particular, Si/C composite architectures have been widely investigated to enhance structural stability and charge transport. These approaches have significantly improved electrochemical performance by alleviating volume expansion and enhancing conductivity. However, many of these strategies rely on high fractions of carbonaceous components or complex nanostructures to alleviate volume changes and improve conductivity, which can reduce the effective active material fraction and hinder practical scalability. Furthermore, the simultaneous optimization of mechanical robustness, electrical conductivity, and structural stability remains challenging in conventional systems. In this context, the development of structurally integrated yet scalable architectures that can concurrently address mechanical stability, charge transport, and practical manufacturability remains a critical challenge. Accordingly, composite engineering employing conductive, mechanically robust, and elastic matrices that enhance charge transport and buffer volume fluctuations has emerged as a practical route to stabilize Si. Among the candidate matrices, transition metal silicides (TMSs), such as FeSi_2_, CoSi_2_, and NiSi_2_, have been widely investigated because of their high intrinsic electronic conductivity, mechanical robustness, and chemical compatibility with Si [17–27]. Nevertheless, many TMS-based Si composites still exhibit limited durability and nonuniform distributions, thereby triggering localized mechanical failure. Accordingly, achieving a homogeneous dispersion of Si within the TMS matrix, together with the integration of complementary conductive and stress-buffering components, is essential for stable long-term cycling. In most previous studies, conductive and mechanically adaptive matrices have been incorporated into Si anodes as a practical strategy to enhance charge transport and mechanical stability [10, 28, 29]. However, these approaches largely rely on a single matrix component, limiting the simultaneous optimization of electronic conductivity, mechanical robustness, and strain accommodation. Therefore, a functionally differentiated multimatrix design is required, in which distinct matrices are independently optimized and synergistically integrated, providing a more rational framework for balancing electrochemical and mechanical stability in Si anodes.

In this study, a Si-based nanocomposite anode (Si/a-Sn/CoSi_2_/G/C) was developed by integrating multifunctional matrices, including deformable and conductive amorphous Sn; a mechanically robust and elastic CoSi_2_ framework; a highly Li-reversible, stress-mitigating graphite scaffold; and a highly elastic, electronically conductive poly(vinyl chloride) (PVC)-pyrolyzed amorphous carbon shell. This multimatrix architecture enabled the uniform dispersion of ultrafine Si nanocrystallites, effectively accommodating volume fluctuations while maintaining continuous charge transport and interparticle/interfacial contact during cycling. As a result, the anode exhibited outstanding cycling stability, an excellent rate capability, and a high energy density in both liquid electrolyte LIBs and sulfide-based ASSLBs. Full-cells (Si/a-Sn/CoSi_2_/G/C|NCM811) achieved high energy densities of 434.4 Wh kg^–1^ in LIBs and > 300 Wh kg^–1^ in sulfide-based ASSLBs, demonstrating the strong potential of the Si/a-Sn/CoSi_2_/G/C anode as a scalable and practical platform for next-generation LIBs and ASSLBs.

## Experimental Section

### Material Preparation

Si/Sn composites with different Sn contents, denoted as Si/Sn-5, Si/Sn-10, Si/Sn-15, and Si/Sn-20 (corresponding to Si:Sn weight ratios of 95:5, 90:10, 85:15, and 80:20, respectively), were synthesized using high-power mechanical milling (MM) (SPEX 8000M). Stoichiometric amounts of Si powder (99.9%, AVENTION) and Sn powder (99%, Daejung Chemicals & Metals) were loaded into an 80 cm^3^ hardened steel vial with stainless steel balls (ball-to-powder weight ratio = 20:1). The vial was sealed under Ar to prevent oxidation, and milling was conducted for 6 h. To obtain Si/a-Sn/TMS composites (TMS = FeSi_2_, CoSi_2_, or NiSi_2_), Si, Sn, and the corresponding transition metal were milled under identical conditions. The starting powder amounts were adjusted to obtain a final composite comprising Si, Sn, and the in situ-formed TMS at a fixed weight ratio of 50:10:40 (as optimized). For comparison, a Sn-free Si/CoSi_2_ composite was prepared by milling Si and Co powders using the same high-power MM protocol, targeting a final Si:CoSi_2_ weight ratio of 60:40. The optimized Si/a-Sn/CoSi_2_ powder was mixed with graphite (mesocarbon microbeads, MCMB) at a weight ratio of 70:30 and briefly milled for 5 min to obtain Si/a-Sn/CoSi_2_/G. To obtain Si/a-Sn/CoSi_2_/G/C, the Si/a-Sn/CoSi_2_/G was mixed with poly(vinyl chloride) (PVC) and heat-treated at 700 °C for 3 h under Ar. The PVC amount was calculated based on the carbon yield determined by thermogravimetric analysis (TGA) to achieve 10 wt% PVC-pyrolyzed carbon (Si/a-Sn/CoSi_2_/G:C = 90:10 wt%). Individual TMS phases (FeSi_2_, CoSi_2_, and NiSi_2_) were synthesized by high-power MM of each transition metal (Fe: > 99% purity, Sigma-Aldrich; Co: > 99% purity, Kanto Chemical; Ni: 99% purity, Daejung Chemicals & Metals) with Si for 6 h under identical conditions. For ASSLB tests, argyrodite-type Li_6_PS_5_Cl (LPSC) was provided by Dongwha Electrolyte and used as received.

### Material Characterization

The crystal structures were characterized using X-ray diffraction (XRD) (Rigaku D/MAX, Cu Kα radiation) and Raman spectroscopy (Renishaw System 1000, 514 nm laser). Extended X-ray absorption fine structure (EXAFS) analysis at the Sn K-edge was conducted at the 10C wide XAFS beamline of the Pohang Accelerator Laboratory. The microstructures and elemental distributions were examined using transmission electron microscopy (TEM) (JEM-ARM200F, JEOL) and energy-dispersive X-ray spectroscopy (EDX). The mechanical properties of the synthesized TMS phases, graphite, and PVC-pyrolyzed carbon were evaluated using a nanoindenter (iMicro, KLA) with a 100-μm-diameter flat-punch tip. Nanoindentation tests were performed to a maximum depth of 1000 nm at an indentation strain rate of 0.05 s^–1^. Cyclic voltammetry (CV) measurements for the anodes were taken at a scan rate of 3 mV s^–1^ over a voltage range of 0.01–2.0 V (*vs.* Li^+^/Li). The electronic conductivity was determined from current–voltage (I–V) measurements using pelletized samples (area: 0.785 cm^2^) and a ZIVELAB electrochemical workstation by sweeping the voltage from –300 to + 300 mV. The Li-ion conductivity of the LPSC SE was measured using a KEITHLEY 6517B electrometer under a DC bias of 50 mV in symmetric Li|LPSC|Li cells, where a pelletized argyrodite LPSC was sandwiched between two Li foils. Electrochemical impedance spectroscopy (EIS) was conducted over a frequency range of 1 MHz–10 mHz with an AC amplitude of 10 mV. TGA (TGA550, TA Instruments) was performed under N_2_ at a heating rate of 10 °C min^–1^ up to 900 °C to determine the carbon yield from PVC. The surface and cross-sectional morphologies were examined using scanning electron microscopy (SEM) (SNE-4500M, SEC), and the particle size distributions were obtained using a particle size analyzer (PSA) (Mastersizer 3000, Malvern). For cross-sectional SEM observation, the samples were mechanically sectioned using a cutting tool inside an Ar-filled glovebox to expose fresh cross-sectional surfaces. The specific surface areas of the samples were measured by N_2_ adsorption–desorption isotherms using a Brunauer–Emmett–Teller (BET) analyzer (3Flex, Micromeritics). Prior to measurement, the samples were degassed under vacuum at 120 °C for 12 h. Electrode thickness changes before and after cycling were investigated using optical microscopy (OM) (BX53M, Olympus). X-ray photoelectron spectroscopy (XPS) (PHI 5000 VersaProbe III, ULVAC-PHI) with Al Kα radiation was utilized to investigate the chemical composition of LPSC SE.

### Electrochemical Measurements

For anode fabrication, poly(acrylic acid) (PAA) binder was dissolved in distilled water to prepare an aqueous solution, after which the active material and carbon black (Denka) were added to form a homogeneous slurry (active material:carbon black:PAA = 70:15:15 wt%). The slurry was coated onto Cu foil, vacuum-dried at 120 °C for 3 h, and punched into 10 mm disks with an active material loading of ~ 2.5 mg cm^–2^. LIB half-cell tests were performed using coin-type half-cells assembled in an Ar-filled glovebox (O_2_ and H_2_O < 0.5 ppm) with Li foil (MTI) as the counter/reference electrode. An electrolyte of 1.3 M LiPF_6_ in ethylene carbonate/diethyl carbonate (EC/DEC; 3:7 vol%) containing 10 wt% fluoroethylene carbonate (FEC) was used with a Celgard 2400 polypropylene separator. Galvanostatic cycling was conducted at 300 mA g^–1^ within a range of 0.01–2.0 V (*vs.* Li^+^/Li), and the rate capability was evaluated at various current densities. For LIB full-cells, cathodes were prepared by dissolving poly(vinylidene fluoride) (PVDF) in N-methyl-2-pyrrolidone (NMP), followed by the addition of NCM811 and carbon black to obtain a slurry (NCM811:carbon black:PVDF = 80:10:10 wt%), which was coated onto Al foil and dried at 120 °C for 3 h. Prior to the full-cell assembly, the anodes were prelithiated by direct contact in an Ar-filled glovebox at 25 °C by placing a Li foil in face-to-face contact with the anode and introducing a small amount of electrolyte at the interface to provide ionic conduction. The stack was then pressed at 1 MPa for 20 min to promote pressure-assisted Li insertion into the anode. The full-cells were tested with an N/P capacity ratio of 1.1 within a range of 2.0–4.3 V. All LIB measurements were taken at room temperature using a Maccor Series 4000 system. ASSLB tests were carried out using load-type cell molders (TLP1109-SR, Teraleader Inc.) equipped with hardened martensitic steel punches and a 10-mm-diameter polyetheretherketone (PEEK) spacer (Fig. S1). For ASSLB half-cell assembly, an argyrodite LPSC SE pellet (80 mg, thickness: ~ 600 μm) was prepared by uniaxial pressing at 125 MPa for 1 min. The anode was placed on one side of the pellet and consolidated at 375 MPa for 3 min. A Li–In counter/reference electrode (Li:In = 1:2, molar ratio) was prepared by pressing Li (50 μm) and In (100 μm) foils together at 62.5 MPa for 1 min. For ASSLB full-cells, a composite cathode was prepared by mechanically mixing NCM811, vapor-grown carbon fiber, and LPSC at 77:3:20 wt% under Ar. The composite cathode was placed on one side of the LPSC pellet, while the anode was placed on the opposite side, followed by consolidation of the entire stack at 375 MPa for 3 min. The N/P capacity ratio was fixed at 1.1. Electrochemical measurements of the ASSLB half-cells were taken at 60 °C (except for temperature-dependent tests) within a voltage range of –0.6 to 1.38 V versus Li–In, whereas ASSLB full-cells were tested at 60 °C (except for temperature-dependent tests) within a voltage range of 2.0–4.3 V. A constant operating stack pressure of 40 MPa was applied during cycling for both the half-cell and full-cell ASSLB tests. All ASSLB tests were conducted using a Maccor Series 4000 system.

## Results and Discussion

### Optimizing the Sn Matrix in Si/Sn Composite Anodes

**Fig. 1:**
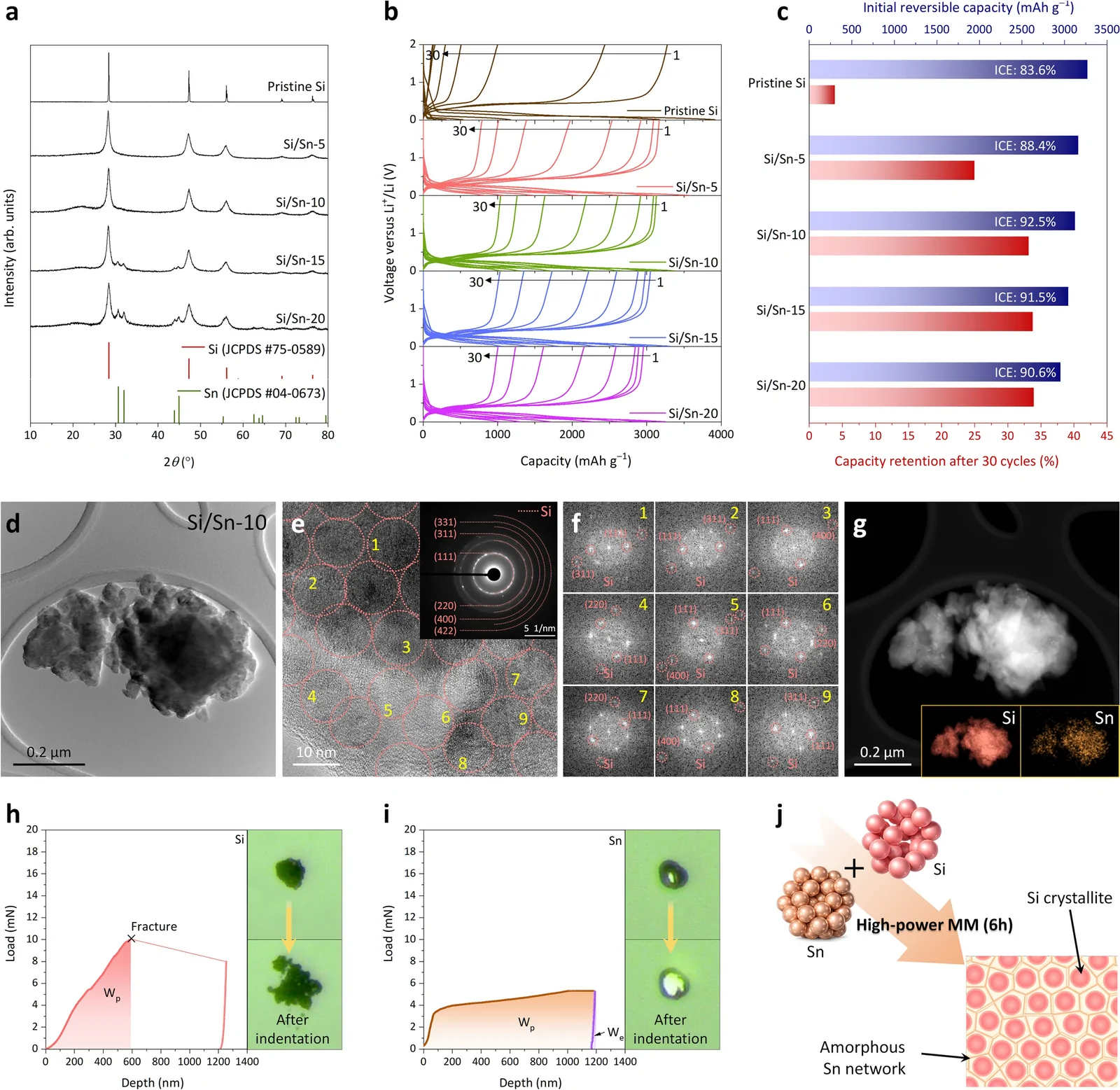
Structural and electrochemical characterization results for pristine Si and Si/Sn composite anodes. **a** XRD patterns and **b** voltage profiles obtained at 300 mA g^–1^ for pristine Si and Si/Sn composite anodes with Sn contents of 5, 10, 15, and 20 wt%. **c** Summary of initial reversible capacity, ICE, and capacity retention of pristine Si and Si/Sn composites after 30 cycles. **d** BFTEM image, **e** HRTEM image with SAED pattern (inset), **f** FFT patterns of selected regions in (e), and **g** STEM image and corresponding EDX elemental mapping of the Si/Sn-10 composite. Load–displacement profiles of **h** Si and **i** Sn obtained by nanoindentation. **j** Schematic illustration of the microstructure of the Si/Sn-10 composite synthesized by high-power MM

To mitigate the severe volume change and intrinsically low electronic conductivity of Si, metallic candidates such as Al, Zn, and Sn were selected based on their nonsilicide-forming behavior, high electronic conductivity, and sufficient mechanical deformability to serve as matrix components for Si. To compare their structural evolution under identical high-power MM conditions, each metal was incorporated at 10 wt%. The XRD results (Fig. S2) showed that only Sn existed in an amorphous state, enabling more homogeneous dispersion within Si, whereas Al and Zn remained as crystalline phases. This distinctive structural evolution indicates that Sn is more favorable for achieving fine and homogeneous dispersion within Si, which is advantageous for constructing an effective buffering matrix. Consistently, electrochemical comparisons showed that Si/Sn delivered the most favorable balance of high initial Coulombic efficiency (ICE) and reversible capacity, whereas Si/Al suffered from a relatively low initial efficiency, likely due to the native oxide layer on Al, and Si/Zn exhibited a lower reversible capacity because of the intrinsically low theoretical capacity of Zn. Therefore, Sn was selected as the optimal metallic component in this system based on these combined structural and electrochemical results. Based on this result, Si/Sn composites with various Sn contents (5–20 wt%) were further prepared using a high-power MM technique to optimize the composition. Distinct Sn diffraction peaks emerged in the XRD patterns when the Sn content exceeded 15 wt% (Fig. 1a), indicating the formation of crystalline Sn due to Sn coarsening. Electrochemical evaluation results (Fig. 1b) further demonstrated that incorporating 10 wt% Sn into the Si anode markedly enhances the electrochemical performance. This improvement is attributed to the effective dispersion of the well-deformable, electronically conductive Sn matrix, which facilitates enhanced electronic pathways. Based on the initial reversible capacity, ICE, and capacity retention after 30 cycles (Fig. 1c and Table S1), the optimal composition was determined to be 90 wt% Si and 10 wt% Sn (Si/Sn-10). When the Sn content exceeded 10 wt%, the excess Sn tended to undergo crystallite coarsening, as evidenced by the XRD results, thereby disrupting the formation of continuous electronically conductive pathways and resulting in reduced ICE and limited capacity retention [30]. TEM was performed to examine the microstructural characteristics. Bright-field TEM (BFTEM) revealed the overall particle morphology of the Si/Sn-10 composite (Fig. 1d), while high-resolution TEM (HRTEM) and selected-area electron diffraction (SAED) revealed that ~ 10 nm Si crystallites were uniformly dispersed within an amorphous Sn matrix (Fig. 1e). The Si crystallite size estimated from HRTEM results agreed well with the Scherrer-derived value (10.91 nm; Fig. S3). Fast Fourier transform (FFT) analyses of selected regions assigned the observed lattice spacings (*d*-spacings) exclusively to crystalline Si (Fig. 1f), further confirming the amorphous nature of the Sn matrix. Scanning TEM (STEM) and EDX elemental mapping confirmed the homogeneous distribution of Sn throughout the composite (Fig. 1g).

To elucidate the mechanical behaviors of Si and Sn in the composite, their mechanical responses were quantified using nanoindentation tests, which were conducted on ~ 20 μm particles of Si and Sn (maximum depth: 1000 nm; strain rate: 0.05 s^–1^; Fig. S4). The load–displacement profile of Si (Fig. 1h) exhibits an abrupt displacement excursion accompanied by particle fracture, as confirmed by OM images taken before and after indentation. The total deformation energy of Si was dominated by the plastic deformation energy (W_p_: 30.372 × 10^–10^ J), with no elastic deformation energy (W_e_), highlighting the highly brittle nature of Si. In contrast, Sn sustained indentation to 1000 nm without fracture at a maximum load of only 5.3 mN (Fig. 1i), and its deformation energy was predominantly W_p_ (51.603 × 10^–10^ J) with negligible W_e_ (0.497 × 10^–10^ J), indicating pronounced deformability. Based on these contrasting mechanical characteristics, during the high-power MM process, Sn preferentially deformed and spread along the surfaces of the Si nanocrystallites, forming an interconnected amorphous Sn network between them (Fig. 1j). This well-deformable and electronically conductive amorphous Sn network contributed to the enhanced electrochemical performance. However, the capacity retention of the Si/Sn-10 anode after 30 cycles remained poor (~ 33.2%), indicating that additional structural stabilization was still required for long-term cycling.

### One-Pot Mechanochemical Synthesis of Si/a-Sn/TMS Composites

**Fig. 2:**
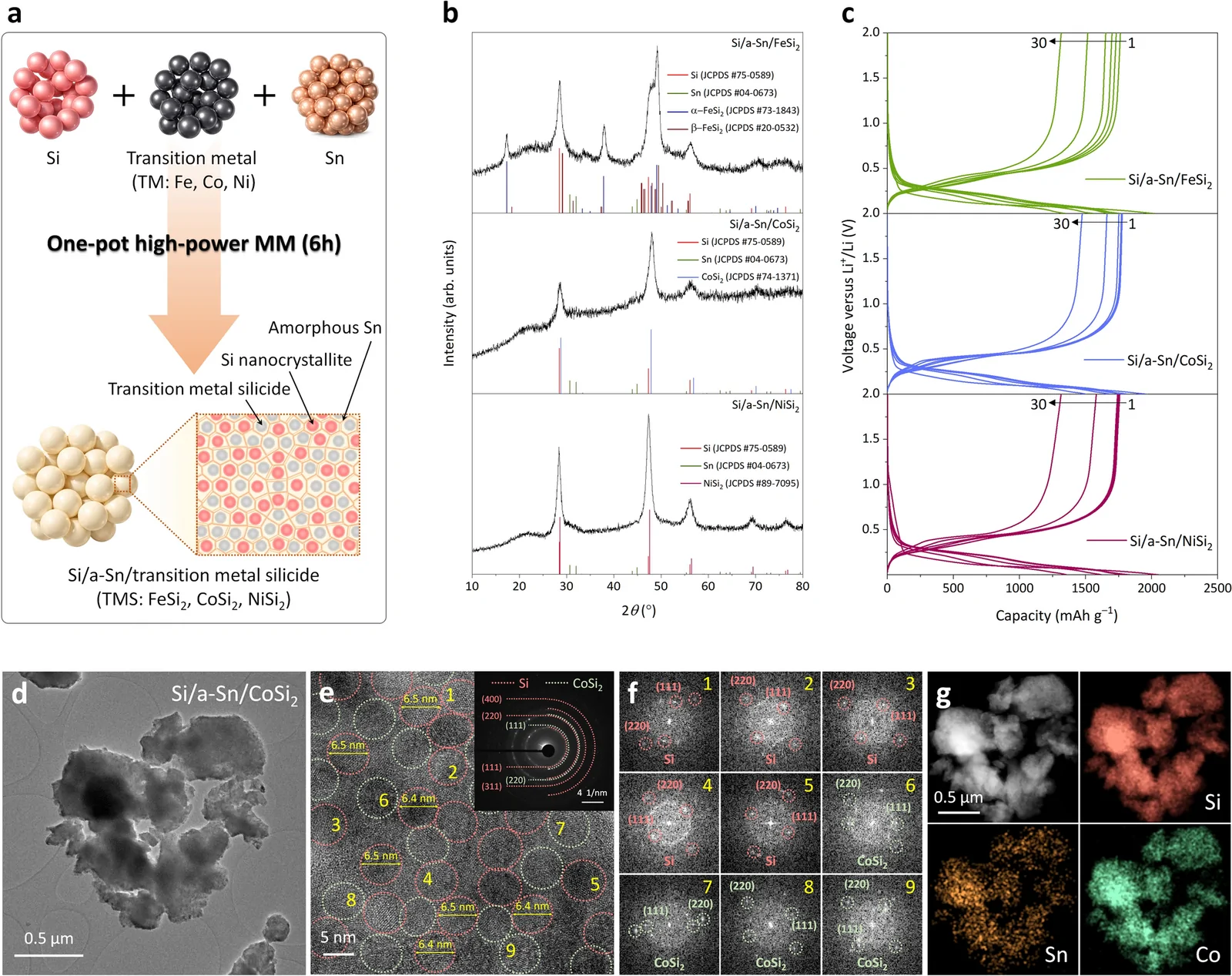
Synthesis, structural characterization results, and electrochemical performances of Si/a-Sn/TMS composites (TMS = FeSi_2_, CoSi_2_, and NiSi_2_). **a** Schematic illustration of the fabrication process via one-pot high-power MM-driven mechanochemical synthesis. **b** XRD patterns of the Si/a-Sn/FeSi_2_, Si/a-Sn/CoSi_2_, and Si/a-Sn/NiSi_2_ composites. **c** Voltage profiles of the Si/a-Sn/TMS composite anodes tested at 300 mA g^–1^. **d** BFTEM image, **e** HRTEM image with SAED pattern (inset), **f** FFT patterns of selected regions in (e), and **g** STEM image and corresponding EDX elemental mapping of the Si/a-Sn/CoSi_2_ composite

To further enhance the cycling stability of the Si/Sn-10 anode, TMS phases were incorporated as electronically conductive and mechanically robust matrices using a one-pot high-power MM process. As schematically illustrated in Fig. 2a, co-milling Si, Sn (10 wt%), and a transition metal precursor (Fe, Co, or Ni) yielded three distinct Si/a-Sn/TMS composites (TMS = FeSi_2_, CoSi_2_, or NiSi_2_). The XRD patterns (Fig. 2b) confirmed the successful formation of the corresponding TMS phases, along with reduced crystalline Si and amorphous Sn. PSA and SEM images confirmed that the average particle sizes of the Si/a-Sn/FeSi_2_, Si/a-Sn/CoSi_2_, and Si/a-Sn/NiSi_2_ nanocomposites were 10.0, 9.35, and 10.4 μm, respectively (Fig. S5). Electrochemical tests (Fig. 2c and Table S2) showed that all Si/a-Sn/TMS composite anodes outperformed the Si/Sn-10 anode, demonstrating the effectiveness of the electronically conductive and mechanically robust TMS matrix. Among them, Si/a-Sn/CoSi_2_ delivered the most balanced performance, achieving an initial discharge/charge capacity of 1961.7/1771.5 mAh g^–1^ (ICE: 90.3%) and a capacity retention of 83.2% after 30 cycles. Furthermore, to elucidate the role of Sn, a Sn-free Si/CoSi_2_ composite was prepared (Fig. S6). Compared with the Si/a-Sn/CoSi_2_ anode, it had an inferior electrochemical performance, including a lower ICE (86.5%), poorer capacity retention (77.6% after 30 cycles), and a reduced average Coulombic efficiency (CE) over 30 cycles (97.2% *vs.* 98.5% for Si/a-Sn/CoSi_2_; Fig. S7), underscoring the critical role of a uniformly dispersed, electronically conductive amorphous Sn matrix in ensuring stable cycling.

TEM analyses further elucidated the microstructures of the Si/a-Sn/TMS composites (Figs. 2d–g and S8–S9). For the Si/a-Sn/CoSi_2_ composite, BFTEM (Fig. 2d) revealed the overall morphology, whereas HRTEM (Fig. 2e) showed densely packed nanocrystallites. Although Si and CoSi_2_ have similar cubic crystal structures with closely matched d-spacings, local FFT analyses of selected regions enabled phase identification (Fig. 2f). The measured crystallite sizes (6.3–6.8 nm) of both the Si and CoSi_2_ were consistent with the Scherrer-estimated value (~ 6.39 nm; Fig. S10b). STEM–EDX elemental mapping further confirmed the homogeneous distribution of nanocrystalline Si and CoSi_2_, with amorphous Sn uniformly surrounding the crystallites to form a continuous electronically conductive amorphous matrix that facilitated efficient charge transport (Fig. 2g). Similar TEM analyses were performed for the Si/a-Sn/FeSi_2_ (Fig. S8) and Si/a-Sn/NiSi_2_ composites (Fig. S9). The crystallite sizes determined from the Scherrer equation were in good agreement with the HRTEM measurements (Fig. S10), confirming the well-dispersed nanocrystalline Si and TMS phases embedded within a uniformly distributed amorphous Sn matrix.

To clarify the role of the TMS phases in the electrochemical performance of the composites, individual TMS phases were synthesized (Fig. S11). Their electronic conductivities were quantified by I–V measurements over a range of − 300 to + 300 mV using a SUS|TMS pellet|SUS symmetric cell configuration (Fig. S12 a). All the TMS phases exhibited significantly higher electronic conductivities than that of Si (1.177 × 10^–5^ S cm^–1^). Among them, CoSi_2_ (2.186 × 10^–1^ S cm^–1^) and NiSi_2_ (1.950 × 10^–1^ S cm^–1^) showed particularly high electronic conductivities, enabling efficient electron transport and thereby enhancing charge transfer kinetics and rate capability. In contrast, FeSi_2_ exhibited the lowest electronic conductivity (1.573 × 10^–2^ S cm^–1^), which could be attributed to its semiconducting nature [31]. In addition, the mechanical properties of the TMS phases were evaluated by nanoindentation tests (Fig. S13 and Table S3). All TMS particles were indented to a maximum depth of 1000 nm without fracture, and OM images taken before and after the indentation confirmed that their morphologies were well preserved (inset in Fig. S13), demonstrating their intrinsic mechanical robustness. Among the TMS phases, CoSi_2_ exhibited the highest total deformation energy (W_total_: 177.967 × 10^–10^ J), comprising 68% W_p_ and 32% W_e_, indicating its superior tolerance to severe mechanical stress and strong capability for elastic recovery. The substantial plastic energy dissipation, combined with elastic strain accommodation, enables CoSi_2_ to effectively buffer Si volume expansion while maintaining structural coherence. Electrochemical evaluation of the TMS anodes, including FeSi_2_, CoSi_2_, and NiSi_2_, showed negligible reversible capacities, which originated from the conductive carbon additive (approximately 40 mAh g^–1^; Fig. S14), confirming their electrochemical inactivity toward Li. Therefore, CoSi_2_ primarily serves as a mechanically robust and elastic framework, while also providing electronic conductivity within the composite. This mechanically robust and elastically recoverable behavior, together with its high electronic conductivity, established a conductivity–mechanical synergy that accounted for the superior electrochemical performance of the Si/a-Sn/CoSi_2_ composite.

### Graphite Scaffold as a Li-Reversible Conductive and Stress-Mitigating Matrix

**Fig. 3:**
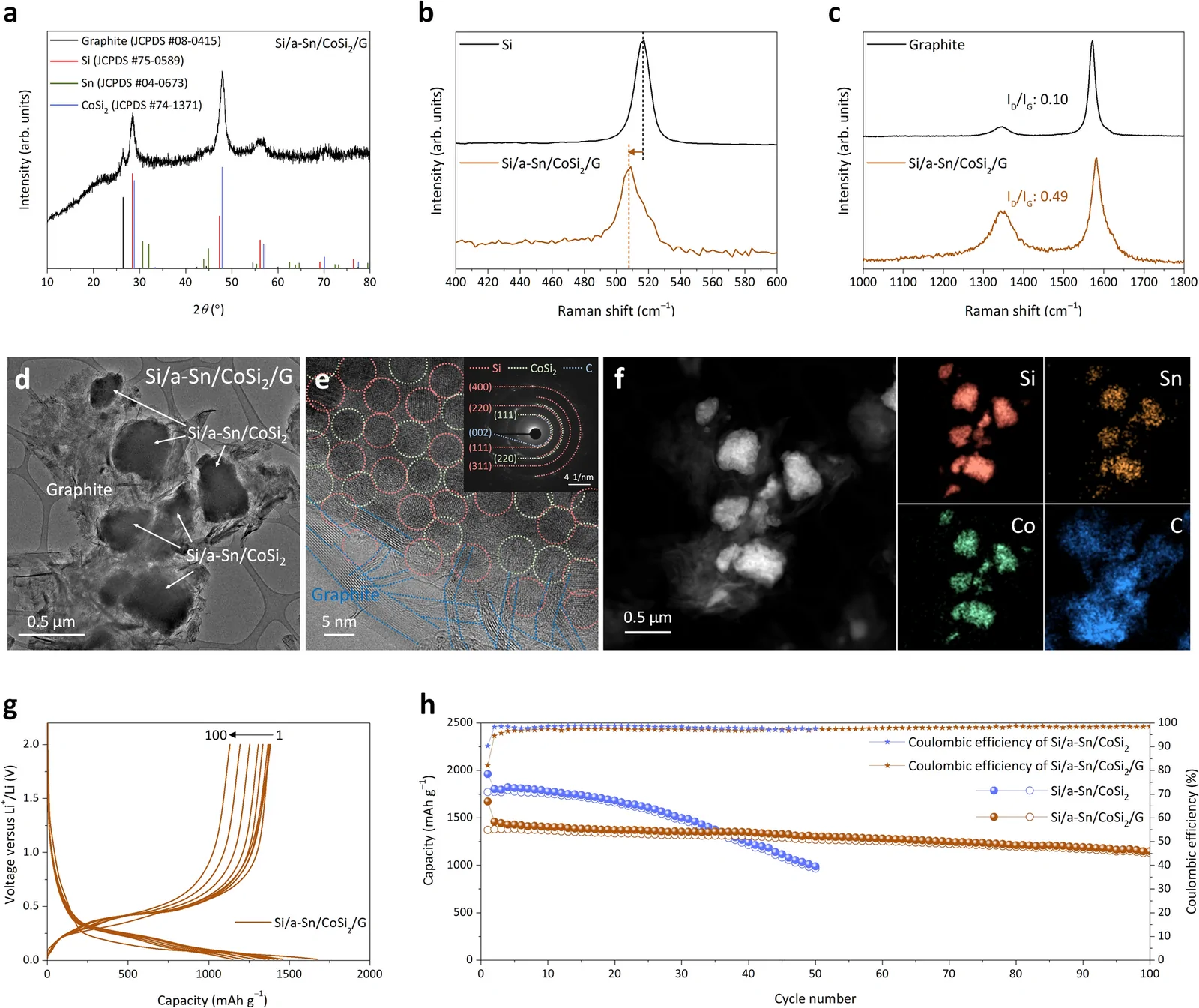
Structural characterization and electrochemical performance of the Si/a-Sn/CoSi_2_/G composite. **a** XRD pattern of the Si/a-Sn/CoSi_2_/G composite. **b** Raman spectra of the pristine Si and Si/a-Sn/CoSi_2_/G composite (Raman shift: 400–600 cm^–1^). **c** Raman spectra of graphite and the Si/a-Sn/CoSi_2_/G composite (Raman shift: 1000–1800 cm^–1^). **d** BFTEM image, **e** HRTEM image with SAED pattern (inset), and **f** STEM image with corresponding EDX elemental mapping of the Si/a-Sn/CoSi_2_/G composite. **g** Voltage profile of the Si/a-Sn/CoSi_2_/G composite anode tested at 300 mA g^–1^. **h** Cycling performance comparison of the Si/a-Sn/CoSi_2_ and Si/a-Sn/CoSi_2_/G composite anodes

To further improve the long-term cycling stability and structural durability of the Si/a-Sn/CoSi_2_ composite, a highly Li-reversible, electronically conductive, and stress-mitigating graphite scaffold was incorporated using a simple high-power MM process (5 min) to form the Si/a-Sn/CoSi_2_/G nanocomposite. The XRD pattern (Fig. 3a) confirmed the coexistence of nanocrystalline Si, CoSi_2_, and graphite without detectable side products, indicating the successful formation of the graphite-incorporated composite. PSA and SEM revealed an average particle size of ~ 10.3 μm (Fig. S15). Raman spectroscopy showed a shift of the characteristic Si peak from 517.2 to 509.0 cm^–1^ (Fig. 3b), which was consistent with phonon confinement effects arising from Si nanocrystallization. Meanwhile, the I_D_/I_G_ ratio of graphite increased from 0.10 in the pristine state to 0.49 in the composite (Fig. 3c), reflecting increased structural disorder generated during high-power MM. These structural defects could provide additional electrochemically active sites and facilitate Li⁺ transport along the graphite scaffold, thereby enhancing the reaction kinetics and structural adaptability. BFTEM (Fig. 3d) revealed that the Si/a-Sn/CoSi_2_ nanocomposite particles were uniformly anchored within the graphite scaffold, whereas HRTEM (Fig. 3e) showed well-dispersed Si and CoSi_2_ nanocrystallites (5–7 nm) within the nanocomposite, with the graphite scaffold providing an extended conductive network that bridged the nanocrystallites. Such an architecture is expected to enhance electronic percolation and provide mechanical buffering against Si volume changes during cycling. STEM and corresponding EDX elemental mapping (Fig. 3f) confirmed the presence of homogeneous distributions of Si, Co, and Sn within the Si/a-Sn/CoSi_2_ particles and their intimate integration with the graphite scaffold. The elastic recoverability of the graphite scaffold was further validated by the nanoindentation results, which exhibited fracture-free deformation with a high W_e_ contribution (38.4%) and preserved particle morphology (Fig. S16). Consistent with this structural resilience, electrochemical measurements (Fig. 3g) demonstrated that the Si/a-Sn/CoSi_2_/G anode delivered high initial discharge/charge capacities of 1672.9/1372.7 mAh g⁻^1^ (ICE: 82.1%) and retained 1131.7 mAh g⁻^1^ after 100 cycles, corresponding to a capacity retention of 82.4% (Fig. 3h).

### Highly Elastic PVC-Pyrolyzed Amorphous Carbon Shell for Structurally Stable, Fast Transport Nanocomposites

**Fig. 4:**
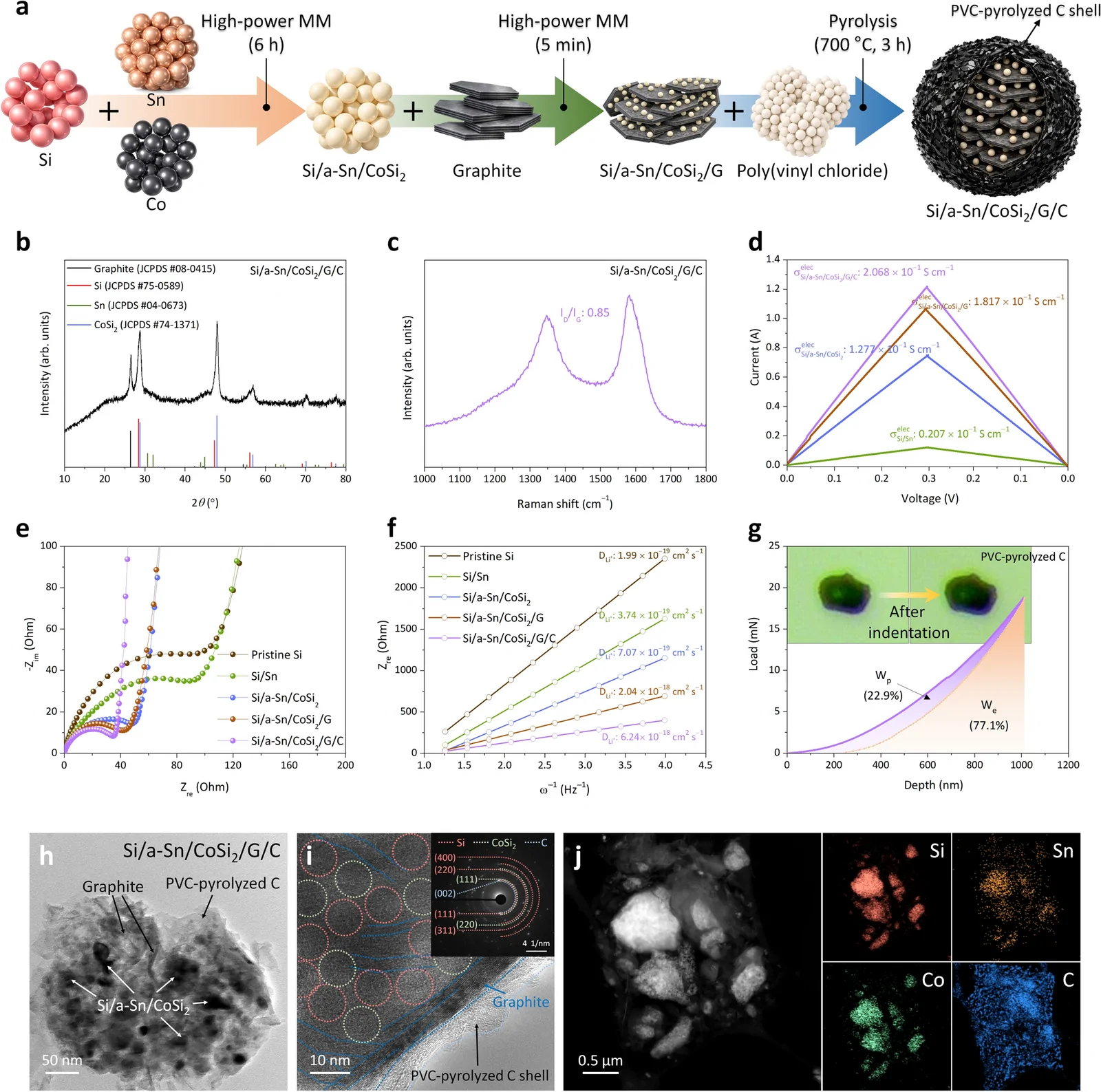
Synthesis, structural characterization, and charge transport properties of the Si/a-Sn/CoSi_2_/G/C nanocomposite. **a** Schematic illustration of the synthesis procedure for the Si/a-Sn/CoSi_2_/G/C nanocomposite. **b** XRD pattern and **c** Raman spectrum of Si/a-Sn/CoSi_2_/G/C. **d** I–V profiles of the as-prepared anode pellets. **e** Nyquist plots of the pristine Si, Si/Sn, Si/a-Sn/CoSi_2_, Si/a-Sn/CoSi_2_/G, and Si/a-Sn/CoSi_2_/G/C anodes. **f** Z_re_ versus ω^–1/2^ plots derived from (e). **g** Load–displacement profile of a PVC-pyrolyzed carbon particle. **h** BFTEM image, **i** HRTEM image with the corresponding SAED pattern (inset), and **j** STEM image with corresponding EDX elemental mappings of Si/a-Sn/CoSi_2_/G/C

Although the Si/a-Sn/CoSi_2_/G composite demonstrated a promising electrochemical performance, its long-term cycling stability remained insufficient for practical applications, motivating further reinforcement of the electrode structure. To address this limitation, a surface carbon coating was introduced using PVC as a cost-effective and scalable carbon precursor capable of yielding a dense, electronically conductive amorphous carbon shell upon pyrolysis [32]. The synthesis process for the final Si/a-Sn/CoSi_2_/G/C nanocomposite is schematically illustrated in Fig. 4a. Despite the stepwise procedure, the overall synthesis remains simple and scalable, consisting of only high-power MM and a single heat treatment step, both of which rely on straightforward and industry-compatible techniques. TGA of PVC (Fig. S17) revealed a residual carbon yield of ~ 13.5 wt% after pyrolysis at 700 °C. Based on this result, the PVC-pyrolyzed carbon content in the final composite was fixed at 10 wt%. The PVC-pyrolyzed carbon anode delivered a reversible capacity of 401.4 mAh g^–1^ with stable cycling behavior (Fig. S18), indicating its contribution to Li storage. In addition, I–V measurements confirmed the electronic conductivity of the PVC-pyrolyzed carbon (Fig. S19), supporting its role as an electronically conductive carbon shell. XRD patterns showed the presence of Si, CoSi_2_, and graphite with no detectable impurity phases (Fig. 4b), confirming phase retention during the PVC-pyrolyzed carbon coating process. SEM and PSA revealed a uniform particle morphology with an average particle size of 11.8 μm (Fig. S20). Raman spectroscopy further showed that the I_D_/I_G_ ratio increased from 0.49 for the uncoated Si/a-Sn/CoSi_2_/G to 0.85 after coating (Fig. 4c), indicating an increase in the structural disorder consistent with the formation of an amorphous carbon shell, as demonstrated by the XRD pattern of the PVC-pyrolyzed carbon (Fig. S21). Raman analysis confirmed the preservation of nanocrystalline Si, with the characteristic Si peak observed at 509.7 cm^–1^ (Fig. S22).

Electronic conductivity measurements (Fig. 4d) showed that the Si/a-Sn/CoSi_2_/G/C nanocomposite reached 2.068 × 10^–1^ S cm^–1^, the highest among all the tested composites. This remarkable enhancement can be attributed to the continuous electronic percolation network constructed by the conductive amorphous Sn matrix, CoSi_2_ framework, graphite scaffold, and outer amorphous carbon shell. Consistently, EIS (Fig. 4e) revealed that the Si/a-Sn/CoSi_2_/G/C anode had the lowest charge transfer resistance (R_ct_: 38.7 Ω), which was substantially lower than those of the pristine Si (143.9 Ω), Si/a-Sn (124.8 Ω), Si/a-Sn/CoSi_2_ (59.3 Ω), and Si/a-Sn/CoSi_2_/G (47.5 Ω). In addition, the Warburg factor (σ), which was obtained from the linear region of Z′ versus ω^–1/2^ (Fig. 4f), was used to calculate the Li^+^ diffusion coefficient (D_Li⁺_) using Eqs. (1) and (2):

1 $${Z}^{\prime}={R}_{1}+{R}_{ct}+\sigma {\omega }^{-1/2}$$

2 $$D_{Li^+} = \frac{{R^{2} T^{2} }}{{2A^{2} n^{2} F^{4} C^{2} \sigma^{2} }}$$

where Z' is the real part resistance, ω is the angular frequency, R is the gas constant, T is the absolute temperature, A is the surface area of the electrode (assumed to be a planar electrode), F is the Faraday constant, and C is the molar concentration of Li ions in the active material. The D_Li⁺_ value for Si/a-Sn/CoSi_2_/G/C reached 6.24 × 10^–18^ cm^2^ s^–1^, which was an order of magnitude higher than that of pristine Si (1.99 × 10^–19^ cm^2^ s^–1^) and markedly higher than those of Si/a-Sn (3.74 × 10^–19^ cm^2^ s^–1^), Si/a-Sn/CoSi_2_ (7.07 × 10^–19^ cm^2^ s^–1^), and Si/a-Sn/CoSi_2_/G (2.04 × 10^–18^ cm^2^ s^–1^). To clarify the role of the PVC-pyrolyzed carbon shell in structural stabilization, its mechanical response was evaluated by nanoindentation (Fig. 4g). The PVC-pyrolyzed carbon exhibited fracture-free deformation, with an elastic energy (W_e_) of 51.857 × 10^–10^ J and a plastic energy (W_p_) of 15.403 × 10^–10^ J, corresponding to a high elastic energy fraction of 77.1%, indicating strong elastic recoverability and effective accommodation of volume-change-induced mechanical stress. BFTEM and HRTEM with SAED patterns confirmed that the Si/a-Sn/CoSi_2_/G was coated with a thin PVC-pyrolyzed carbon shell (Fig. 4h, i). STEM and corresponding EDX mapping (Fig. 4j) further verified homogeneous distributions of the nanocrystalline Si, amorphous Sn, CoSi_2_ framework, graphite scaffold, and amorphous carbon shell.

### Electrochemical Performance of Si/a-Sn/CoSi_2_/G/C Nanocomposite Anodes in LIB Half Cells

**Fig. 5:**
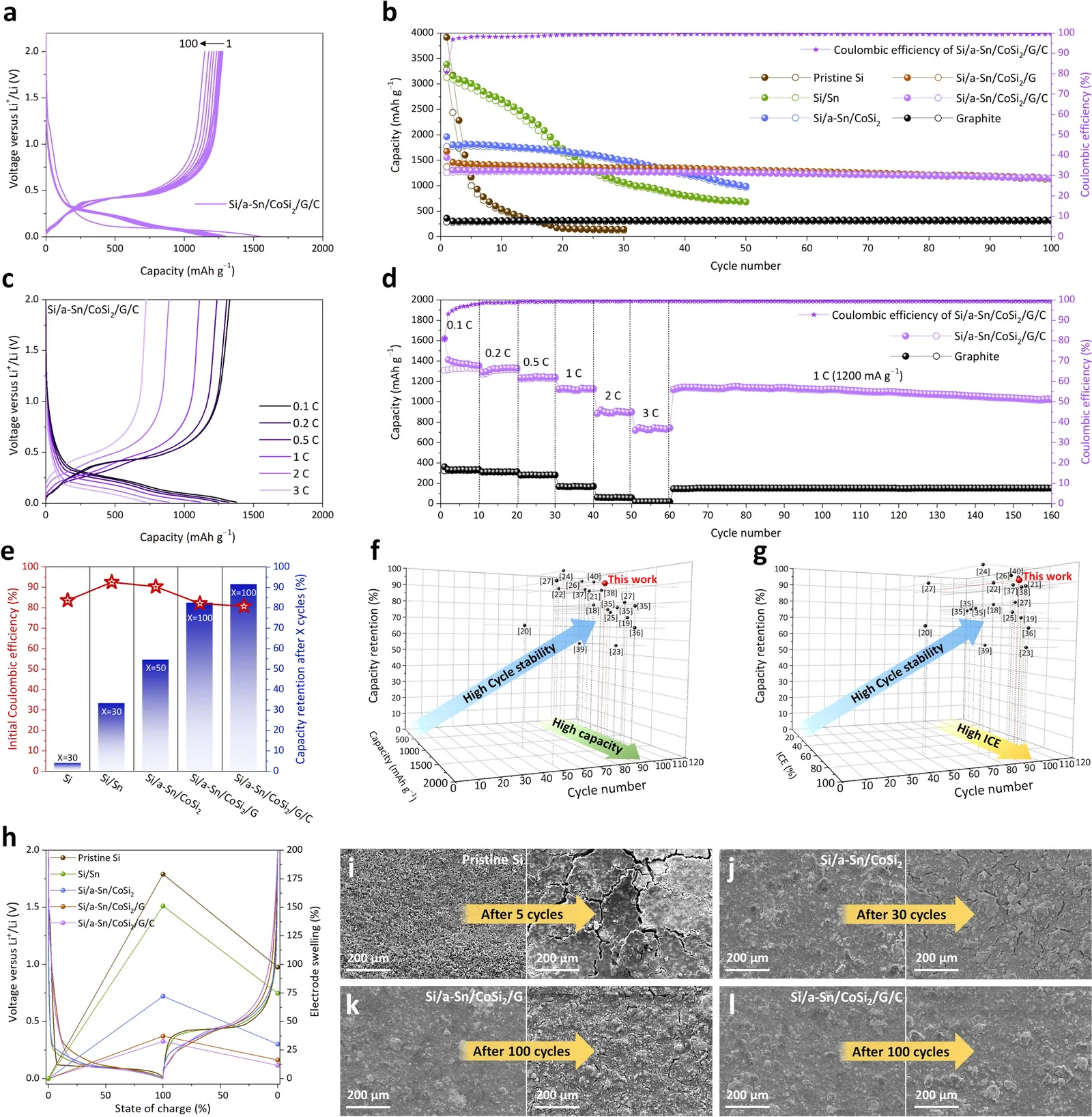
Electrochemical performance and structural stability of the Si/a-Sn/CoSi_2_/G/C anode. **a** Voltage profiles and **b** cycling performance of the Si/a-Sn/CoSi_2_/G/C anode tested at 300 mA g^–1^. **c** Voltage profiles and **d** rate capability test results for Si/a-Sn/CoSi_2_/G/C anode at various C-rates (1 C: 1200 mA g^–^^1^). **e** Summary of ICE and capacity retention results for Si/a-Sn/CoSi_2_/G/C and comparison anodes. Performance comparison of reported Si/TMS-based anodes and Si/a-Sn/CoSi_2_/G/C: **f** cycle number versus reversible capacity versus capacity retention and **g** cycle number versus ICE versus capacity retention. **h** Thickness variation at different states of charge (SOCs). Cross-sectional SEM images before and after cycling: **i** pristine Si (after 5 cycles), **j** Si/a-Sn/CoSi_2_ (after 30 cycles), **k** Si/a-Sn/CoSi_2_/G (after 100 cycles), and **l** Si/a-Sn/CoSi_2_/G/C (after 100 cycles)

Long-term cycling and rate capability tests were performed to evaluate the electrochemical performance of the Si/a-Sn/CoSi_2_/G/C nanocomposite as a LIB anode. Si/a-Sn/CoSi_2_/G/C delivered initial discharge/charge capacities of 1555.1/1255.4 mAh g^–1^ at 300 mA g^–1^ (ICE: 80.7%; Fig. 5a) and retained 1147.4 mAh g^–1^ after 100 cycles, which was a 91.4% capacity retention (Fig. 5b), markedly outperforming commercial graphite anodes (Figs. 5b and S23). To further clarify the electrochemical behavior of the composite, additional CV measurements were taken on Si, Sn, graphite, PVC-pyrolyzed carbon, and the Si/a-Sn/CoSi_2_/G/C nanocomposite (Fig. S24). During lithiation, the strong cathodic feature at ~ 0.01 V is mainly associated with the alloying of Si, together with Li intercalation into the carbonaceous components. The cathodic contribution of Sn lithiation is not clearly separated in the nanocomposite, which is likely due to the relatively low fraction and amorphous nature of Sn, as well as the overlap of its alloying signal with the broader cathodic response of Si and carbon. Upon delithiation, the anodic peaks at ~ 0.35 and 0.54 V are attributed to the dealloying of Si [4], while the peaks at ~ 0.66, 0.73, and 0.80 V correspond to the stepwise dealloying of Li_*x*_Sn phases toward metallic Sn [30]. In addition, graphite and PVC-pyrolyzed carbon contribute a broad anodic feature in the 0.2–0.35 V region, which is attributed to Li deintercalation from the carbonaceous components. Overall, the CV results support the reversible electrochemical behavior of Si, Sn, and carbonaceous components in the Si/a-Sn/CoSi_2_/G/C nanocomposite. To identify the phase evolution of the nanocomposite, ex situ XRD analysis was conducted (Fig. S25). At the fully lithiated state (0.01 V), characteristic diffraction peaks assignable to Li_3.75_Si and LiC_6_ are observed, confirming the lithiation of both Si and graphite components. Although Sn-related lithiated phases are not clearly detected in the XRD patterns due to their low content and amorphous nature, Sn K-edge EXAFS analysis (Figs. S26b and S27) reveals the formation of Li_4.4_Sn at 0.01 V, which reversibly transforms back to metallic Sn upon delithiation (2.0 V). In addition, the CoSi_2_ phase remains structurally unchanged during cycling, as evidenced by the invariant diffraction peaks (Fig. S25), indicating its electrochemical inactivity and role as a mechanically robust matrix. Ex situ EIS analysis (Fig. S28) shows that R_int_ remains nearly constant, indicating the formation of a stable interphase without significant continuous degradation. In contrast, R_ct_ decreases from 38.7 Ω (pristine) to 26.1, 22.8, and 22.3 Ω after 10, 50, and 100 cycles, respectively, indicating progressively improved charge transfer kinetics. This behavior can be attributed to multiple synergistic effects during cycling: (i) electrode activation, which increases the electrochemically active interfacial area; (ii) the mechanically robust and elastically buffering multimatrix architecture that maintains intimate interparticle contact, suppresses crack-induced isolation, and mitigates structural degradation during repeated volume changes; and (iii) enhanced electronic percolation through stable interparticle contacts and reduced local contact resistance within the preexisting Sn and carbon conductive network. Rate capability tests demonstrate outstanding high-rate performance, delivering reversible capacities of 1328.2, 1307.3, 1236.4, 1109.8, 888.7, and 725.2 mAh g^–1^ at 0.1, 0.2, 0.5, 1, 2, and 3 C (1 C: 1200 mA g^–1^), respectively (Fig. 5c, d). Notably, the capacity fully recovered upon returning to 1 C following high-rate cycling at 3 C, accompanied by stable cycling behavior, confirming the high reversibility and fast reaction kinetics. Among the anodes compared, Si/a-Sn/CoSi_2_/G/C simultaneously achieved a high ICE and the highest capacity retention (Fig. 5e). To further elucidate the origin of the high ICE, the specific surface areas (SSA) of the samples were analyzed (Fig. S29). The SSA evolution generally correlated with the ICE behavior [33, 34]. Sn incorporation lowered the SSA of Si and improved the ICE, indicating reduced exposed reactive surface area and suppressed electrolyte decomposition. Although the Si/a-Sn/CoSi_2_ composite showed a slightly increased SSA due to particle fragmentation induced by high-power MM, it still delivered a high ICE, suggesting that ICE is governed not solely by SSA but also by the structural and interfacial characteristics of the composite. After graphite incorporation, the SSA increased markedly with a decrease in ICE, whereas subsequent PVC-pyrolyzed carbon coating reduced the SSA, thereby helping to stabilize the ICE. Overall, the ICE behavior is determined by the combined effects of surface area, surface passivation, and structural stability within the multimatrix architecture. Furthermore, it outperformed most previously reported Si/TMS-based anodes in terms of cycle life, capacity retention, ICE, and reversible capacity (Fig. 5f, g and Table S4) [18–27, 35–40]. The composite composition was intentionally designed to achieve a balance between capacity and electrochemical stability for practical Si-based anodes. Such performance originates from the hierarchical conductive–elastic architecture of the Si/a-Sn/CoSi_2_/G/C nanocomposite anode, which integrates ultrafine Si with a well-deformable amorphous Sn matrix, a mechanically robust and elastic CoSi_2_ framework, a Li-reversible graphite scaffold, and a PVC-pyrolyzed amorphous carbon shell. The synergistic interaction among these components establishes efficient electron/ion transport pathways, as evidenced by the increased electronic conductivity, reduced charge transfer resistance, and enhanced Li-ion diffusion coefficient (Fig. 4d–f).

Electrode swelling measurements and corresponding SEM analyses further clarified the structural evolution of the pristine Si, Si/a-Sn/CoSi_2_, Si/a-Sn/CoSi_2_/G, and Si/a-Sn/CoSi_2_/G/C anodes (Fig. 5h–l). The Si/a-Sn/CoSi_2_/G/C anode showed only 32.6% swelling in the fully lithiated state, which was substantially lower than the values for pristine Si (179.1%), Si/Sn-10 (151.2%), Si/a-Sn/CoSi_2_ (72.1%), and Si/a-Sn/CoSi_2_/G (37.2%). Upon delithiation, the expansion decreased to 11.6%, indicating a highly reversible volume change (Figs. 5h and S30). Consistently, the pristine Si exhibited severe pulverization and cracking after only five cycles (Fig. 5i), whereas Si/a-Sn/CoSi_2_ mitigated damage after 30 cycles (Fig. 5j), and the addition of graphite further suppressed cracking after 100 cycles (Fig. 5k). Notably, Si/a-Sn/CoSi_2_/G/C maintained a crack-free, intact morphology even after 100 cycles (Fig. 5l), indicating that the multimatrix architecture effectively accommodates cycling-induced strain and enables stress redistribution across multiple components, thereby suppressing volume expansion and preventing crack formation.

### Practical High-Energy LIB Full-Cell Performance Enabled by the Si/a-Sn/CoSi_2_/G/C Nanocomposite Anode

**Fig. 6:**
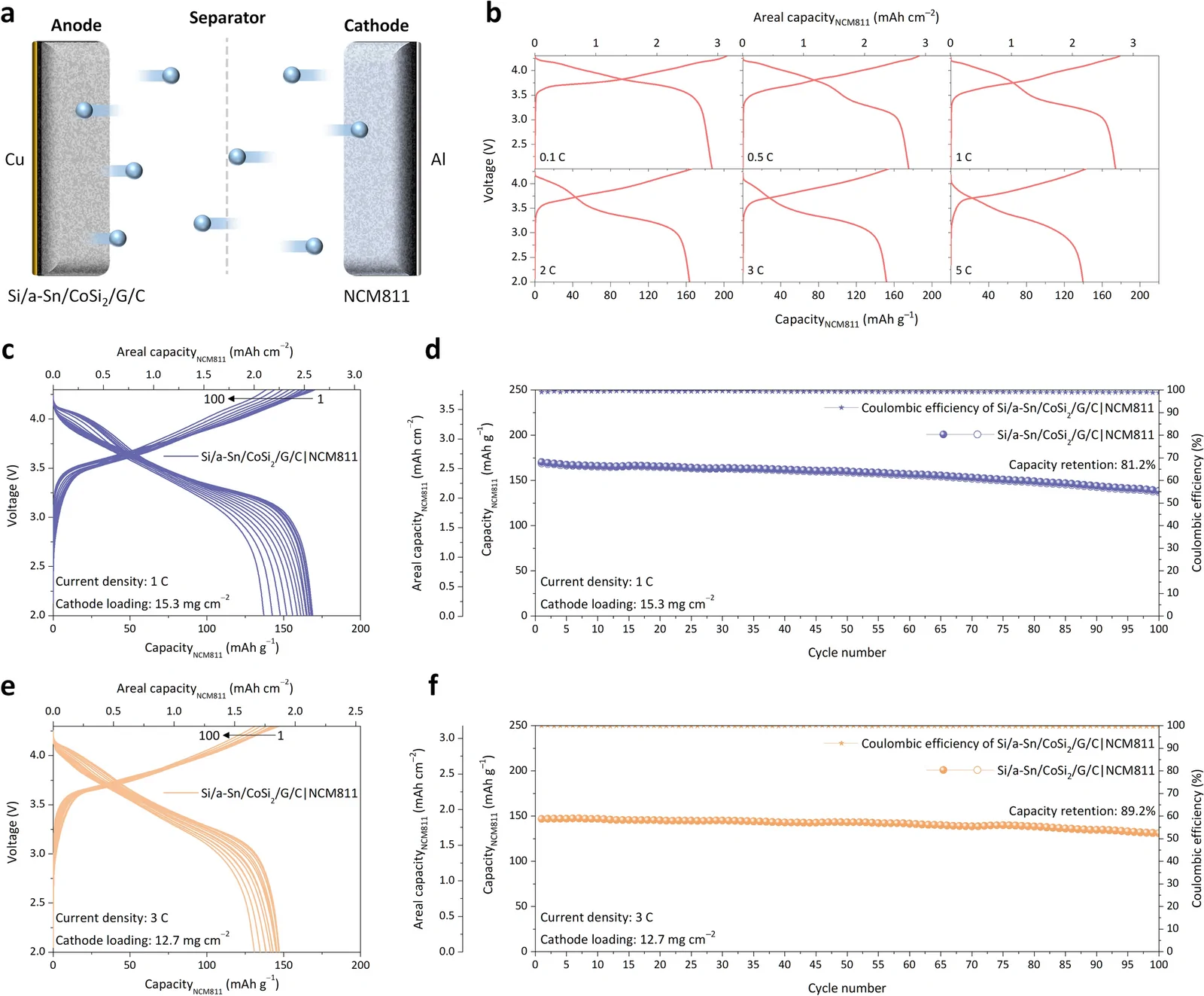
Electrochemical performance of the Si/a-Sn/CoSi_2_/G/C|NCM811 LIB full-cell. **a** Schematic illustration of the full-cell configuration employing a Si/a-Sn/CoSi_2_/G/C anode and an NCM811 cathode. **b** Voltage profiles at various current densities from 0.1 to 5 C (cathode loading: 15.5 mg cm^–2^). **c** Voltage profiles and **d** long-term cycling performance at 1 C (cathode loading: 15.3 mg cm^–2^). **e** Voltage profiles and **f** long-term cycling performance at 3 C (cathode loading: 12.7 mg cm^–2^)

To assess its practical applicability, a LIB full-cell was assembled by pairing the Si/a-Sn/CoSi_2_/G/C anode with an NCM811 cathode (Fig. 6a). In a rate capability test with a high cathode loading (15.5 mg cm^–2^; Fig. 6b), the cell delivered discharge capacities of 187.4, 175.5, 174.2, 163.6, 151.9, and 139.7 mAh g^–1^ at 0.1, 0.5, 1, 2, 3, and 5 C, respectively. The average discharge voltage (V_ave_) was calculated using the integral method (V_ave_ = (∫VdQ)/Q_total_). Based on this value, the corresponding energy densities were calculated using the combined mass of the anode, cathode, and separator, as summarized in Table S5, reaching 434.4 Wh kg^–1^ at 0.1 C. For comparison, a graphite|NCM811 full-cell was assembled and evaluated under identical conditions (Fig. S31). The graphite-based full-cell delivered lower discharge capacities of 179.0, 152.5, 140.2, 124.8, 112.2, and 88.1 mAh g^–1^ at 0.1, 0.5, 1, 2, 3, and 5 C, respectively, corresponding to substantially lower energy densities across the entire current density range, including 309.1 Wh kg^–1^ at 0.1 C (Table S5). These results highlight the advantage of the Si/a-Sn/CoSi_2_/G/C nanocomposite anode over the conventional graphite anode for high-energy LIB full-cells. Long-term cycling was further evaluated at 1 C and 3 C (1 C: 180 mA g^–1^, based on the cathode mass) after an initial formation cycle (Fig. S32). The full-cells exhibited ICE values of 78.0% and 78.1% at cathode loadings of 15.3 and 12.7 mg cm^–2^, respectively. At 1 C with a cathode loading of 15.3 mg cm^–2^, the full-cell delivered 137.1 mAh g^–1^ (areal capacity: 2.10 mAh cm^–2^) after 100 cycles, corresponding to a capacity retention of 81.2% (Fig. 6c, d). Even at 3 C with a cathode loading of 12.7 mg cm^–2^, it maintained 130.9 mAh g^–1^ (areal capacity: 1.66 mAh cm^–2^) after 100 cycles, corresponding to an 89.2% retention (Fig. 6e, f). Compared with previously reported Si-based full-cells, the Si/a-Sn/CoSi_2_/G/C|NCM811 full-cell exhibited a competitive cycle life, capacity retention, and rate performance (Fig. S33 and Table S6) [17, 19, 37, 41–51]. As a practical demonstration, the full-cell successfully powered three commercial green LED bulbs (3 V) (Fig. S34).

The Si/a-Sn/CoSi_2_/G/C nanocomposite anode was further evaluated in a sulfide-based ASSLB employing an argyrodite LPSC SE with high ionic conductivity (Fig. S35). Prior to electrochemical evaluation, the structural stability of the Si/a-Sn/CoSi_2_/G/C nanocomposite under the high pressing condition used for ASSLB assembly (375 MPa) was examined by TEM analyses (Fig. S36). The results confirm that the nanoscale morphology and phase distribution are well preserved without noticeable structural degradation, indicating that the applied pressure primarily promotes electrode densification and improved interparticle contact. In an ASSLB half-cell configuration (Fig. S37), the Si/a-Sn/CoSi_2_/G/C anode delivered initial discharge/charge capacities of 1501.7 and 1137.0 mAh g^–1^, respectively, corresponding to an ICE of 75.7% (Fig. S38), comparable to those obtained in liquid electrolyte LIBs. Although carbon incorporation slightly reduced the ICE compared with the carbon-free Si/a-Sn/CoSi_2_ anode, which delivered initial discharge/charge capacities of 2127.8 and 1718.6 mAh g^–1^ with an ICE of 80.8%, it markedly improved the cycling stability. These results highlight the essential role of carbon in stabilizing the electrode structure and electrode–electrolyte interface in sulfide-based ASSLBs. In many alloy-type ASSLB anodes, the incorporation of a large fraction of SE to ensure sufficient Li^+^ transport inevitably reduces the overall energy density [30, 52–56]. In contrast, the Si/a-Sn/CoSi_2_/G/C anode can operate effectively without SE incorporation in the anode layer, owing to the enhanced Li^+^ transport kinetics enabled by the continuous percolation network established by the multifunctional matrices. This SE-free configuration maximizes the active material fraction while retaining compatibility with conventional slurry-casting processes, thereby offering clear advantages in terms of both practical energy density and manufacturing scalability. This feature represents a significant advantage for the practical implementation of alloy-type anodes in ASSLBs.

### Practical Sulfide-Based ASSLB Full-Cell Performance and a Stabilized LPSC Interphase

**Fig. 7:**
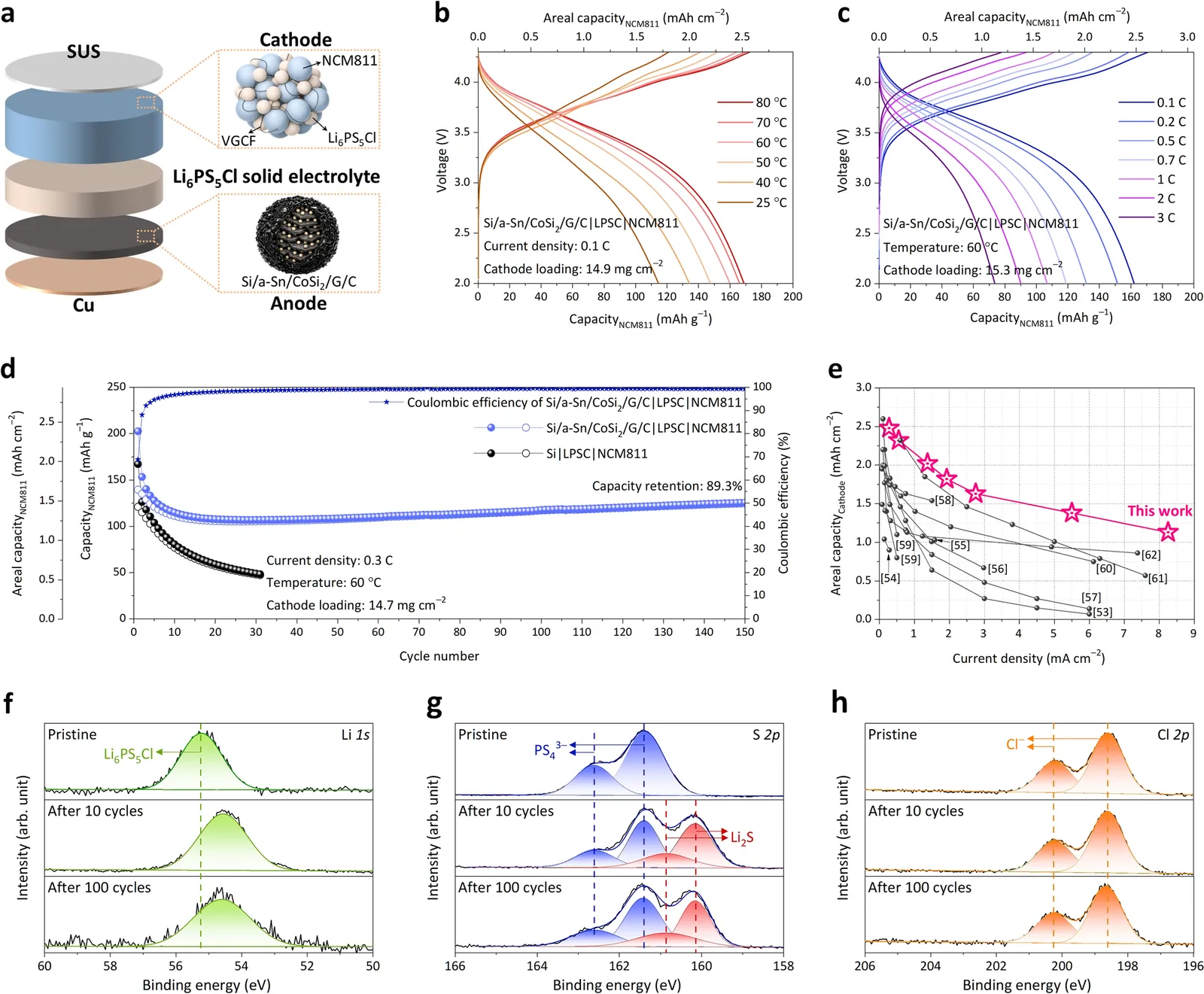
Electrochemical performance of the Si/a-Sn/CoSi_2_/G/C|LPSC|NCM811 sulfide-based ASSLB full-cell. **a** Schematic illustration of the full-cell configuration comprising a Si/a-Sn/CoSi_2_/G/C anode, an LPSC SE, and an NCM811 composite cathode. **b** Voltage profiles tested at 0.1 C over 25–80 °C (cathode loading: 14.9 mg cm^–2^). **c** Voltage profiles at current rates from 0.1 to 3 C (cathode loading: 15.3 mg cm^–2^). **d** Cycling performance comparison between Si/a-Sn/CoSi_2_/G/C|LPSC|NCM811 and Si|LPSC|NCM811 full-cells (cathode loading: 14.7 mg cm^–2^). **e** Comparison of the electrochemical performance of the Si/a-Sn/CoSi_2_/G/C|LPSC|NCM811 full-cell with those of previously reported Si-based ASSLB full-cells. **f** XPS spectra of Li *1s* region, **g** S *2p* region, and **h** Cl *2p* region for LPSC SE before cycling, after 10 cycles, and after 100 cycles

Sulfide-based ASSLB full-cells were assembled using the Si/a-Sn/CoSi_2_/G/C anode, LPSC SE, and an NCM811 composite cathode (Fig. 7a). With a cathode loading of 14.9 mg cm^–2^, the cells delivered reversible discharge capacities of 114.5–168.8 mAh g^–1^ (1.71–2.52 mAh cm^–2^) over a wide temperature range of 25–80 °C (Fig. 7b), demonstrating stable operation across a practical temperature range. Rate capability tests (cathode loading: 15.3 mg cm^–2^) produced results of 162.1 mAh g^–1^ (2.48 mAh cm^–2^) at 0.1 C and 73.6 mAh g^–1^ (1.13 mAh cm^–2^) at 3 C (Fig. 7c). Notably, no short-circuiting was observed even at 3 C, suggesting mechanically and electrochemically stable electrode–electrolyte interfaces and kinetically efficient solid-state operation. The specific energy density of the Si/a-Sn/CoSi_2_/G/C|LPSC|NCM811 ASSLB full-cell was estimated to be 301.2 Wh kg^–1^ at 0.1 C, assuming a commercially relevant LPSC thickness of 30 μm (Table S7), thereby indicating its potential for high-energy ASSLB configurations. Long-term cycling at 0.3 C (cathode loading: 14.7 mg cm^–2^) delivered an initial discharge capacity of 139.5 mAh g^–1^ (2.05 mAh cm^–2^) with 89.3% capacity retention after 150 cycles (Figs. 7d and S39a). In contrast to the Si|LPSC|NCM811 full-cell (Figs. 7d and S39b), the Si/a-Sn/CoSi_2_/G/C|LPSC|NCM811 cell exhibited markedly improved retention and enhanced structural stability. Moreover, the ASSLB full-cell with the Si/a-Sn/CoSi_2_/G/C anode delivered higher areal capacities across a wide current density range compared with previously reported Si-based ASSLB systems (Fig. 7e and Table S8) [53–62].

XPS analysis was performed to evaluate the interfacial stability between the LPSC SE and the Si/a-Sn/CoSi_2_/G/C anode before and after cycling (Fig. 7f–h). The Li *1s* peak of the pristine SE appears at 55.3 eV and shifts to 54.6 eV after 10 cycles, consistent with the formation of reduced Li-containing interphase species during the initial cycles. Notably, no further shift is observed after 100 cycles, indicating that the interphase becomes stabilized after the initial activation stage (Fig. 7f). In the S *2p* spectra (Fig. 7g), the LPSC-related peaks at 161.4 and 162.6 eV, assigned to PS_4_^3–^ thiophosphate units, are retained after cycling, while new peaks at 160.2 and 160.9 eV, assigned to Li_2_S, remain nearly unchanged up to 100 cycles, further supporting interphase stabilization. In contrast, the Cl *2p* spectra show no discernible evolution, which is likely related to the relatively low Cl contribution in LPSC. These results indicate that, although limited reductive decomposition of LPSC occurs during the initial cycles, the reactions are self-limiting and lead to the formation of a stable passivating interphase, thereby ensuring interfacial compatibility and preventing continuous degradation during cycling.

### Morphological Evolution in ASSLBs: Failure of Pristine Si Versus Stable Dense Architecture of Si/a-Sn/CoSi_2_/G/C

**Fig. 8:**
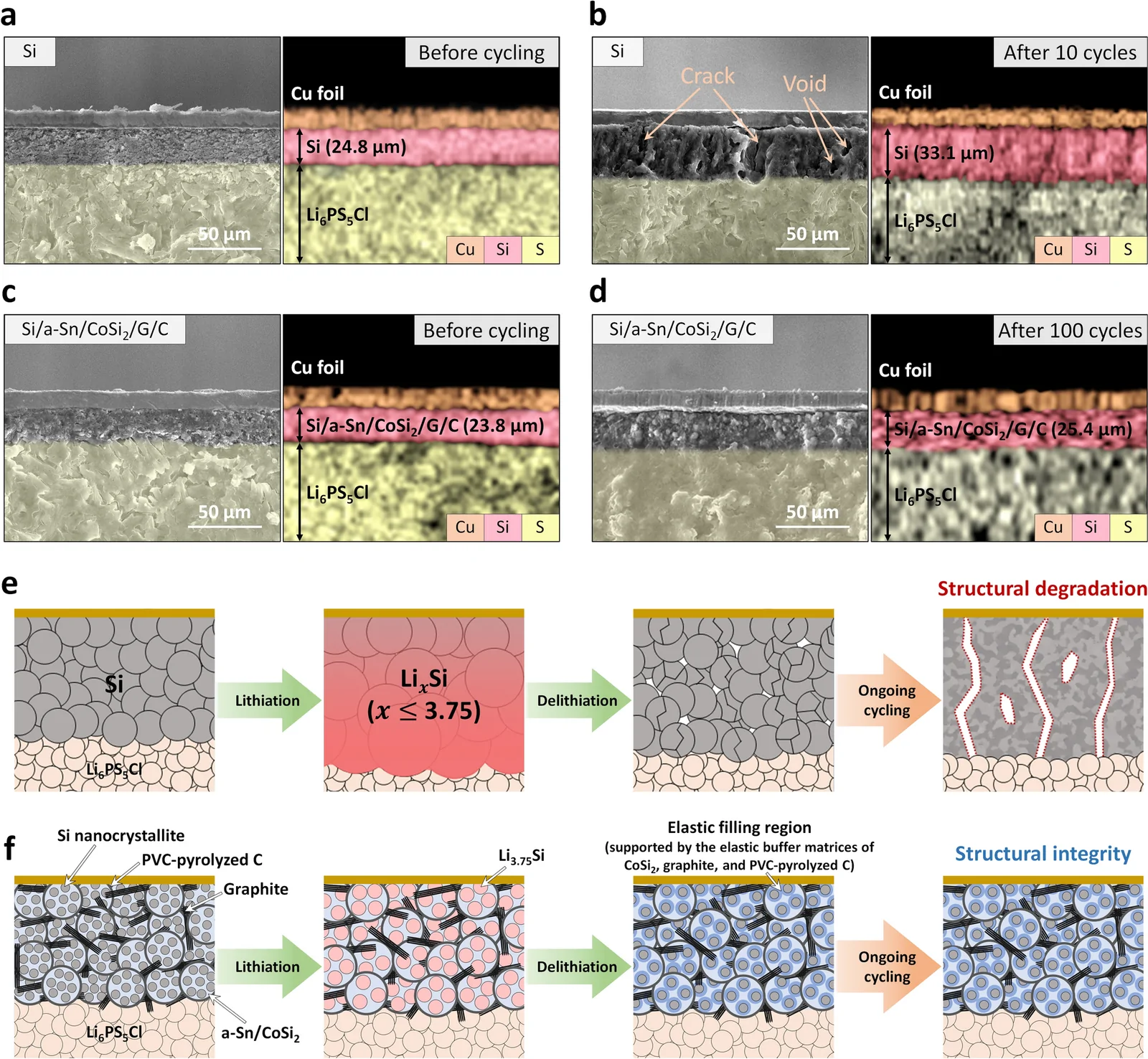
Morphological evolution of Si and Si/a-Sn/CoSi_2_/G/C anodes in sulfide-based ASSLBs. Cross-sectional SEM images with corresponding EDX elemental mapping of the pristine Si anode **a** before cycling and **b** after 10 cycles. Cross-sectional SEM images with corresponding EDX elemental mapping of the Si/a-Sn/CoSi_2_/G/C anode **c** before cycling and **d** after 100 cycles. Schematic illustrations demonstrating the structural evolution of the **e** Si and **f** Si/a-Sn/CoSi_2_/G/C anodes during cycling

The morphological evolution of the pristine Si and Si/a-Sn/CoSi_2_/G/C anodes in ASSLB configurations was comparatively investigated using cross-sectional SEM. As shown in Fig. 8a, the pristine Si anode initially formed intimate contact with the LPSC SE. However, after only 10 cycles, severe mechanically driven degradation occurred, as evidenced by extensive vertical cracking and internal void formation (Fig. 8b). In parallel, the electrode thickness increased from 24.8 to 33.1 μm (33.5% expansion), reflecting the large and poorly accommodated volume changes in the Si during repeated lithiation/delithiation and explaining its rapid capacity decay and premature mechanical failure. In contrast, the Si/a-Sn/CoSi_2_/G/C anode exhibited excellent dimensional and morphological stability. Its thickness increased only slightly from 23.8 μm (Figs. 8c and S40) to 25.4 μm after 100 cycles (Figs. 8d and S41), corresponding to a minimal expansion of 6.7%. Importantly, the electrode retained a dense, well-connected architecture without pronounced cracking, indicating sustained interparticle/interfacial contact with the SE throughout cycling. The initial compaction densities of the pristine Si and Si/a-Sn/CoSi_2_/G/C anodes were estimated to be 0.770 and 0.803 g cm^–3^, respectively, based on the electrode mass, thickness, and area, indicating comparable densification levels. Accordingly, the distinct expansion behaviors are mainly attributed to the intrinsic structural characteristics of the anode materials rather than differences in electrode packing density. The distinct structural evolution is summarized schematically in Fig. 8e, f. For the pristine Si, limited and heterogeneous ion/electron transport in the solid-state environment promoted nonuniform reactions within the electrode, leading to stress concentration, irreversible fracture, and void formation (Fig. 8e). In contrast, the Si/a-Sn/CoSi_2_/G/C anode enabled more homogeneous electrochemical reactions within the Si nanocrystallites and maintained a dense, well-connected structure during cycling (Fig. 8f). This morphological stability originated from the integrated multifunctional architecture composed of well-deformable, electronically conductive amorphous Sn, a mechanically robust and elastic CoSi_2_ framework, a highly Li-reversible, stress-mitigating graphite scaffold, and a highly elastic, electronically conductive PVC-pyrolyzed amorphous carbon shell. Together, these components sustained continuous electronic percolation and favorable Li⁺ transport kinetics while mitigating stress-driven degradation, thereby enabling the superior long-term cycling performance of the Si/a-Sn/CoSi_2_/G/C anode in the sulfide-based ASSLBs. Despite these promising advances, several challenges remain for the practical application of Si-based anodes. In particular, achieving high areal capacity with stable cycling while optimizing the balance among structural stability, conductivity, and active material fraction remains a key issue [63–65]. In addition, reducing the stack pressure requirement in ASSLBs is critical for practical implementation. In this regard, the rational design of multifunctional and scalable composite architectures that synergistically optimize mechanical, electrical, and interfacial properties will be a key direction for next-generation high-energy batteries.

## Conclusions

In this study, a Si/a-Sn/CoSi_2_/G/C nanocomposite incorporating multifunctional conductive–elastic matrices was developed using a simple and scalable fabrication route. The hierarchical architecture, which comprised well-deformable, electronically conductive amorphous Sn, a mechanically robust and elastic CoSi_2_ framework, a highly Li-reversible, stress-mitigating graphite scaffold, and a highly elastic, electronically conductive PVC-pyrolyzed amorphous carbon shell, established continuous electronic percolation, favorable Li⁺ transport kinetics, and multilevel stress accommodation with elastic recovery, thereby overcoming the intrinsic chemo-mechanical limitations of Si anodes. As a result, the Si/a-Sn/CoSi_2_/G/C anode demonstrated outstanding electrochemical performances in both LIB and ASSLB systems. In LIB full-cells paired with an NCM811 cathode, it achieved a high energy density of 434.4 Wh kg⁻^1^ with durable cycling stability and an excellent rate capability. In a sulfide-based ASSLB, the Si/a-Sn/CoSi_2_/G/C|LPSC|NCM811 full-cell delivered an energy density exceeding 300 Wh kg⁻^1^ while maintaining a dense electrode morphology and stable interfacial contact with the solid electrolyte (LPSC). Overall, this study demonstrated that integrating multifunctional conductive–elastic buffering matrices provides a practical and scalable design strategy for stabilizing Si anodes, paving the way for high-energy next-generation LIBs and ASSLBs.

## Supplementary Information

Below is the link to the electronic supplementary material.

Supplementary file1 (DOCX 40191 kb)
