# Supplementary material for: Multifunctional Conductive and Elastic Matrices-Engineered Si Nanocomposite Anodes for Liquid and Solid-State Lithium Batteries
Source: Nanomicro Lett. 2026 Jun 22;18:411. doi: 10.1007/s40820-026-02258-w (PMC13287344; doi:10.1007/s40820-026-02258-w)
Supplement: Supplementary file 1 — Supplementary file1 (DOCX 40191 kb) [file 40820_2026_2258_MOESM1_ESM.docx]

Supporting Information for

**Multifunctional Conductive and Elastic Matrices–Engineered Si Nanocomposite Anodes for Liquid and Solid-State Lithium Batteries**

Young-Han Lee^1,2^, Je-Hyeon Han^1,2^, Deok-Gyu Kim^1,2^, Jung-Woon Yoo^1,2^, Yoon-Cheol Ha^3,4^*, Jae-Hun Kim^5^*, and Cheol-Min Park^1,2^*

^1^ Department of Advanced Materials Science and Engineering, Kumoh National Institute of Technology, Gumi, Gyeongbuk 39177, Republic of Korea

^2^ Department of Energy Engineering Convergence, Kumoh National Institute of Technology, Gumi, Gyeongbuk 39177, Republic of Korea

^3^ Battery Research Division, Korea Electrotechnology Research Institute (KERI), Changwon, Gyeongnam 51543, Republic of Korea

^4^ Electric Energy Materials Engineering, KERI School, University of Science and Technology (UST), Daejeon 34113, Republic of Korea

^5^ School of Materials Science and Engineering, Kookmin University, Seoul 02707, Republic of Korea

*Corresponding authors. E-mail: cmpark@kumoh.ac.kr (Cheol-Min Park)； jaehunkim@kookmin.ac.kr (Jae-Hun Kim)；ycha@keri.re.kr (Yoon-Cheol Ha)

**Supplementary Figures and Tables**


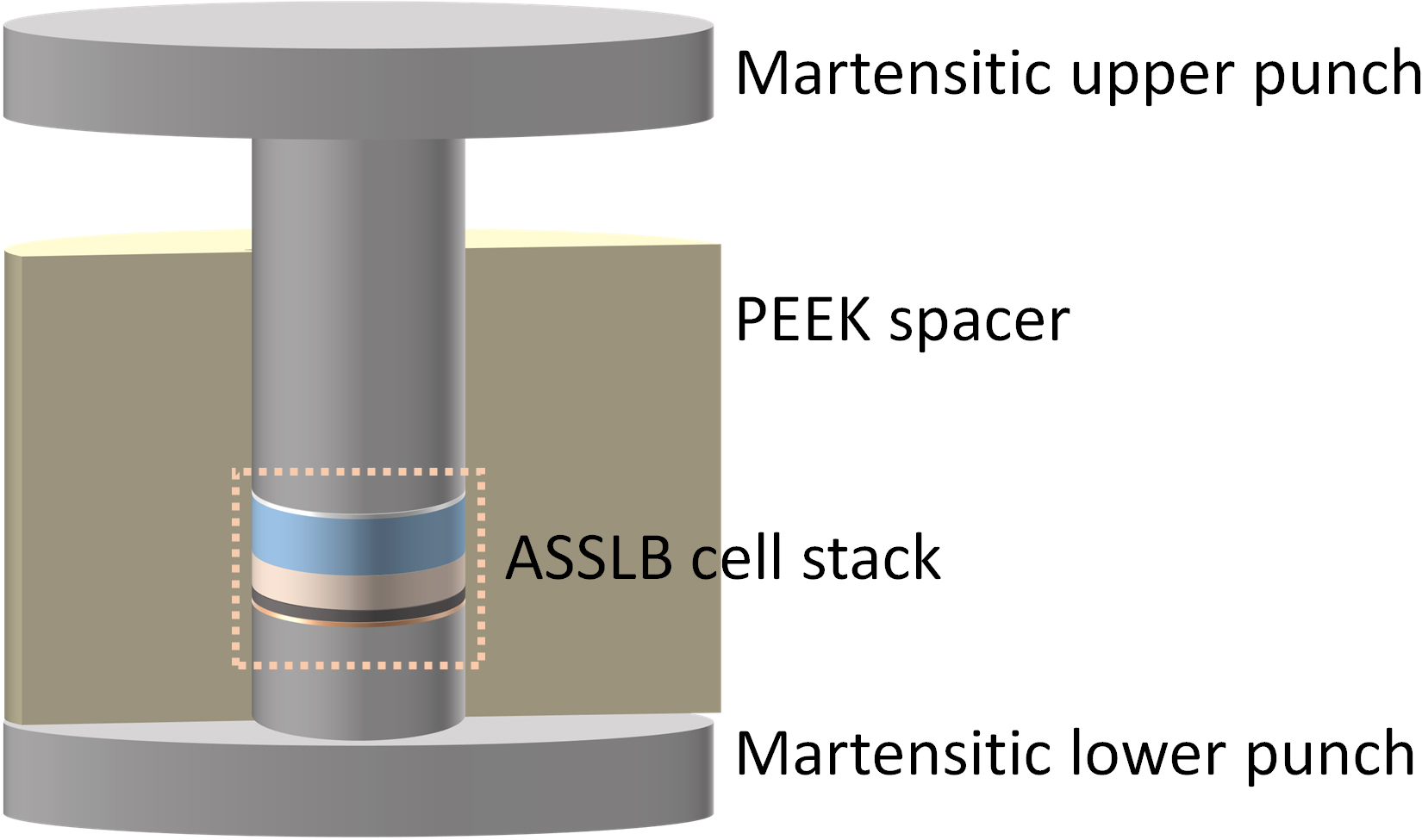


**Fig. S1** Schematic illustration of the ASSLB cell configuration used for electrochemical testing


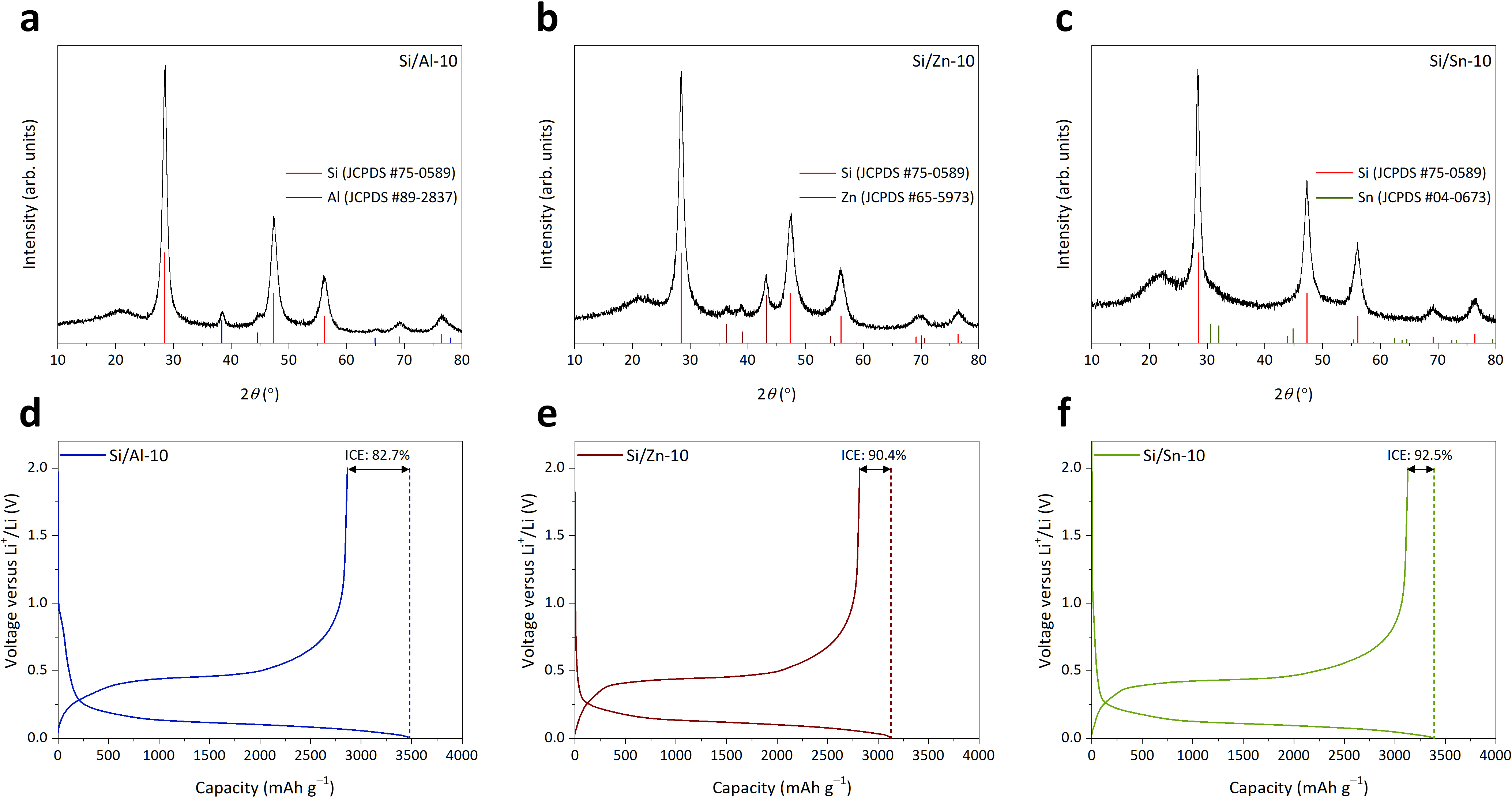


**Fig. S2** Structural and electrochemical comparison of Si/M (M = Al, Zn, Sn) composites prepared under identical high-power MM conditions with 10 wt% metal incorporation. XRD patterns of **a** Si/Al-10, **b** Si/Zn-10, and **c** Si/Sn-10 composites. Voltage profiles of **d** Si/Al-10, **e** Si/Zn-10, and **f** Si/Sn-10 composites during the first cycle


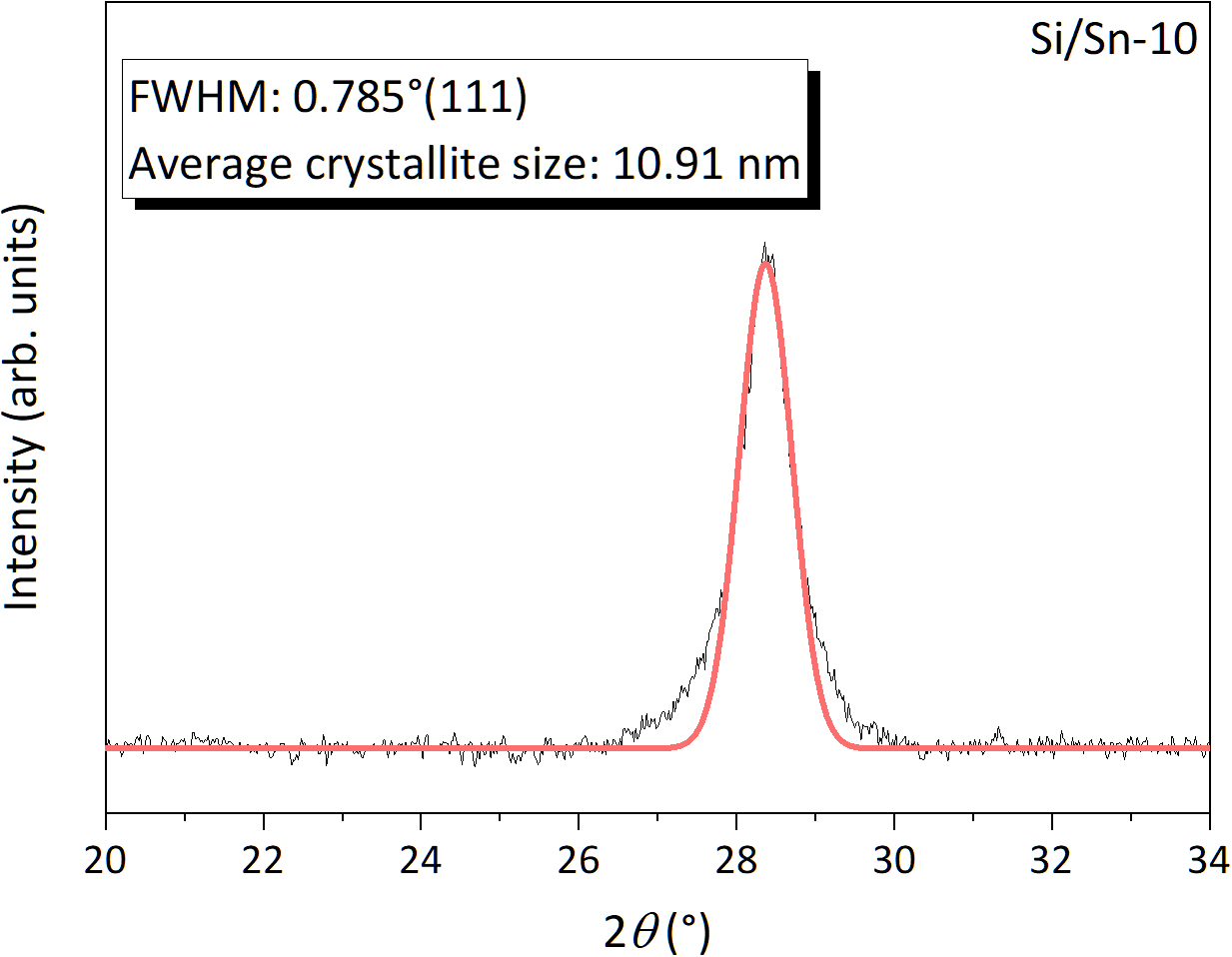


**Fig. S3** Average size of Si crystallites in Si/Sn-10 composites calculated using the Scherrer equation


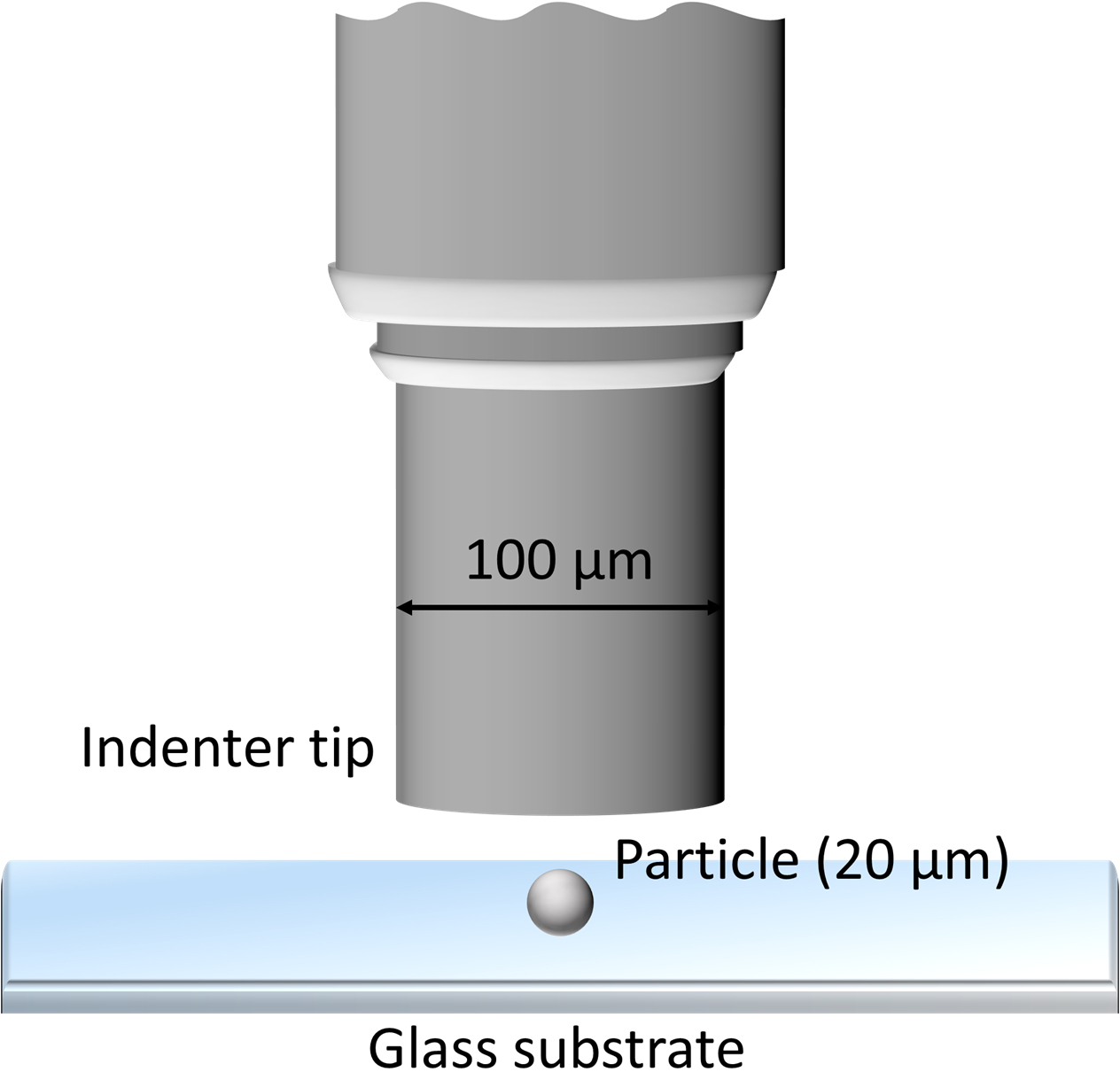


**Fig. S4** Schematic illustration of the nanoindentation test setup


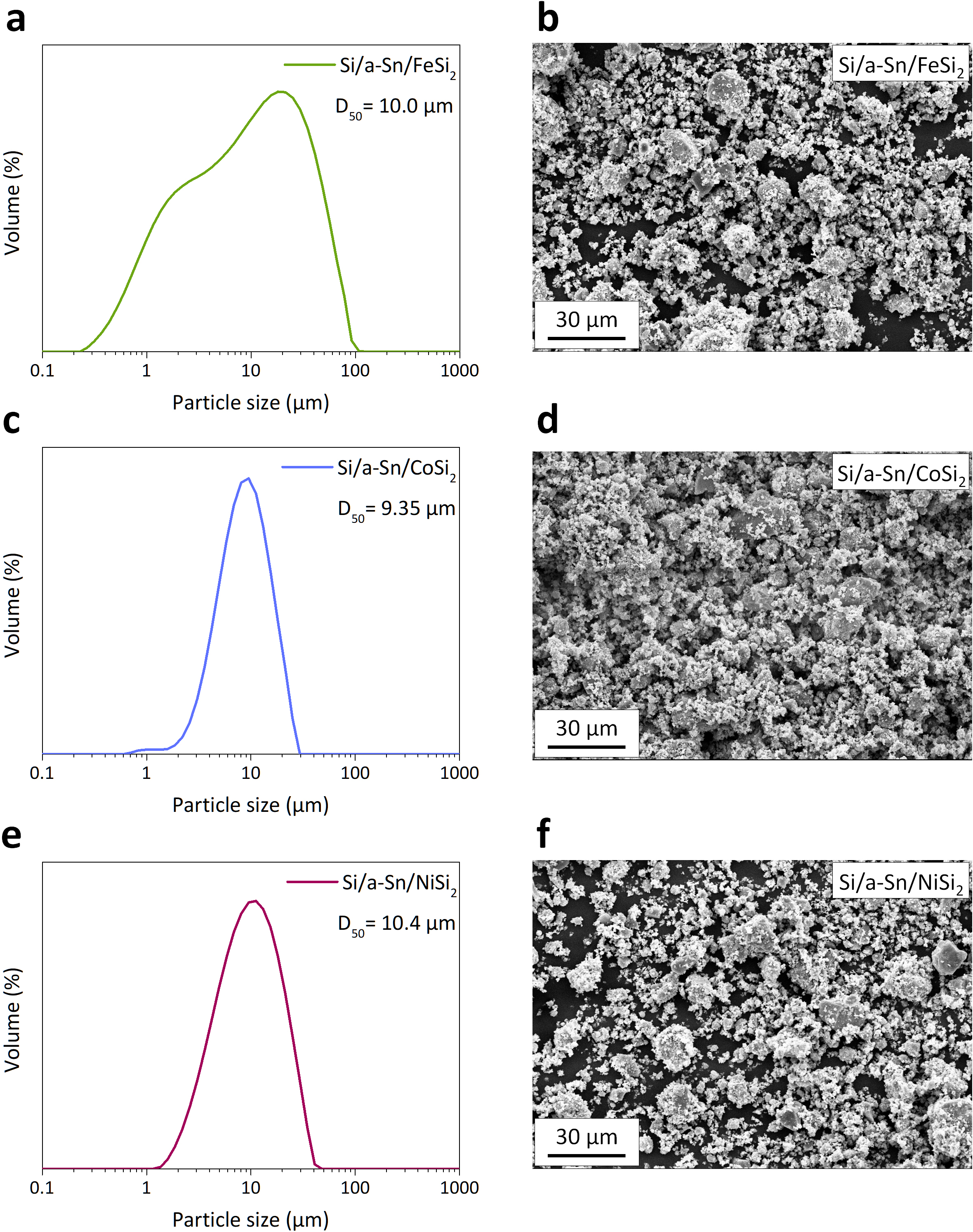


**Fig. S5** Morphological characteristics of the Si/a-Sn/TMS composites. PSA results for **a** Si/a-Sn/FeSi_2_, **c** Si/a-Sn/CoSi_2_, and **e** Si/a-Sn/NiSi_2_. SEM images of **b** Si/a-Sn/FeSi_2_, **d** Si/a-Sn/CoSi_2_, and **f** Si/a-Sn/NiSi_2_


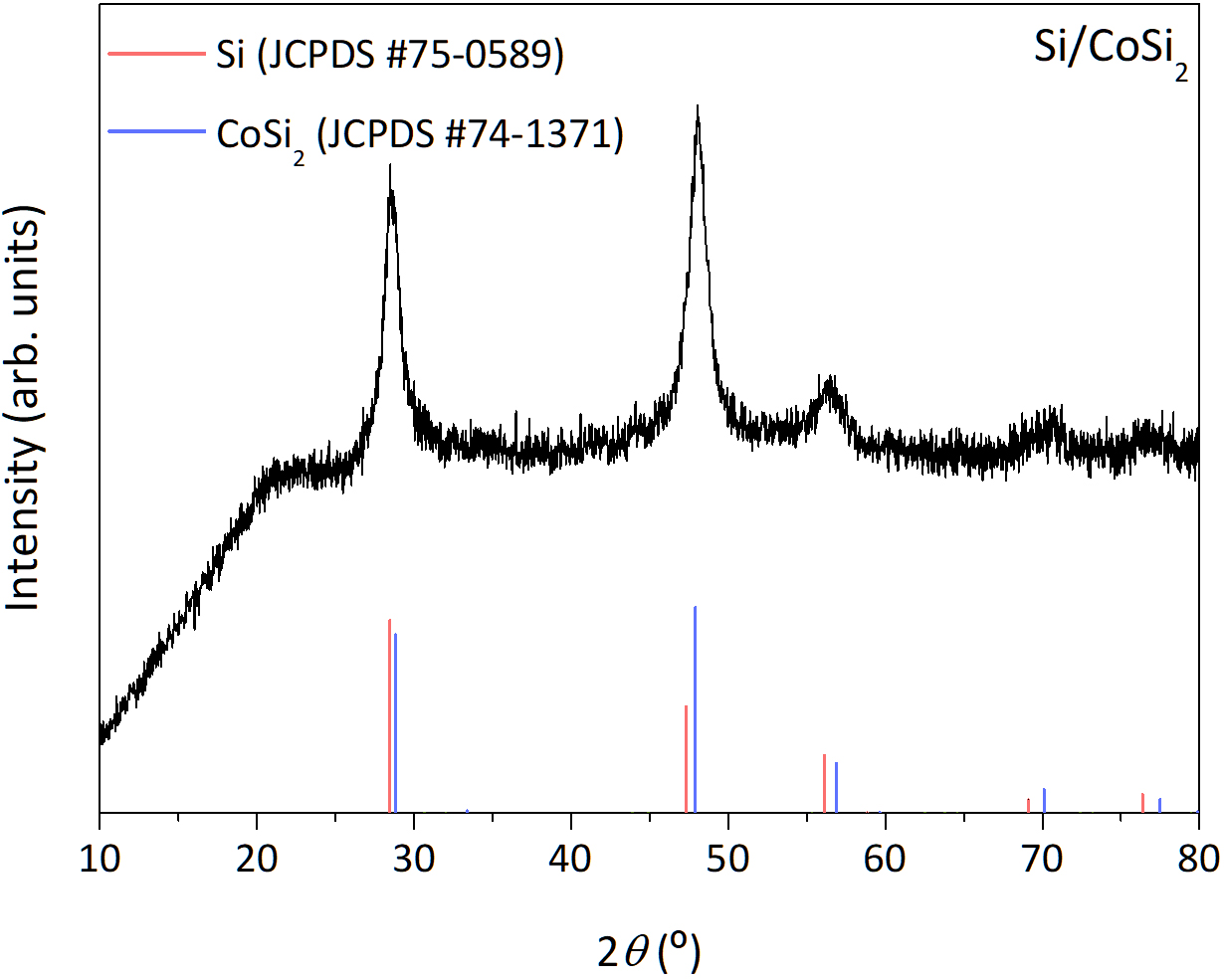


**Fig. S6** XRD pattern of the Si/CoSi_2_ composite synthesized *via* high-power MM


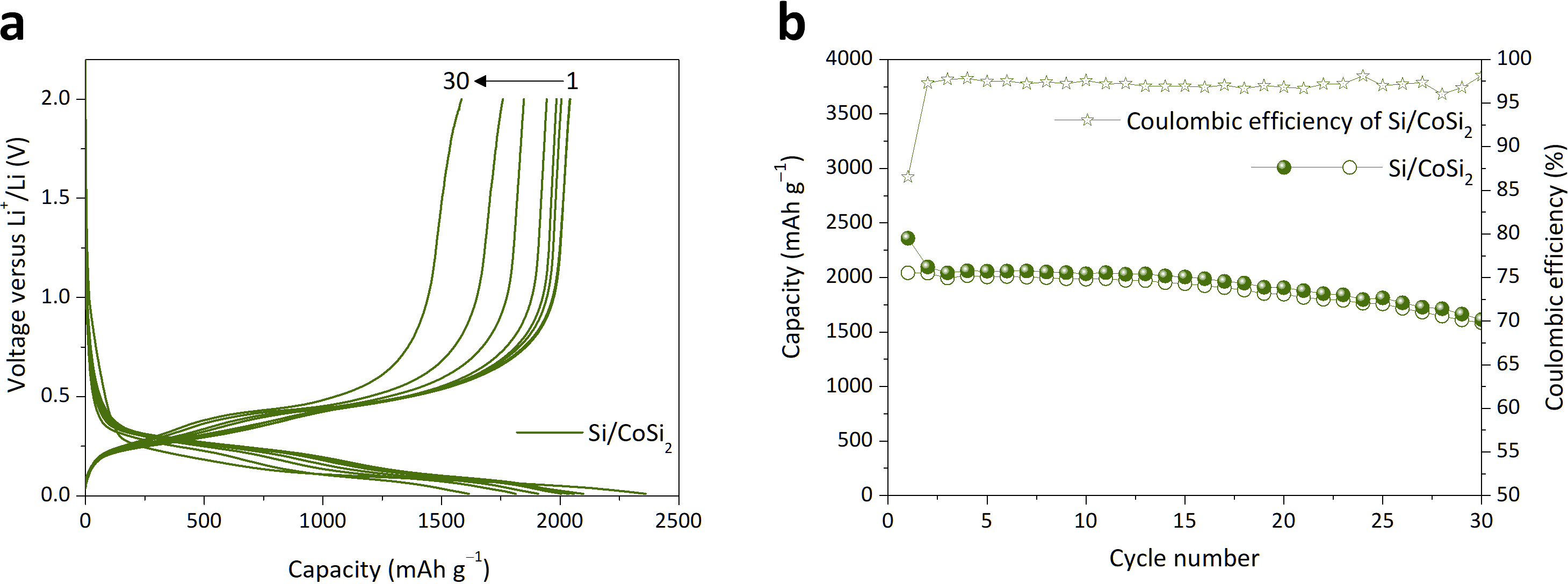


**Fig. S7** Electrochemical performance results for the Si/CoSi_2_ composite. **a** Galvanostatic voltage profiles of the Si/CoSi_2_ composite anode. **b** Long-term cycling performance of the Si/CoSi_2_ composite anode (current density: 300 mA g^–1^)


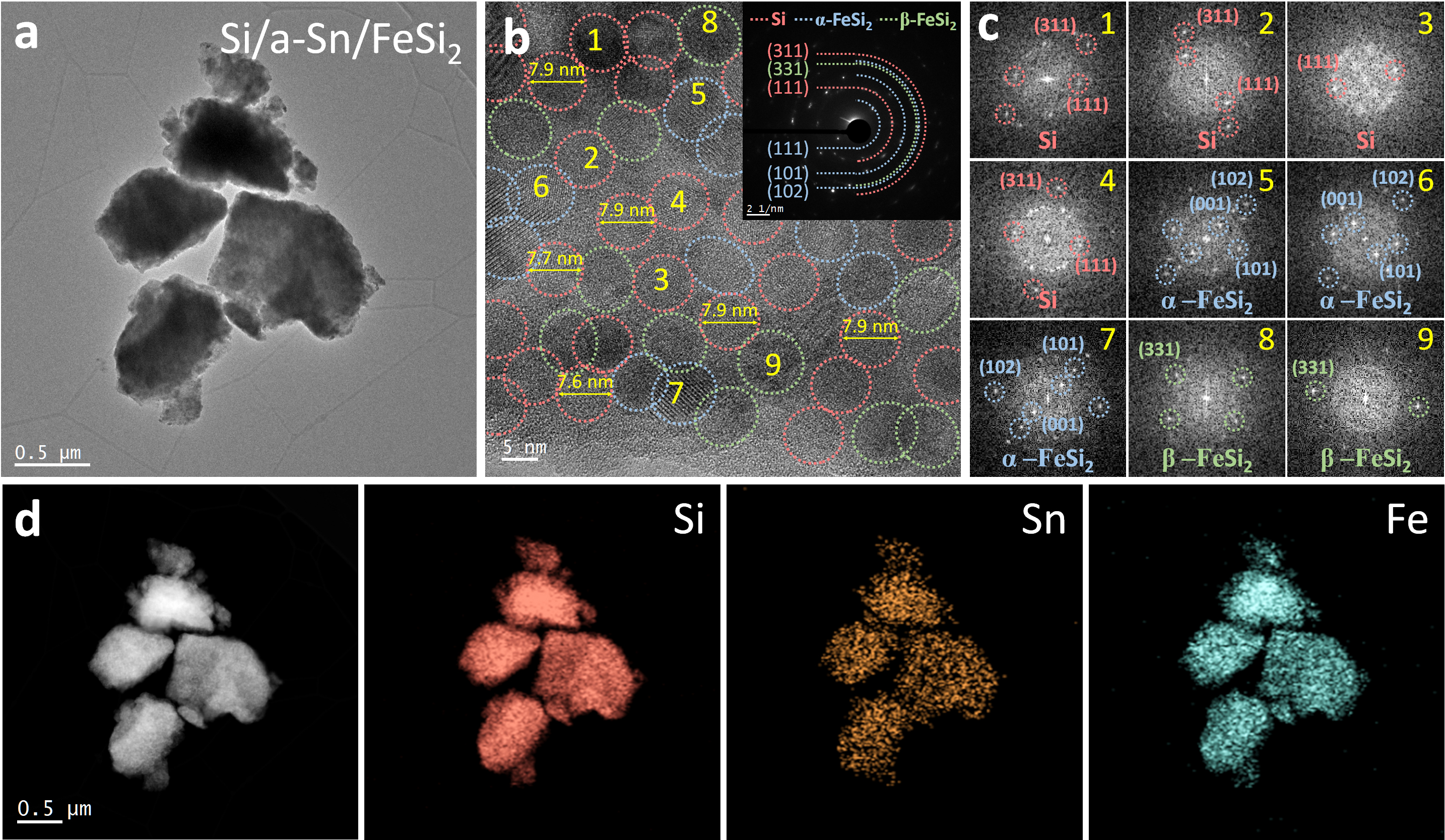


**Fig. S8** Microstructural characteristics of the Si/a-Sn/FeSi_2_ composite: **a** BFTEM image, **b** HRTEM image with SAED pattern, **c** FFT patterns for selected regions, and **d** STEM image with EDX elemental mappings


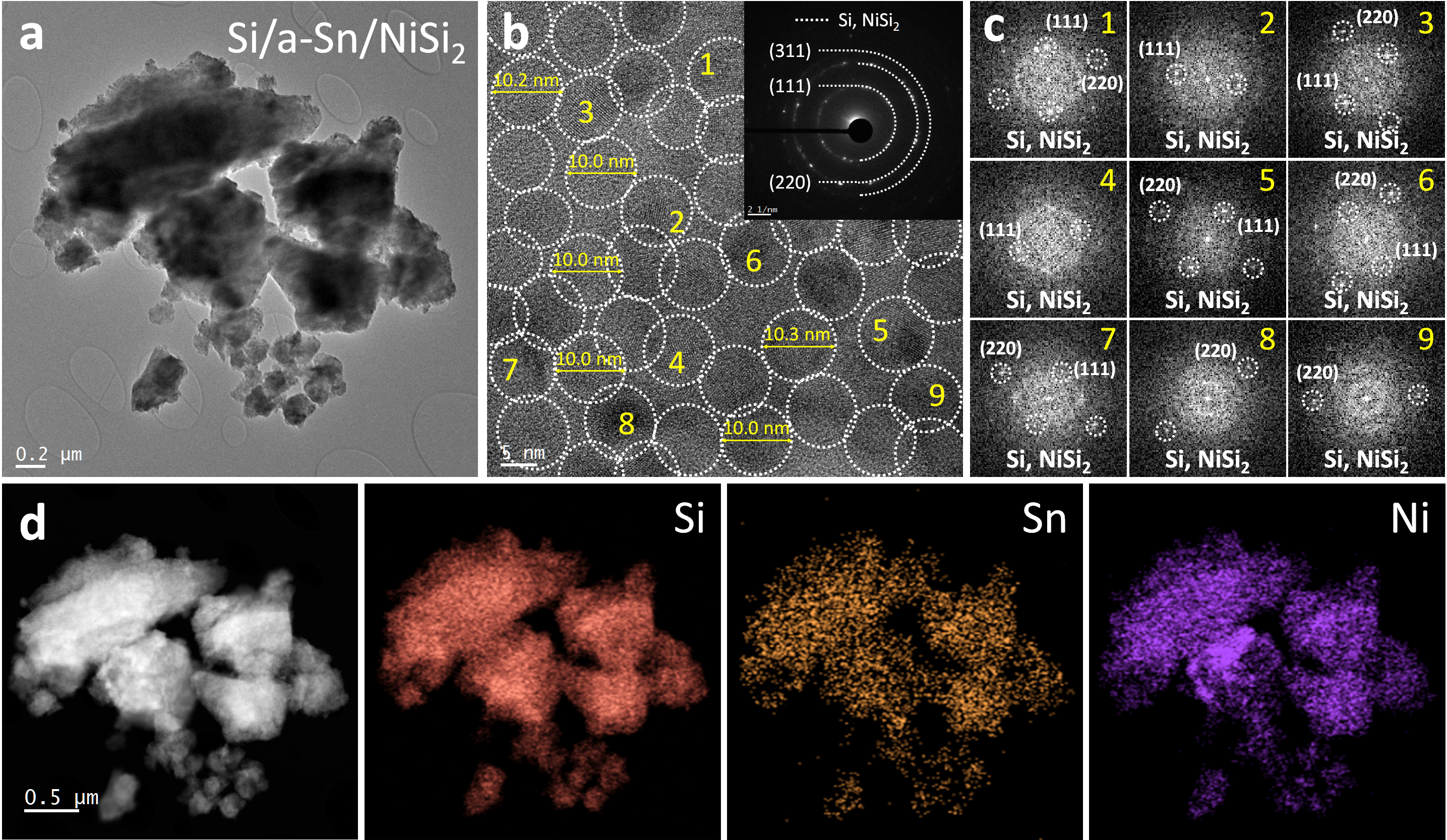


**Fig. S9** Microstructural characteristics of the Si/a-Sn/NiSi_2_ composite: **a** BFTEM image, **b** HRTEM image with SAED pattern, **c** FFT patterns for selected regions, and **d** STEM image with EDX elemental mappings


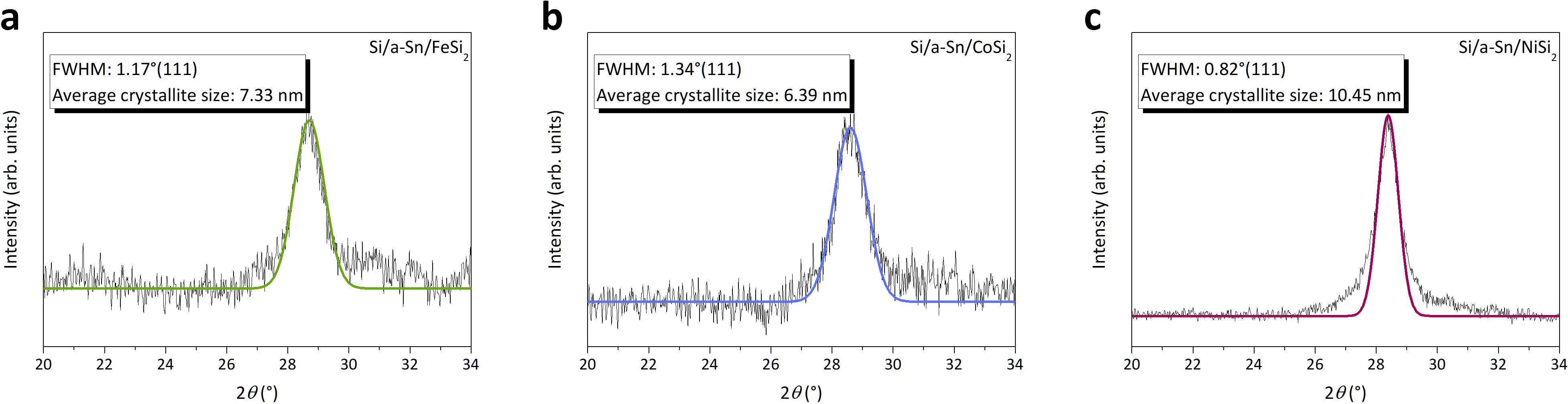


**Fig. S10** Average sizes of Si crystallites in Si/a-Sn/TMS composites calculated using the Scherrer equation: **a** Si/a-Sn/FeSi_2_, **b** Si/a-Sn/CoSi_2_, and **c** Si/a-Sn/NiSi_2_


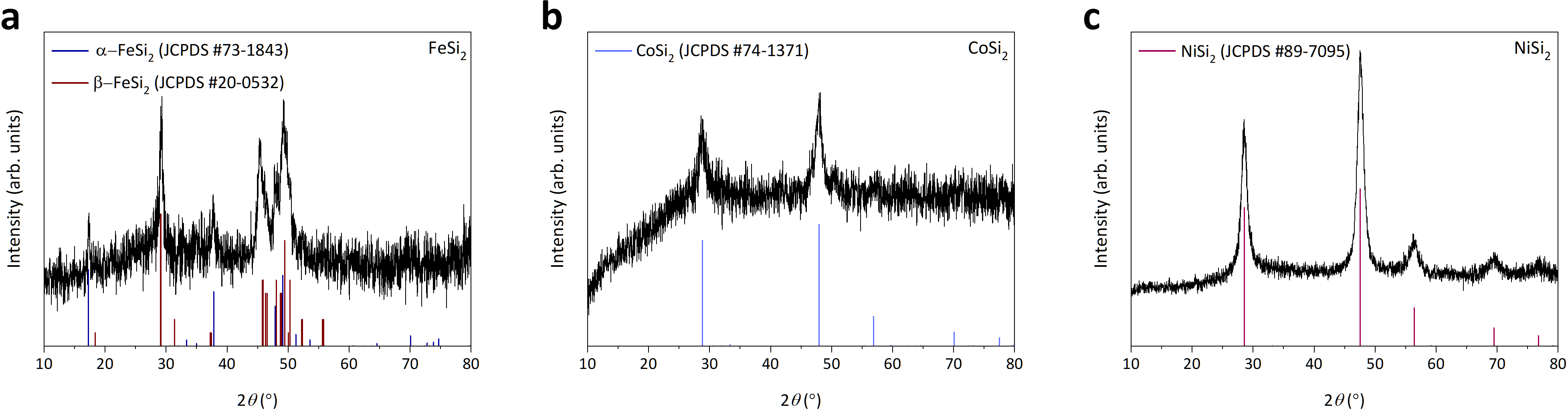


**Fig. S11** Structural characteristics of the high-power MM-derived TMS phases. XRD patterns of high-power MM-derived **a** FeSi_2_, **b** CoSi_2_, and **c** NiSi_2_


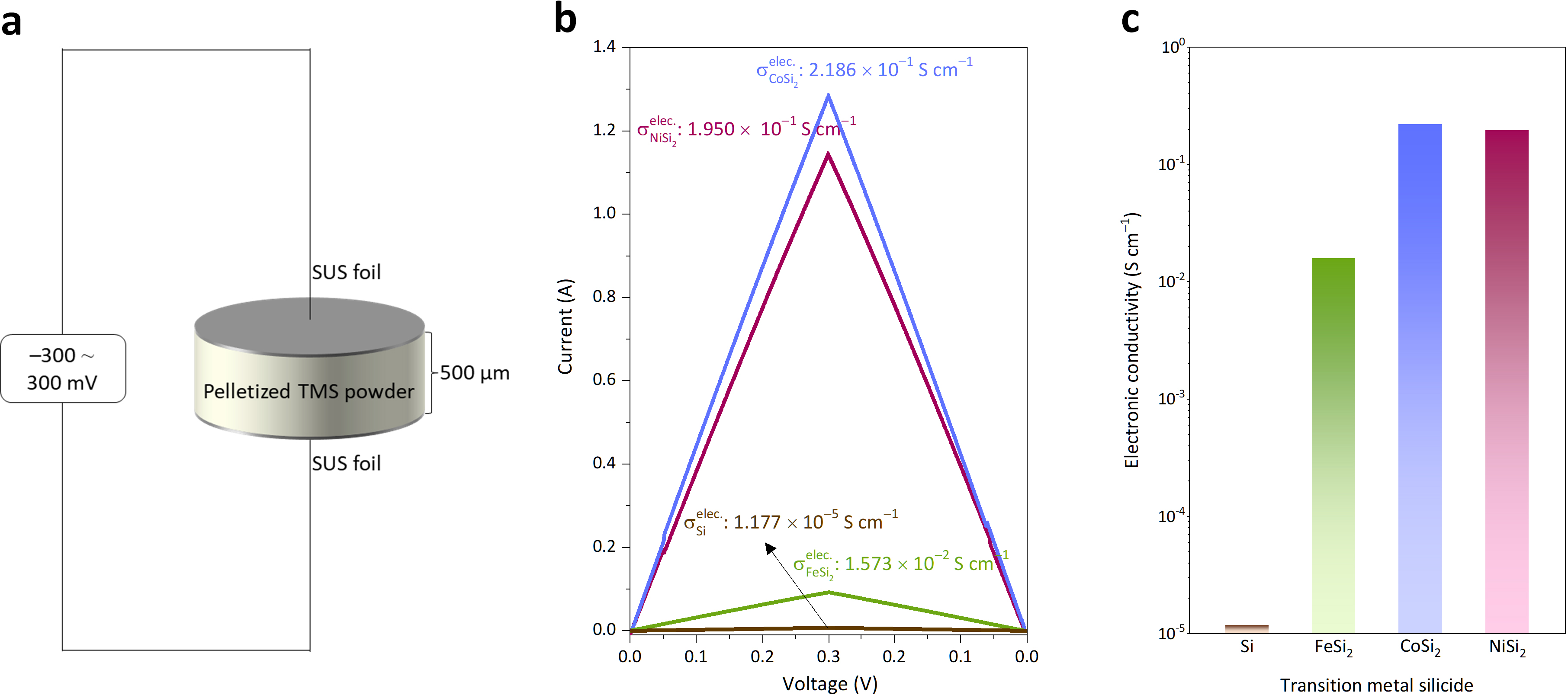


**Fig. S12** Electronic conductivity measurements of high-power MM-derived TMS phases (FeSi_2_, CoSi_2_, and NiSi_2_). **a** Schematic illustration of the SUS|TMS pellet|SUS symmetric cell configuration used to measure electronic conductivity. **b** I–V profiles of the TMS phases measured in the symmetric cell. **c** Electronic conductivities calculated from the slopes of the I–V curves


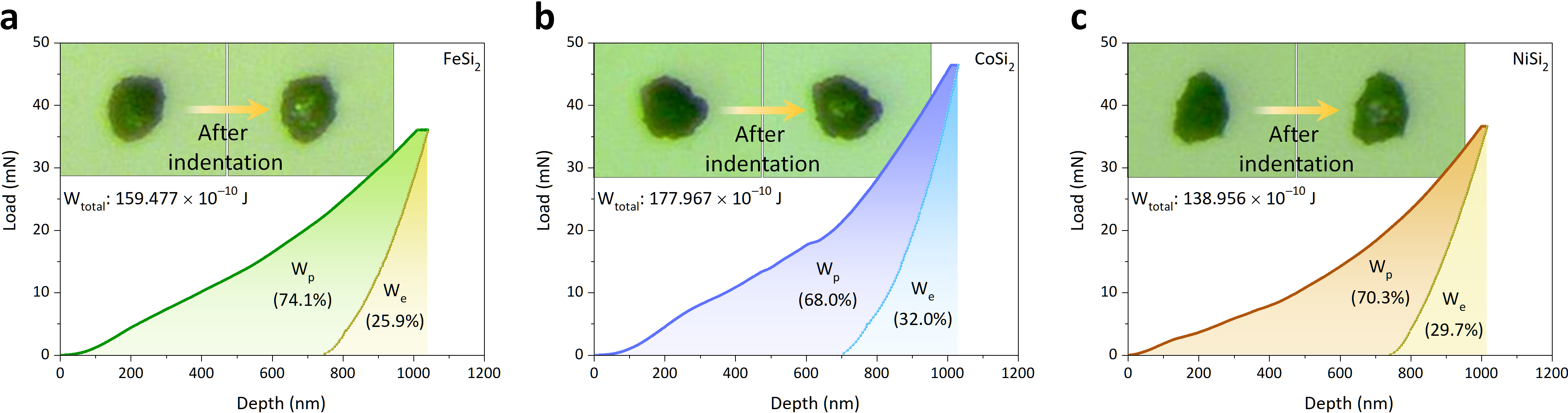


**Fig. S13** Load–displacement profiles obtained from nanoindentation tests on the high-power MM-derived TMS particles: **a** FeSi_2_, **b** CoSi_2_, and **c** NiSi_2_


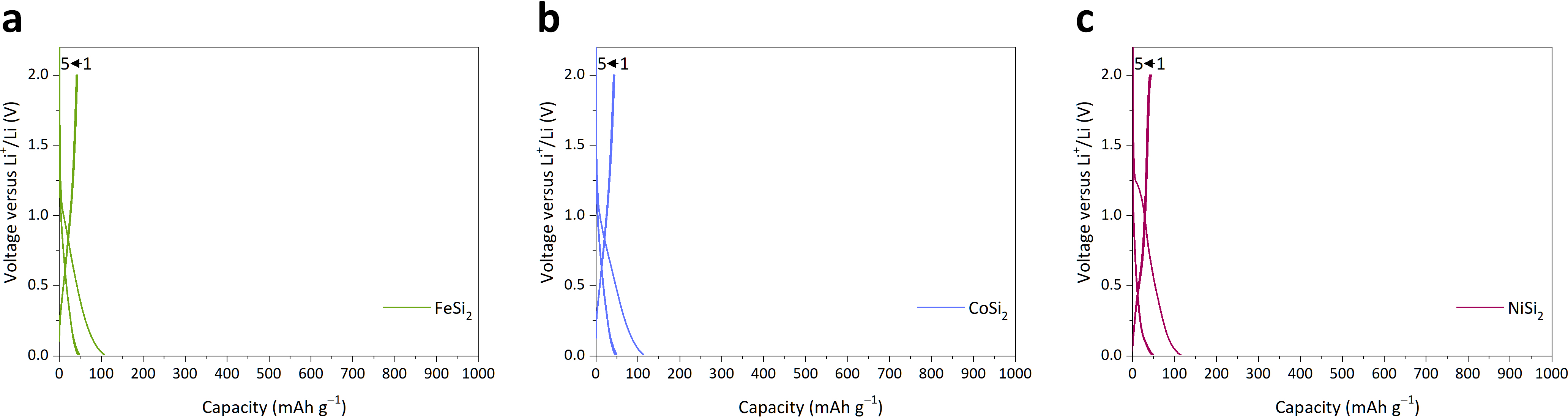


**Fig. S14** Voltage profiles of TMS anodes measured at a current density of 100 mA g^–1^. **a** FeSi_2_, **b** CoSi_2_, and **c** NiSi_2_


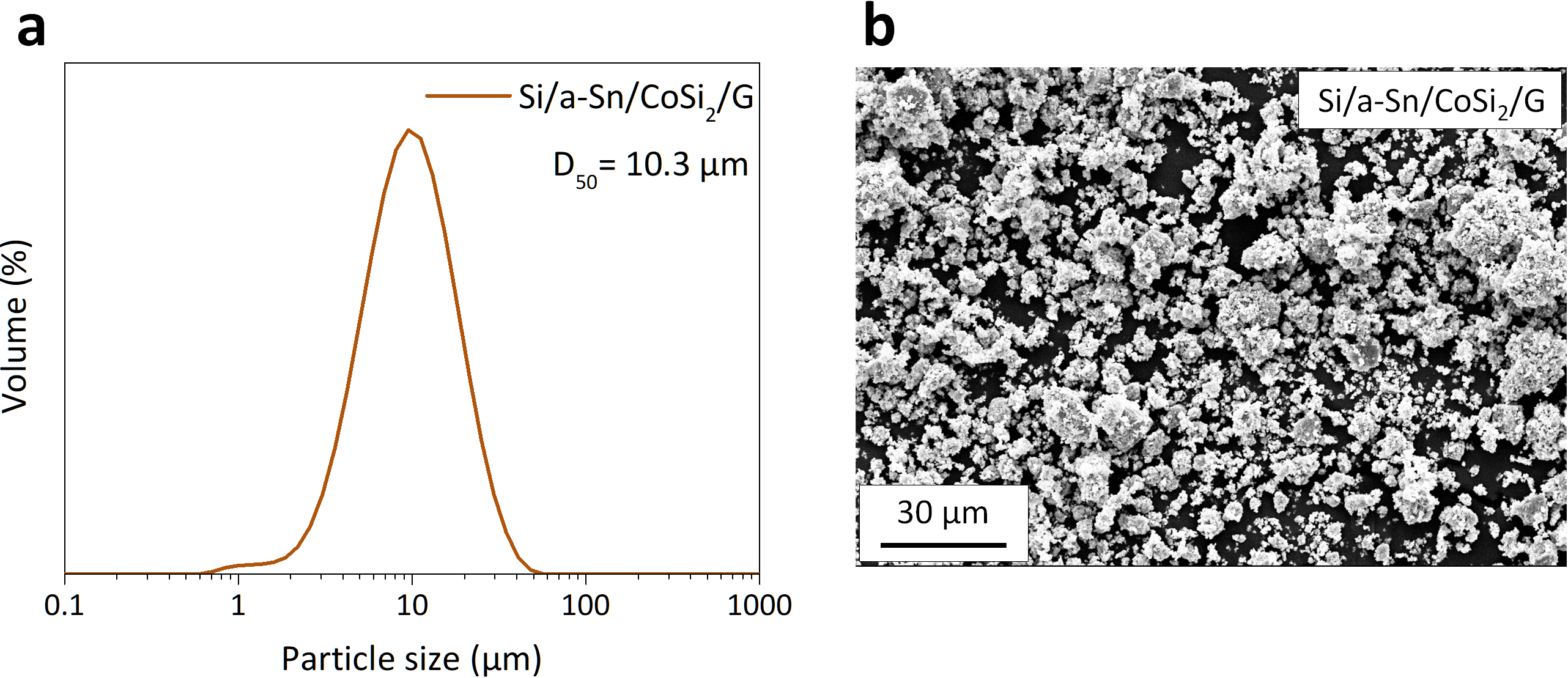


**Fig. S15** Morphological characteristics of the Si/a-Sn/CoSi_2_/G composite: **a** PSA result and **b** SEM image


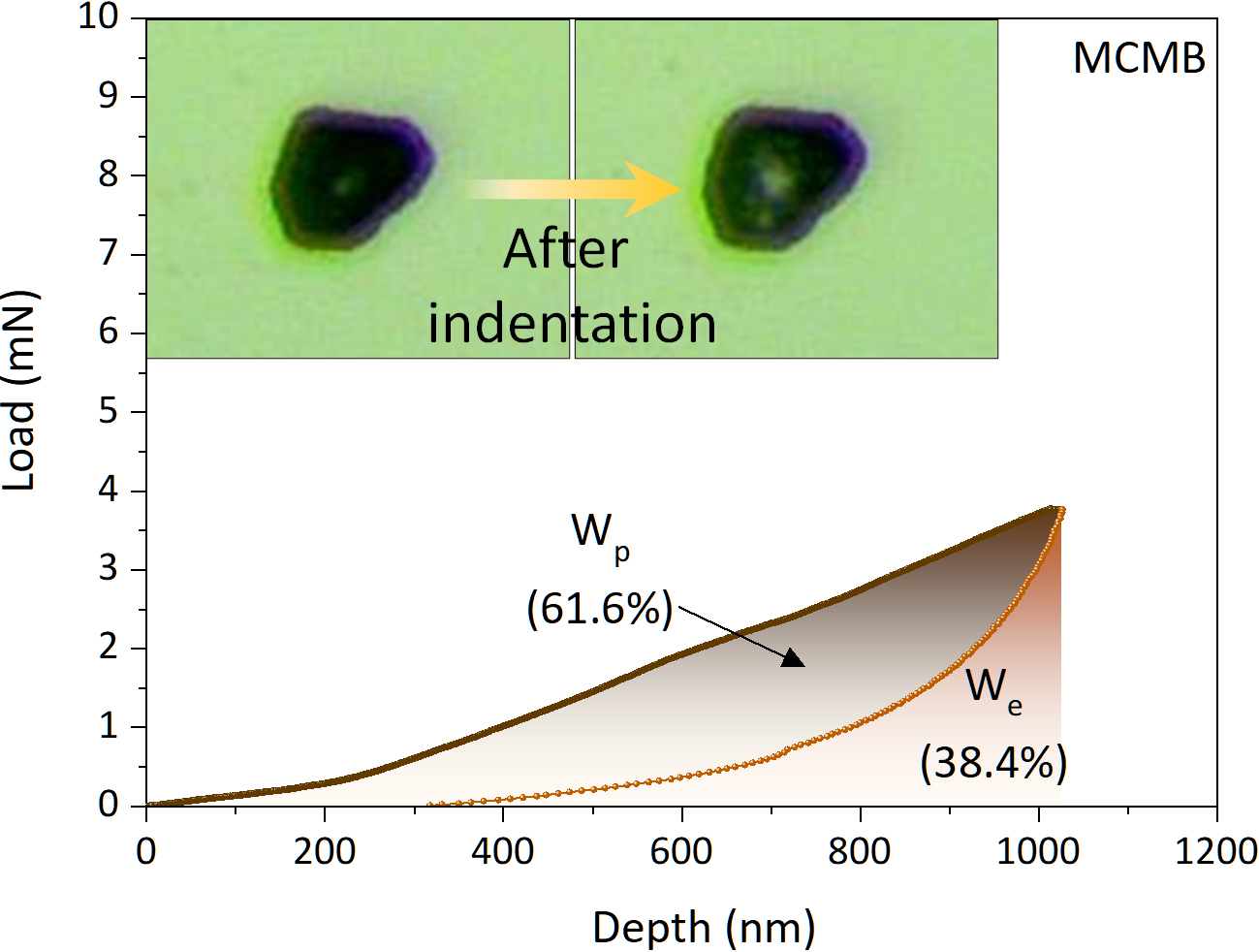


**Fig. S16** Load–displacement profile obtained from a nanoindentation test on an MCMB graphite particle


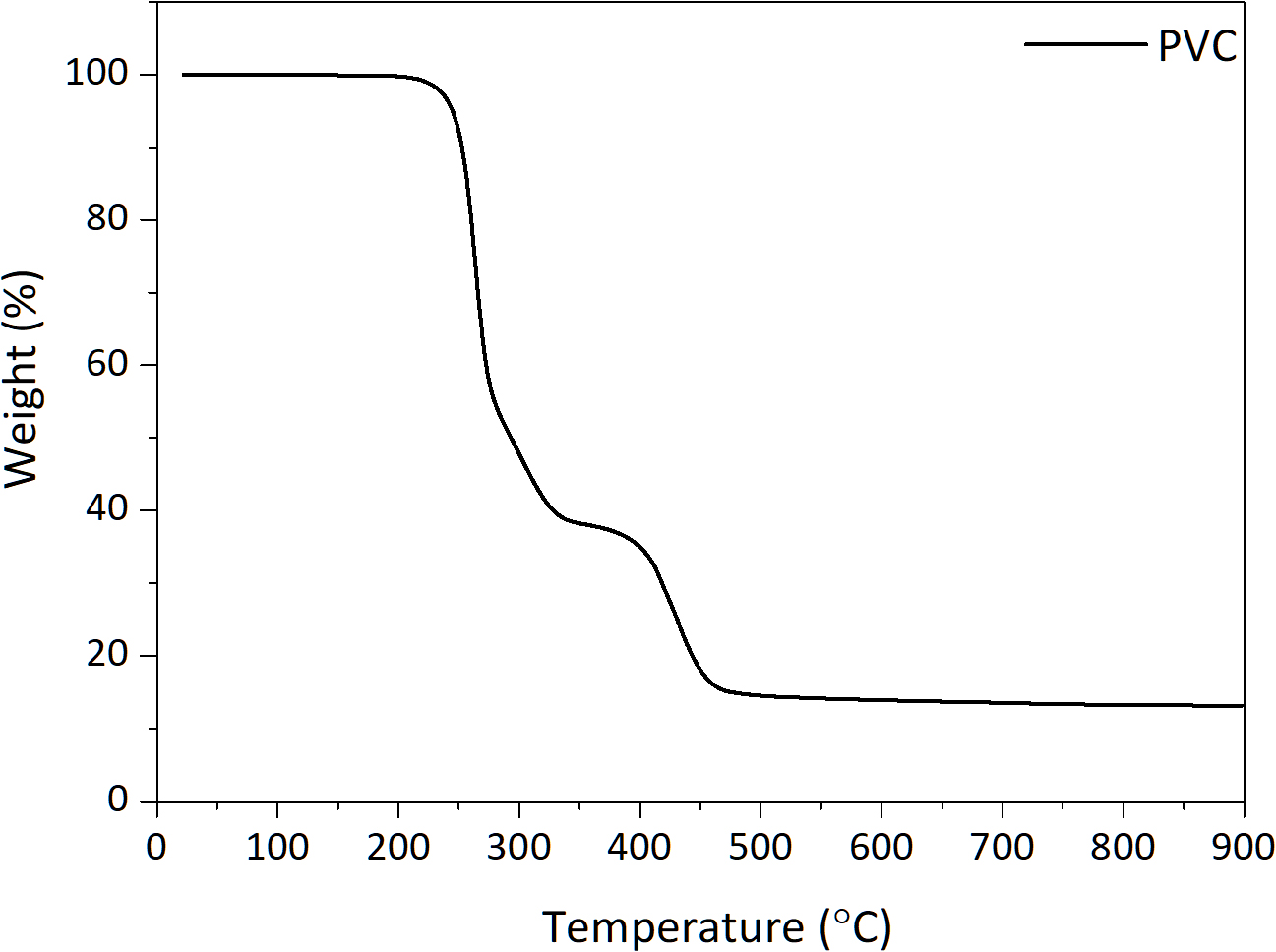


**Fig. S17** TGA curve of PVC under an N_2_ atmosphere


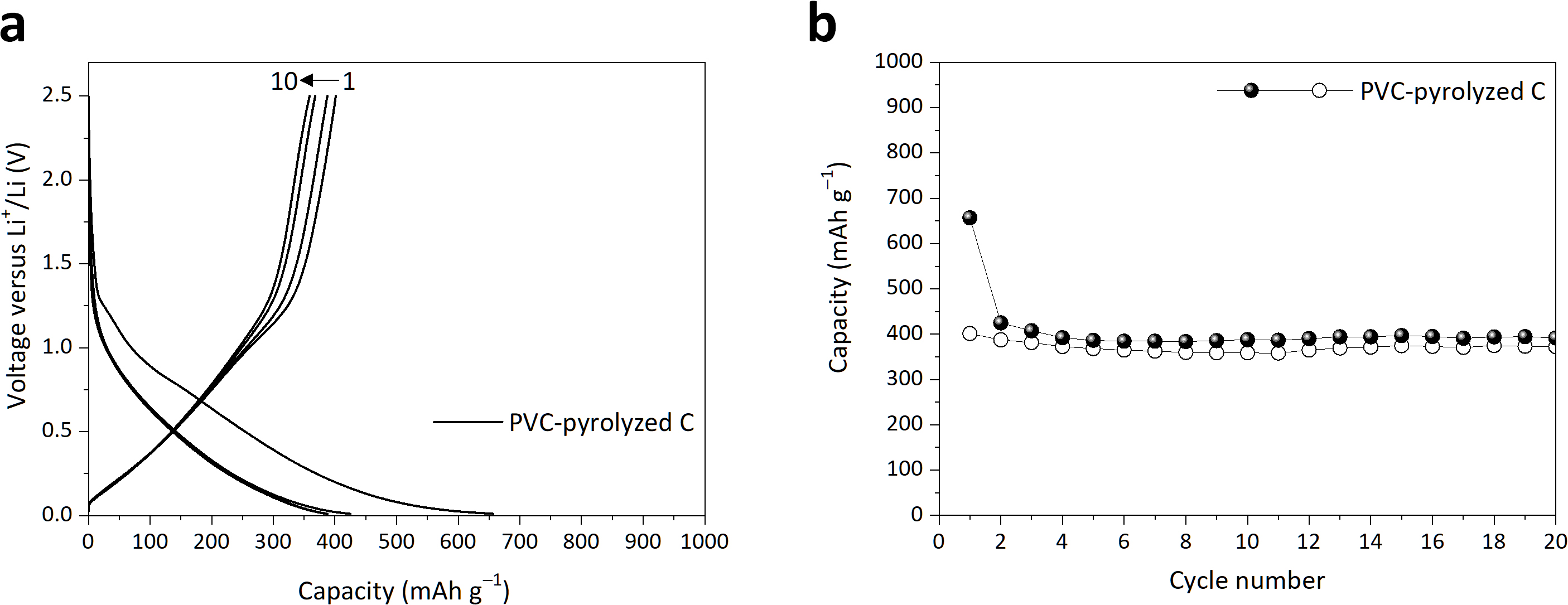


**Fig. S18** Electrochemical performance results for the PVC-pyrolyzed carbon anode: **a** voltage profiles and **b** cycling performance of the PVC-pyrolyzed carbon anode tested at 100 mA g^–1^


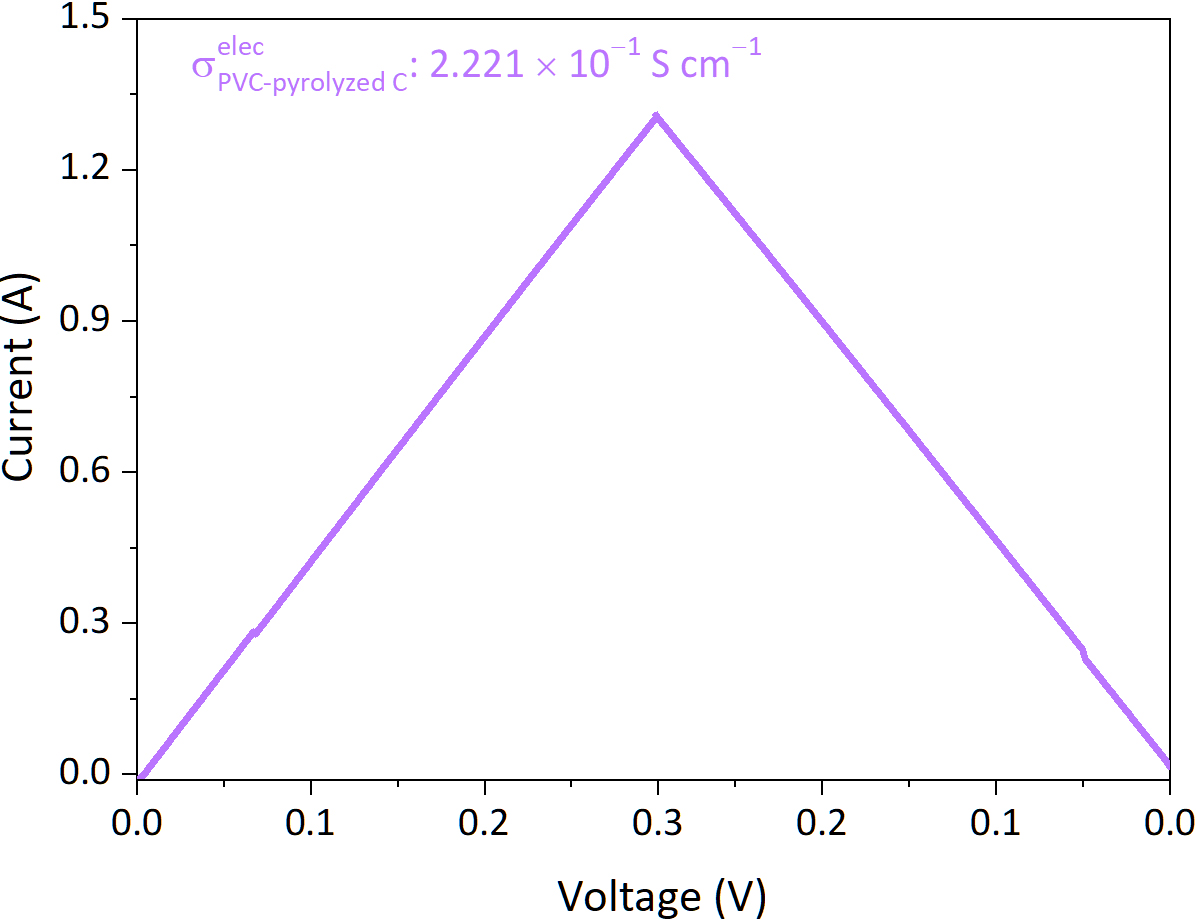


**Fig. S19** I–V profile of the PVC-pyrolyzed C and the corresponding calculated electronic conductivity


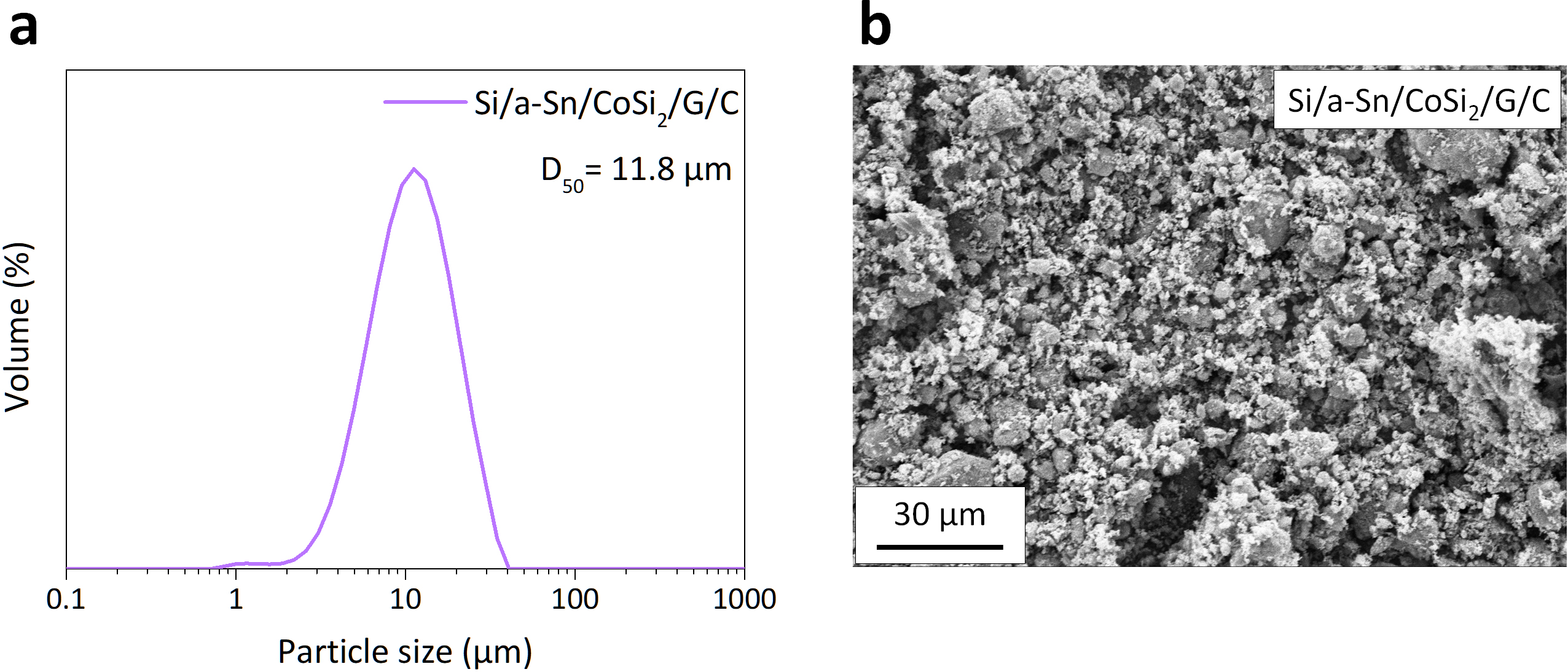


**Fig. S20** Morphological characteristics of the Si/a-Sn/CoSi_2_/G/C nanocomposite: **a** PSA result and **b** SEM image


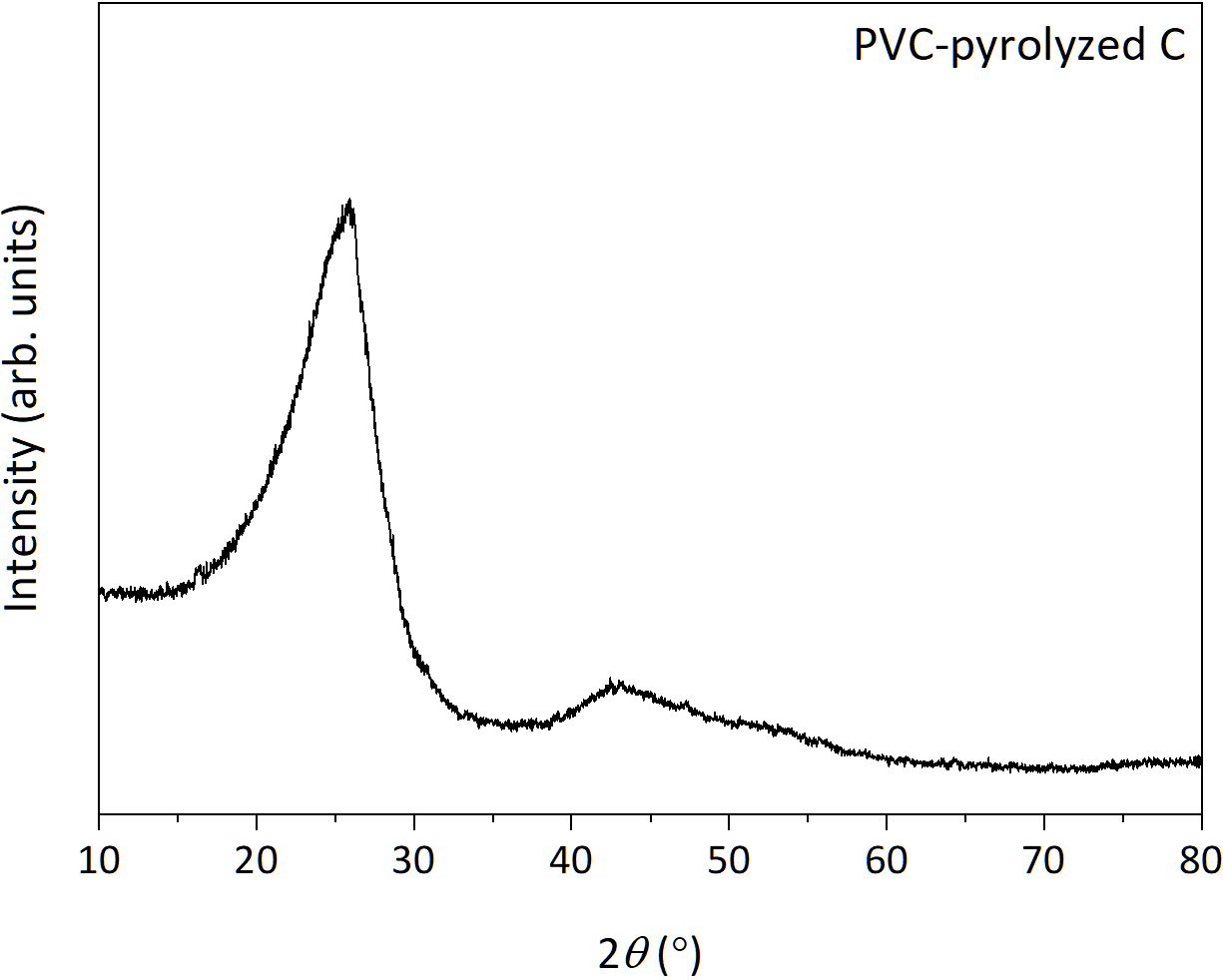


**Fig. S21** XRD pattern of the PVC-pyrolyzed carbon obtained by thermal pyrolysis at 700 °C under an Ar atmosphere


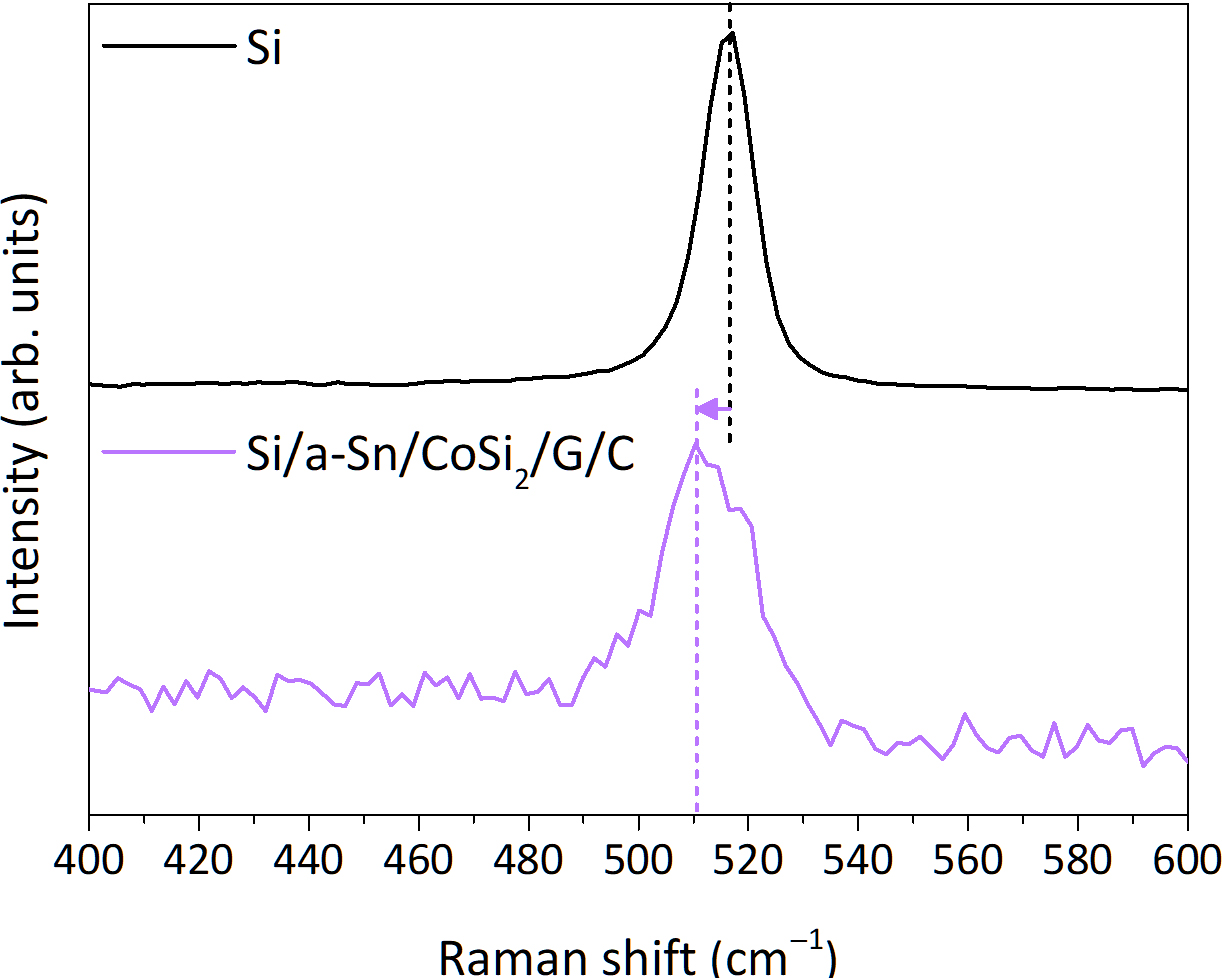


**Fig. S22** Raman spectra of the pristine Si and Si/a-Sn/CoSi_2_/G/C nanocomposite in the range of 400–600 cm^–1^


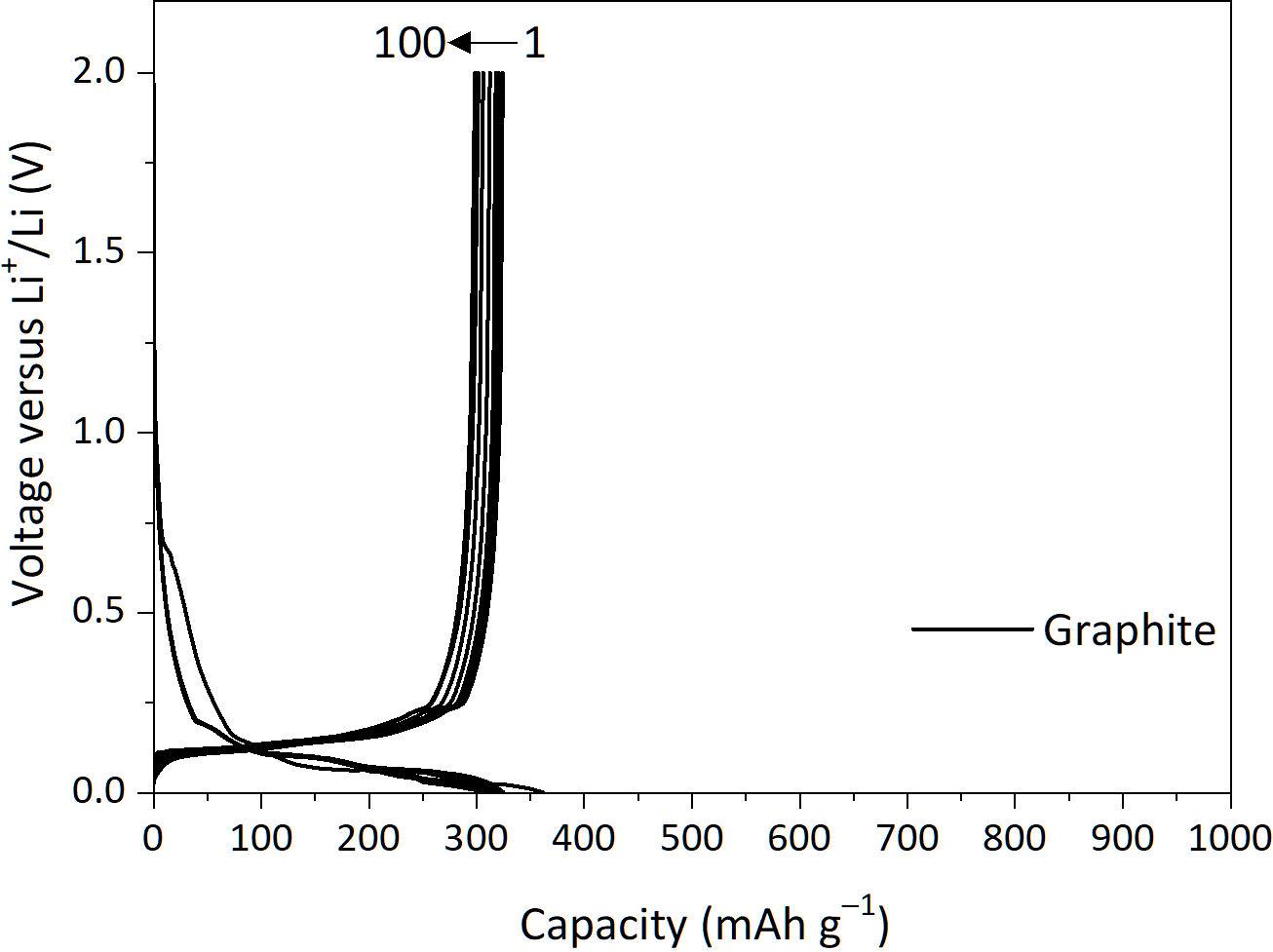


**Fig. S23** Voltage profiles of the commercial graphite anode tested at 100 mA g^–1^


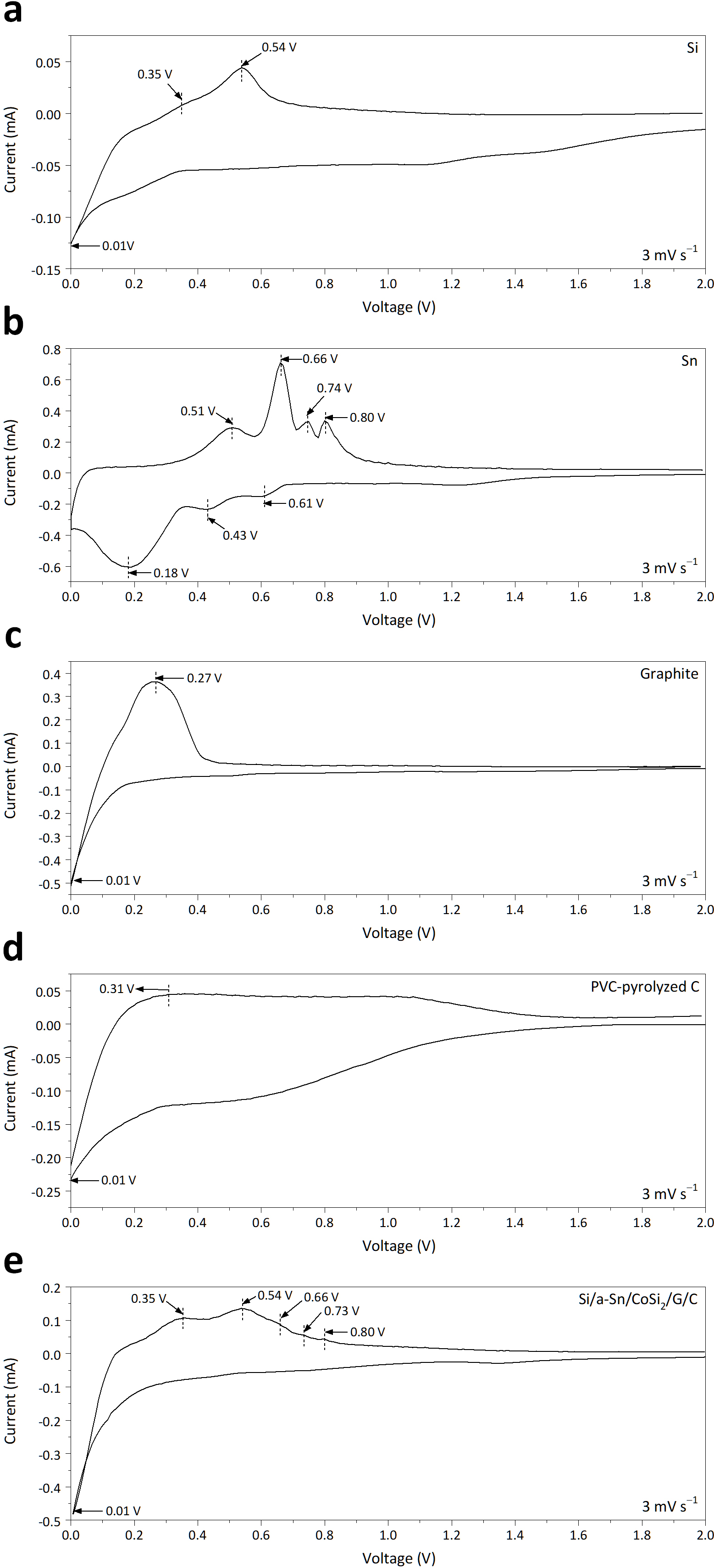


**Fig. S24** CV profiles of Si, Sn, graphite, PVC-pyrolyzed amorphous carbon, and the Si/a-Sn/CoSi_2_/G/C nanocomposite anode measured at a scan rate of 3 mV s^–1^ over a voltage range of 0.01–2.0 V (*vs.* Li^+^/Li)


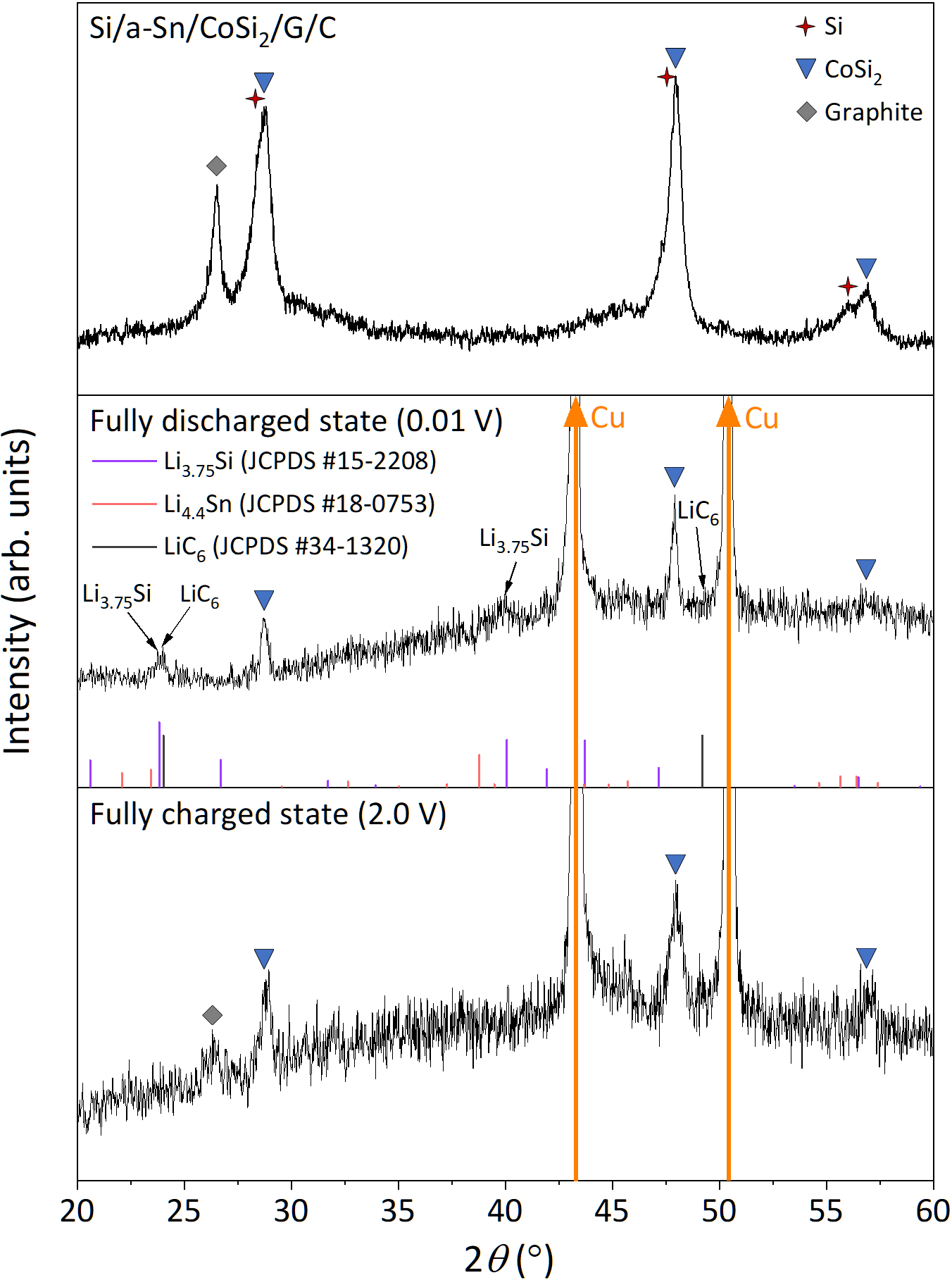


**Fig. S25** *Ex situ* XRD patterns of the Si/a-Sn/CoSi_2_/G/C anode at the pristine state, fully lithiated state (0.01 V), and delithiated state (2.0 V)


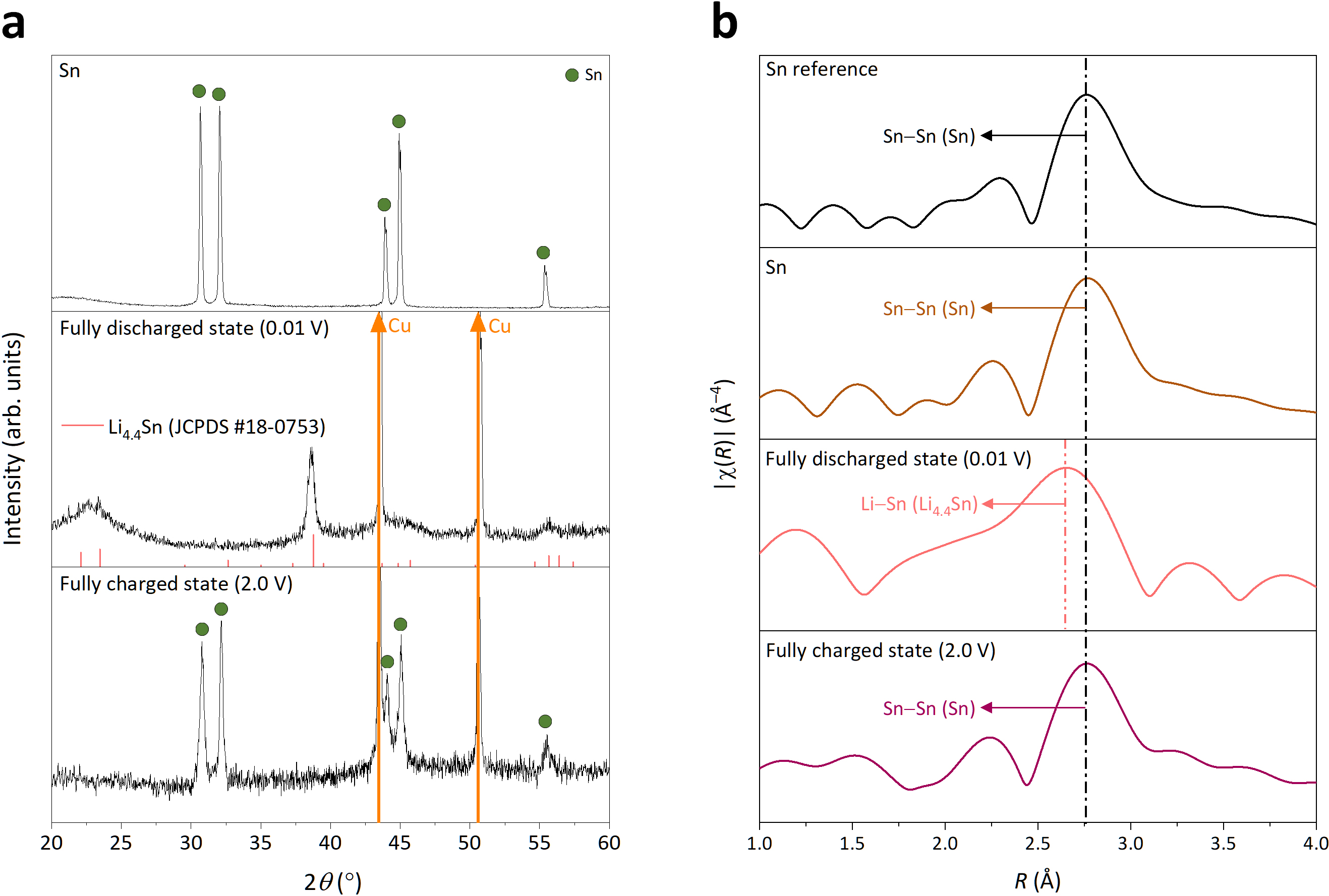


**Fig. S26** Phase evolution of the Sn reference anode at the pristine state, fully lithiated state (0.01 V), and delithiated state (2.0 V). **a** *Ex situ* XRD patterns and **b** *ex situ* Sn K-edge EXAFS spectra


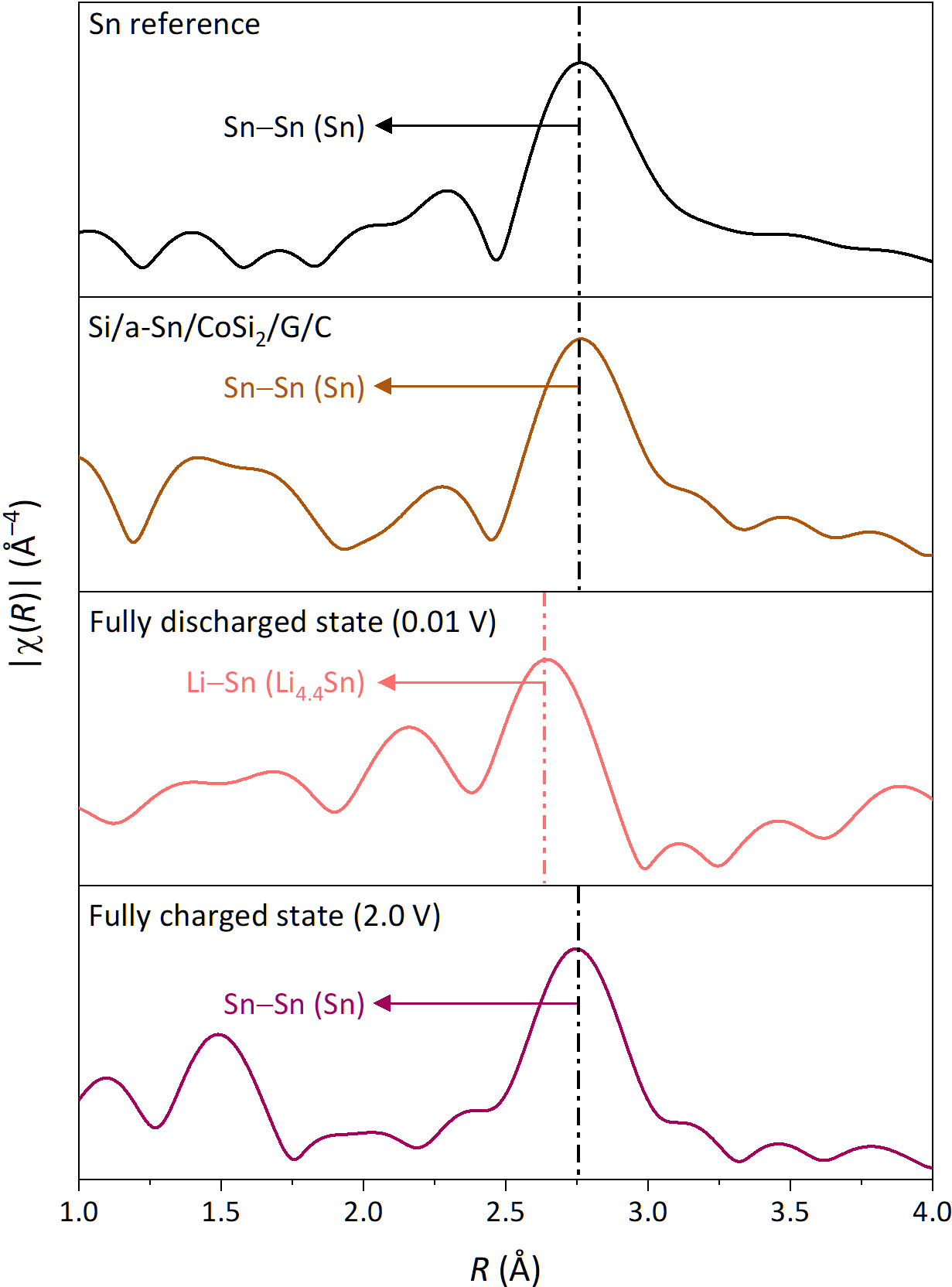


**Fig. S27** *Ex situ* Sn K-edge EXAFS spectra of the Si/a-Sn/CoSi_2_/G/C anode at the pristine state, fully lithiated state (0.01 V), and delithiated state (2.0 V)


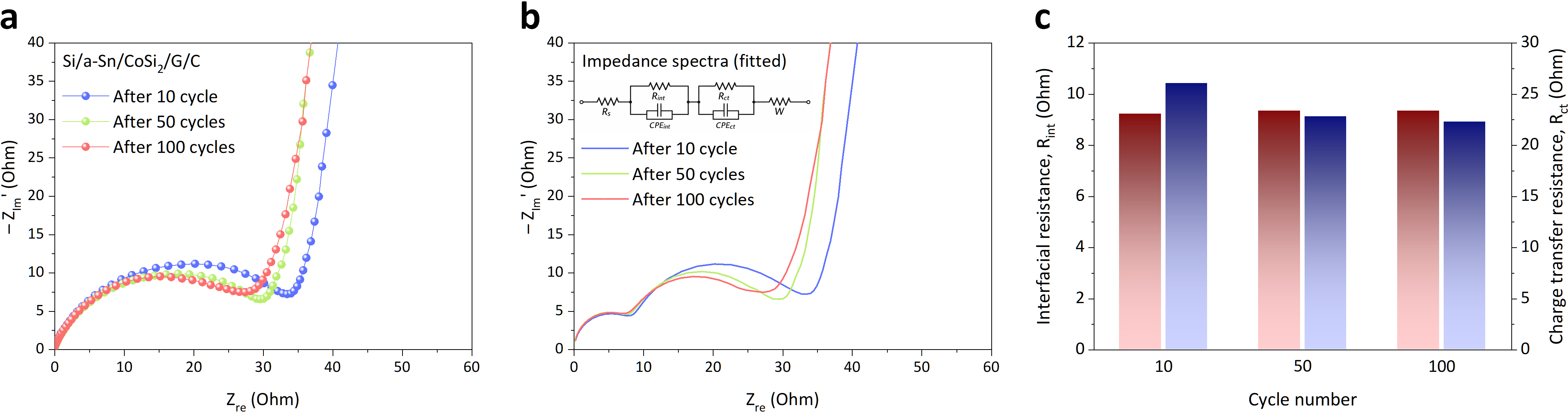


**Fig. S28** *Ex situ* EIS analysis of the Si/a-Sn/CoSi_2_/G/C anode after 10, 50, and 100 cycles. **a** Experimental Nyquist plots. **b** Equivalent circuit fitting of the impedance spectra using an R_s_–(R_int_||CPE_int_)–(R_ct_||CPE_ct_)–W model. **c** Evolution of R_int_ and R_ct_ extracted from the fitting


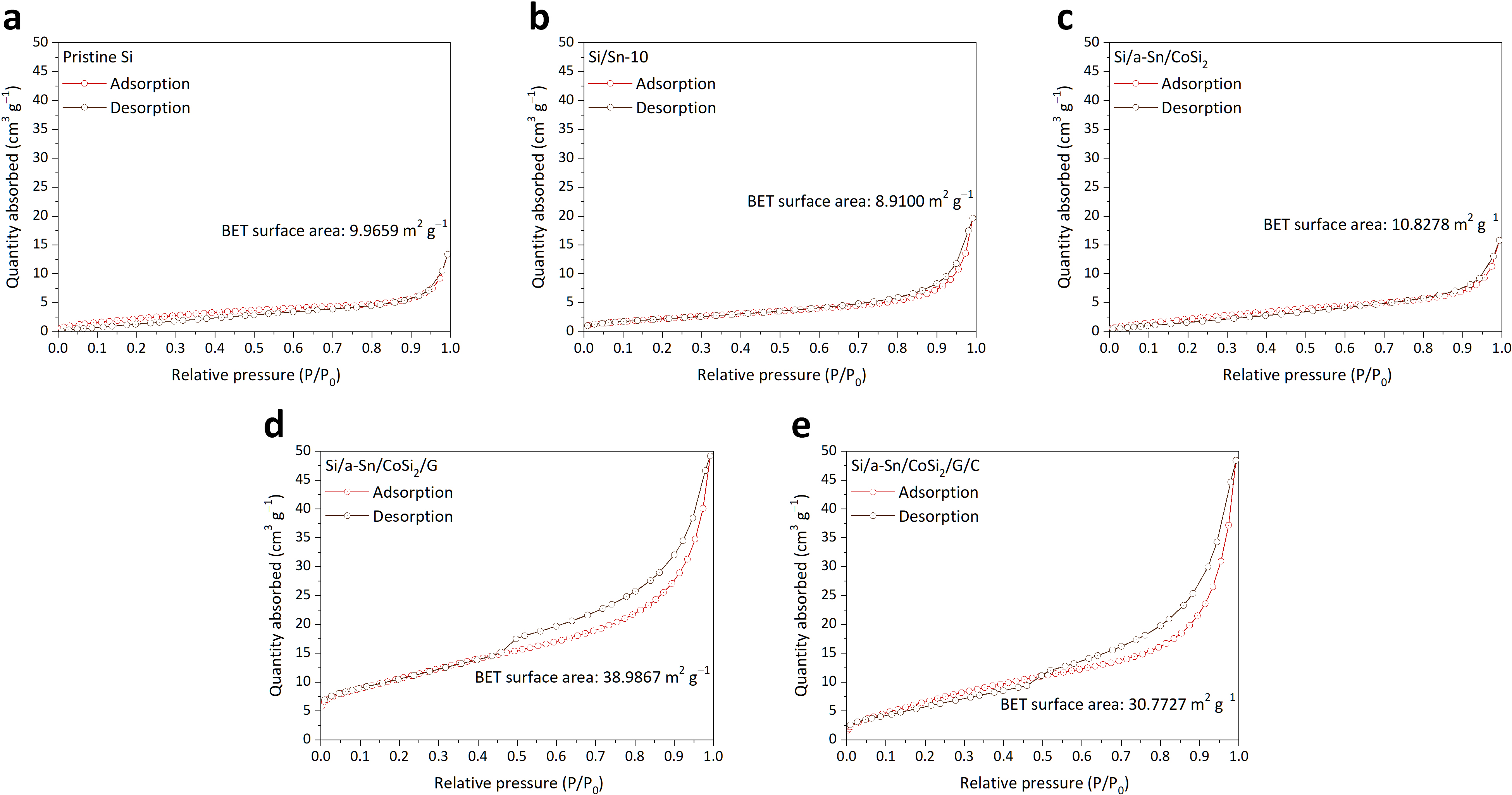


**Fig. S29** N_2_ adsorption–desorption isotherms of **a** pristine Si, **b** Si/Sn-10, **c** Si/a-Sn/CoSi_2_, **d** Si/a-Sn/CoSi_2_/G, and **e** Si/a-Sn/CoSi_2_/G/C composites


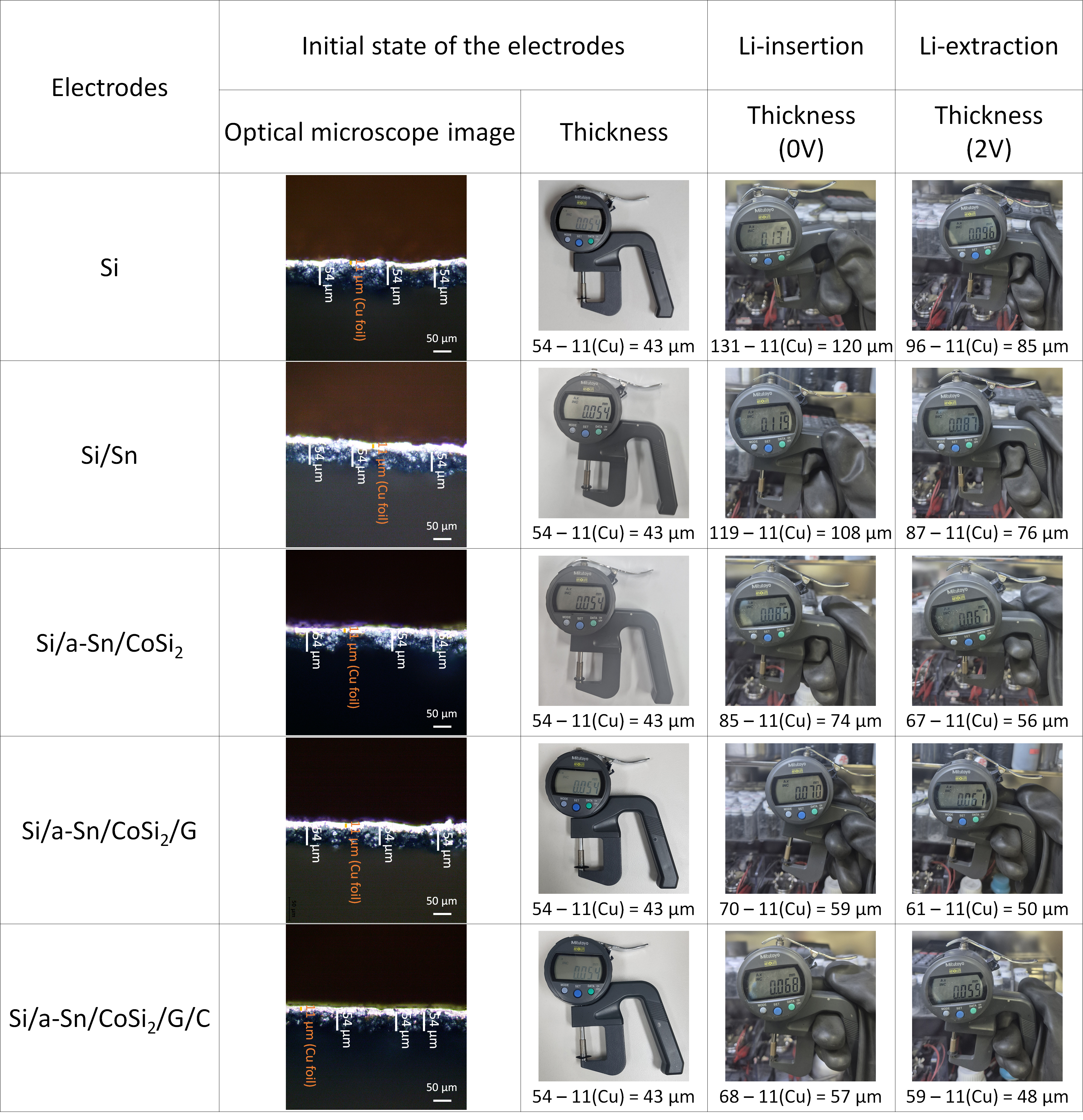


**Fig. S30** OM images and photographs showing changes in the thicknesses of pristine Si, Si/Sn, Si/a-Sn/CoSi_2_, Si/a-Sn/CoSi_2_/G, and Si/a-Sn/CoSi_2_/G/C anodes in the fully discharged (0 V *vs.* Li^+^/Li) and fully charged (2 V *vs.* Li^+^/Li) states


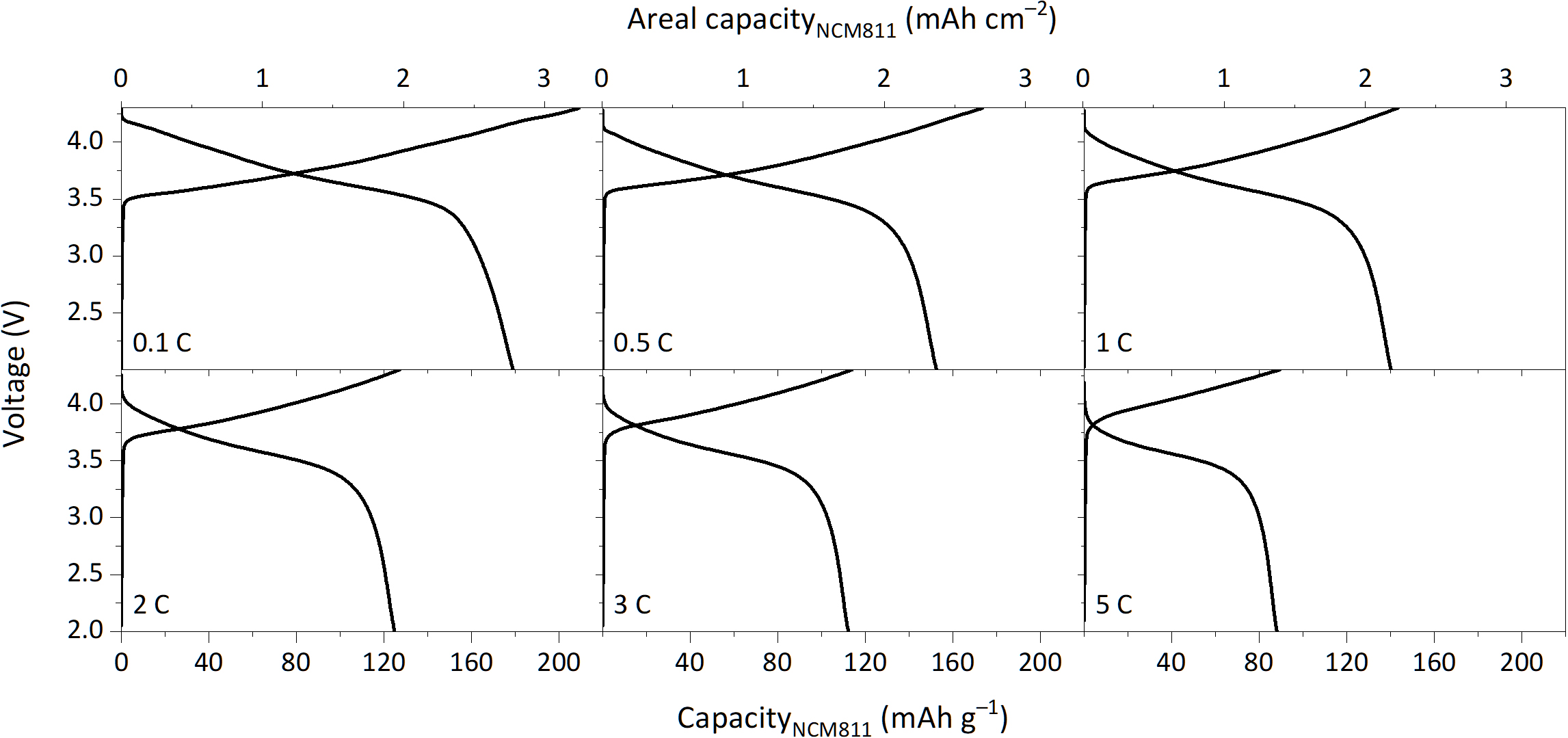


**Fig. S31** Voltage profiles of the graphite|NCM811 full cell measured at various current densities from 0.1 to 5 C with a cathode loading of 15.5 mg cm^–2^


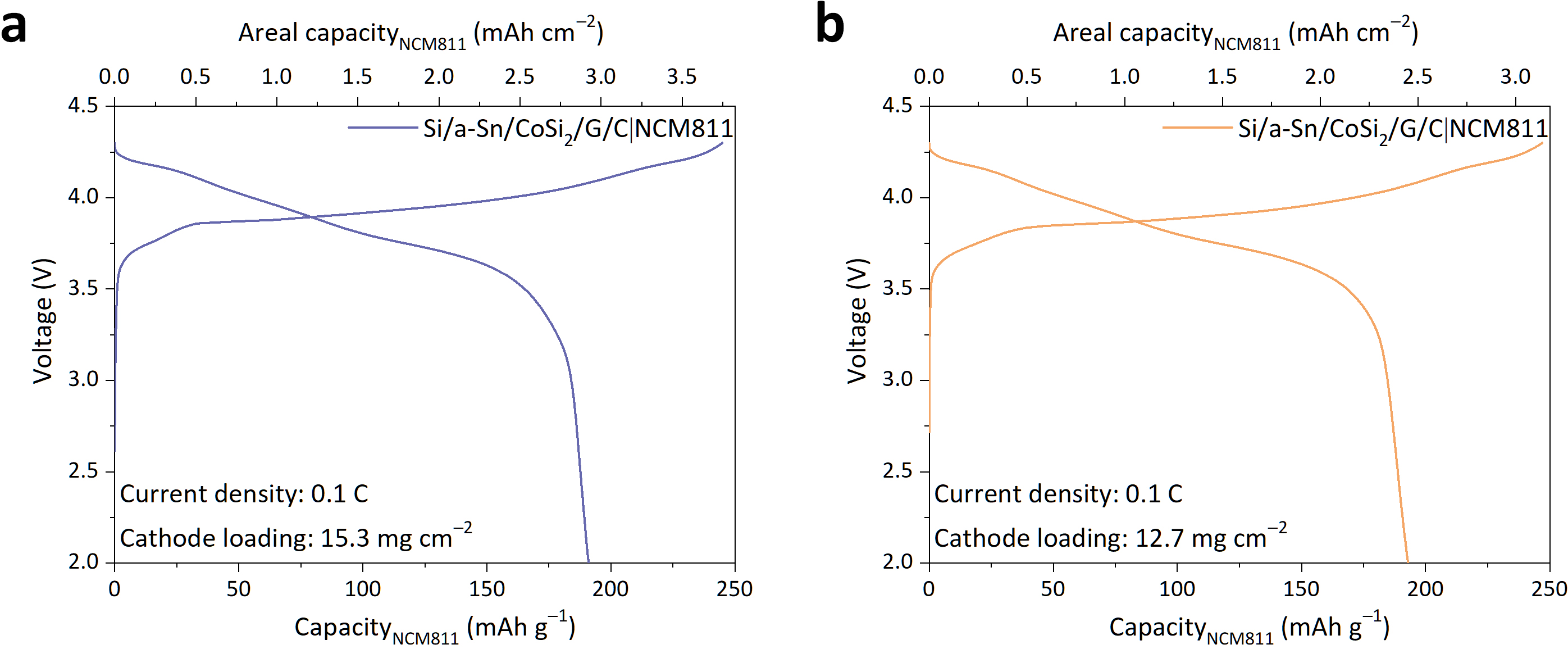


**Fig. S32** Voltage profiles of the Si/a-Sn/CoSi_2_/G/C|NCM811 full cells during the initial activation cycle at 0.1 C with cathode loadings of **a** 15.3 mg cm^–2^ and **b** 12.7 mg cm^–2^


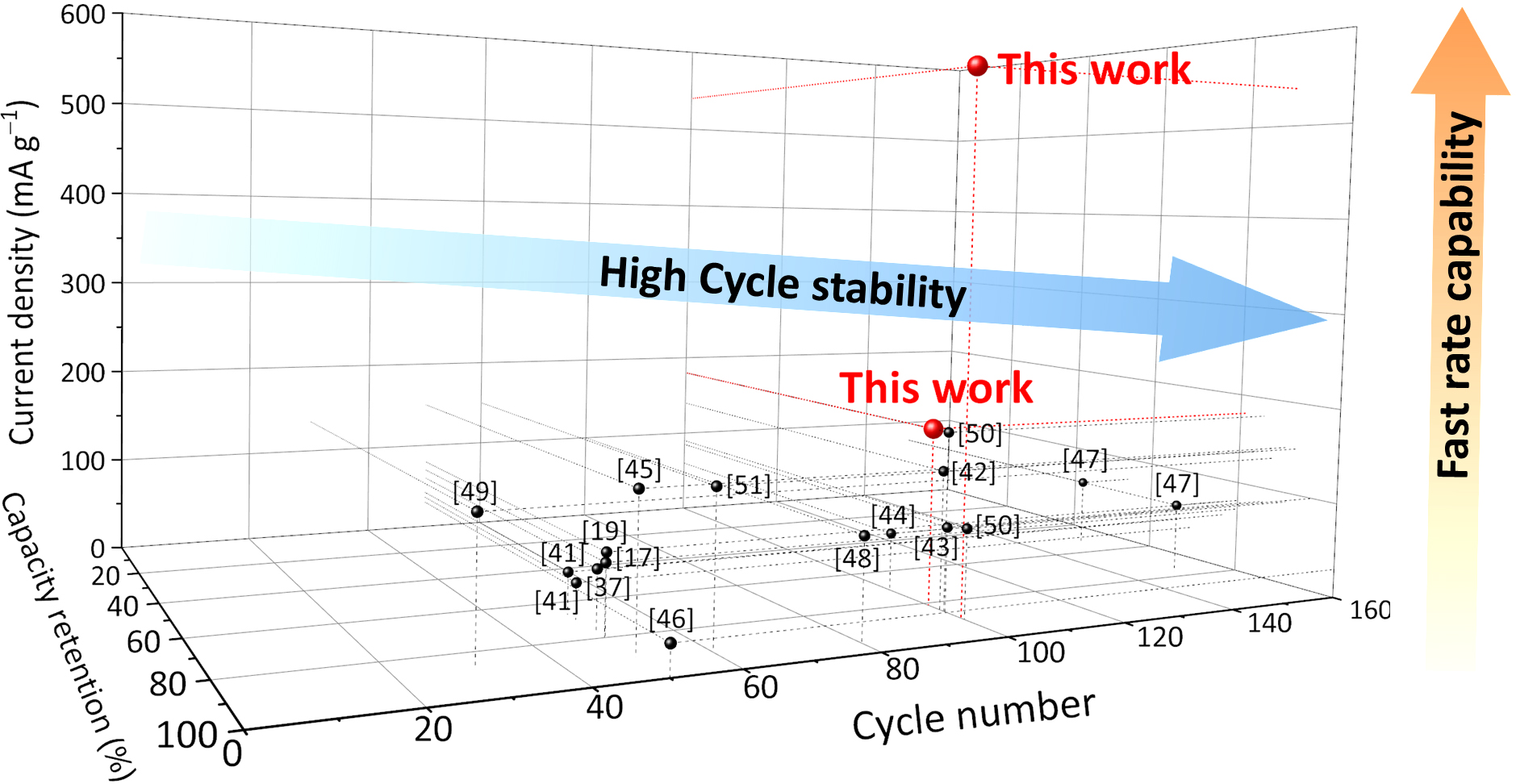


**Fig. S33** Comparison of the electrochemical performance of the Si/a-Sn/CoSi_2_/G/C|NCM811 full cell with those of previously reported Si-based full cells


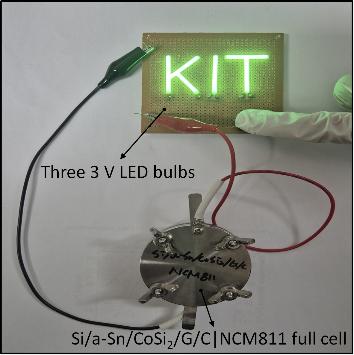


**Fig. S34** Photograph of three commercial green LED bulbs (3 V) powered by the Si/a-Sn/CoSi_2_/G/C|NCM811 full cell


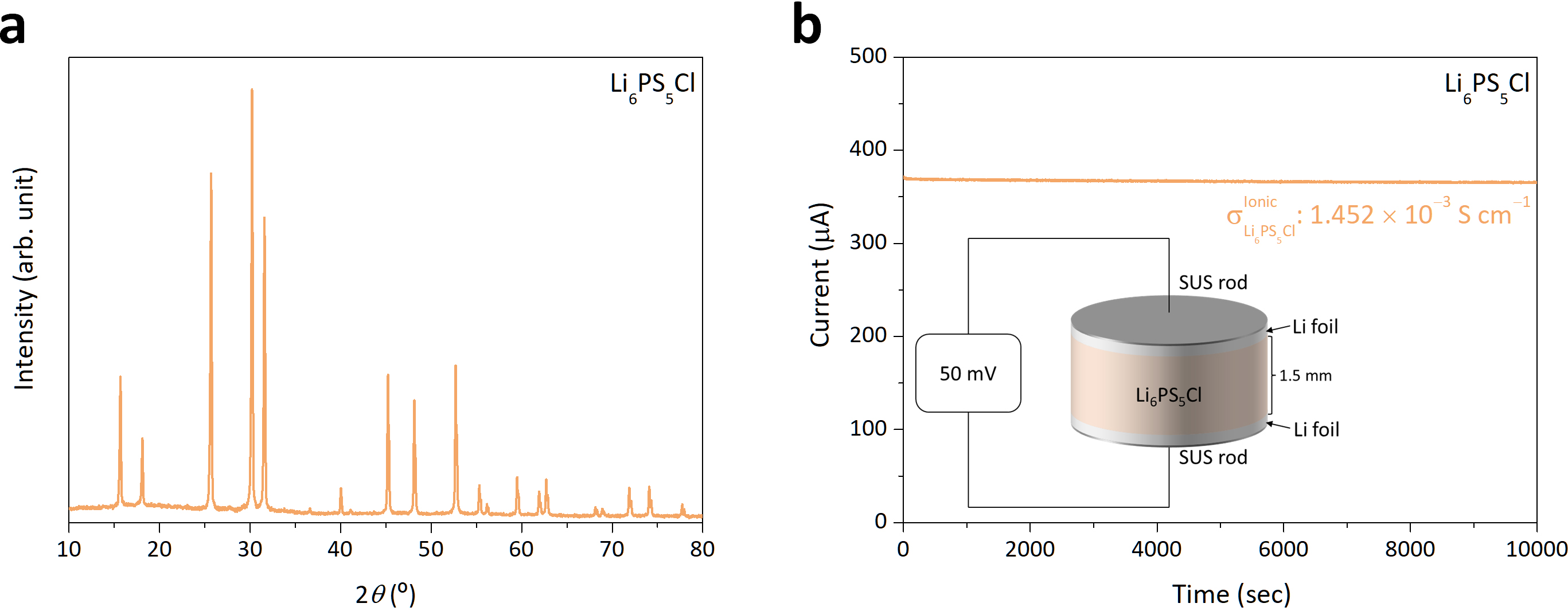


**Fig. S35** Structural characteristics and ionic conductivity of argyrodite LPSC SE: **a** XRD pattern and **b** ionic conductivity measured by DC polarization in an electron-blocking symmetric cell (DC bias: 50 mV)


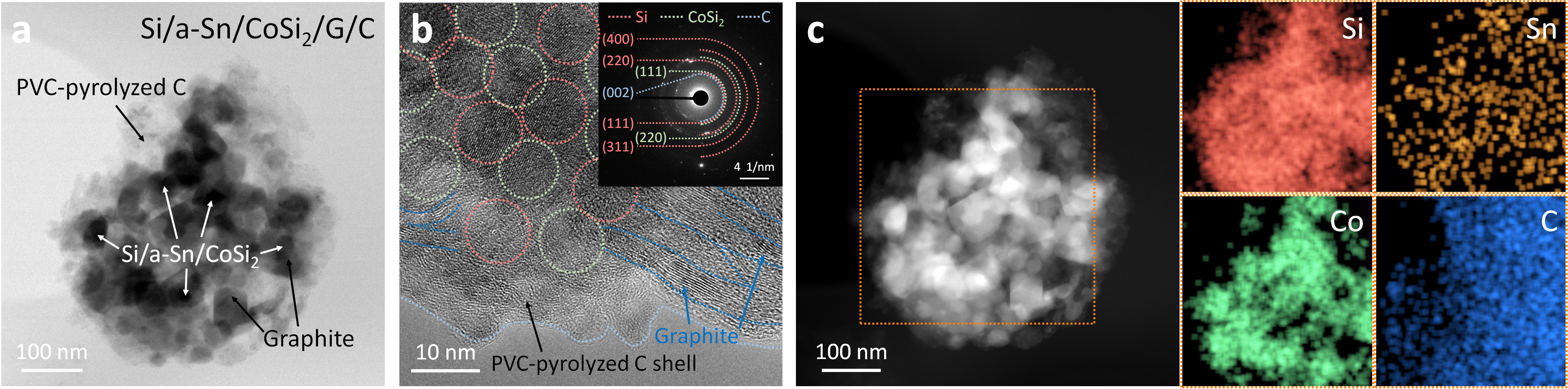


**Fig. S36** Structural characterization of the Si/a-Sn/CoSi_2_/G/C nanocomposite after pressing under 375 MPa for ASSLB assembly. **a** BFTEM image, **b** HRTEM image with the corresponding SAED pattern (inset), and **c** STEM image with the corresponding EDX elemental mappings of Si/a-Sn/CoSi_2_/G/C


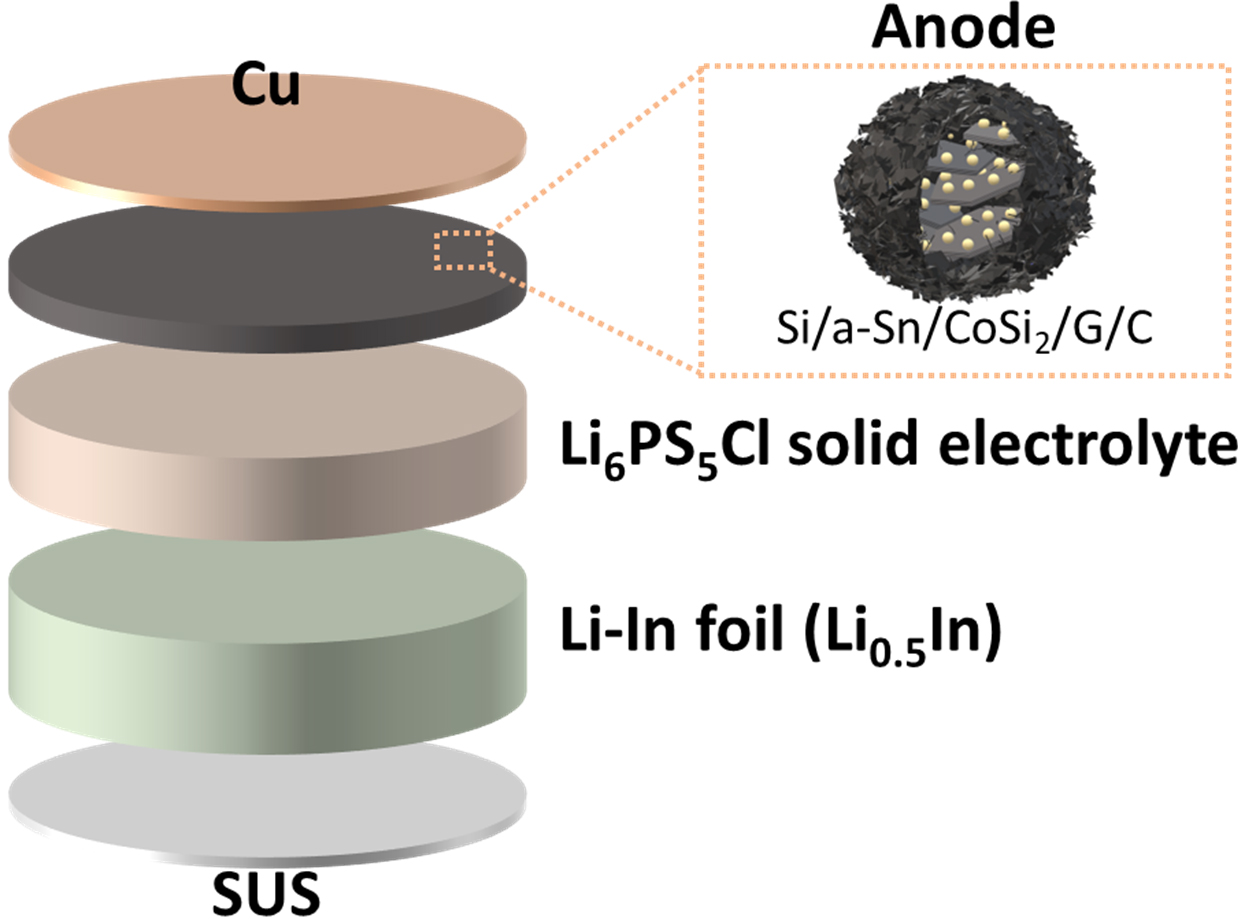


**Fig. S37** Schematic illustration of the ASSLB half-cell configuration


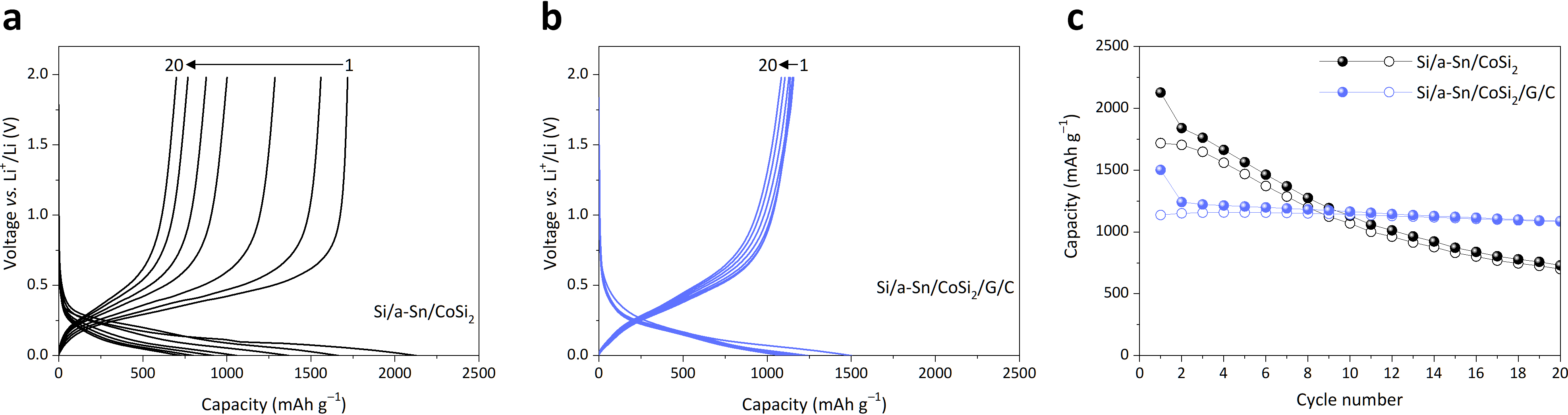


**Fig. S38** Electrochemical comparison of Si/a-Sn/CoSi_2_ and Si/a-Sn/CoSi_2_/G/C anodes in sulfide-based ASSLB half cells. Voltage profiles of **a** Si/a-Sn/CoSi_2_ and **b** Si/a-Sn/CoSi_2_/G/C anodes. **c** Cycling performance of Si/a-Sn/CoSi_2_ and Si/a-Sn/CoSi_2_/G/C anodes at a current density of 300 mA g^–1^


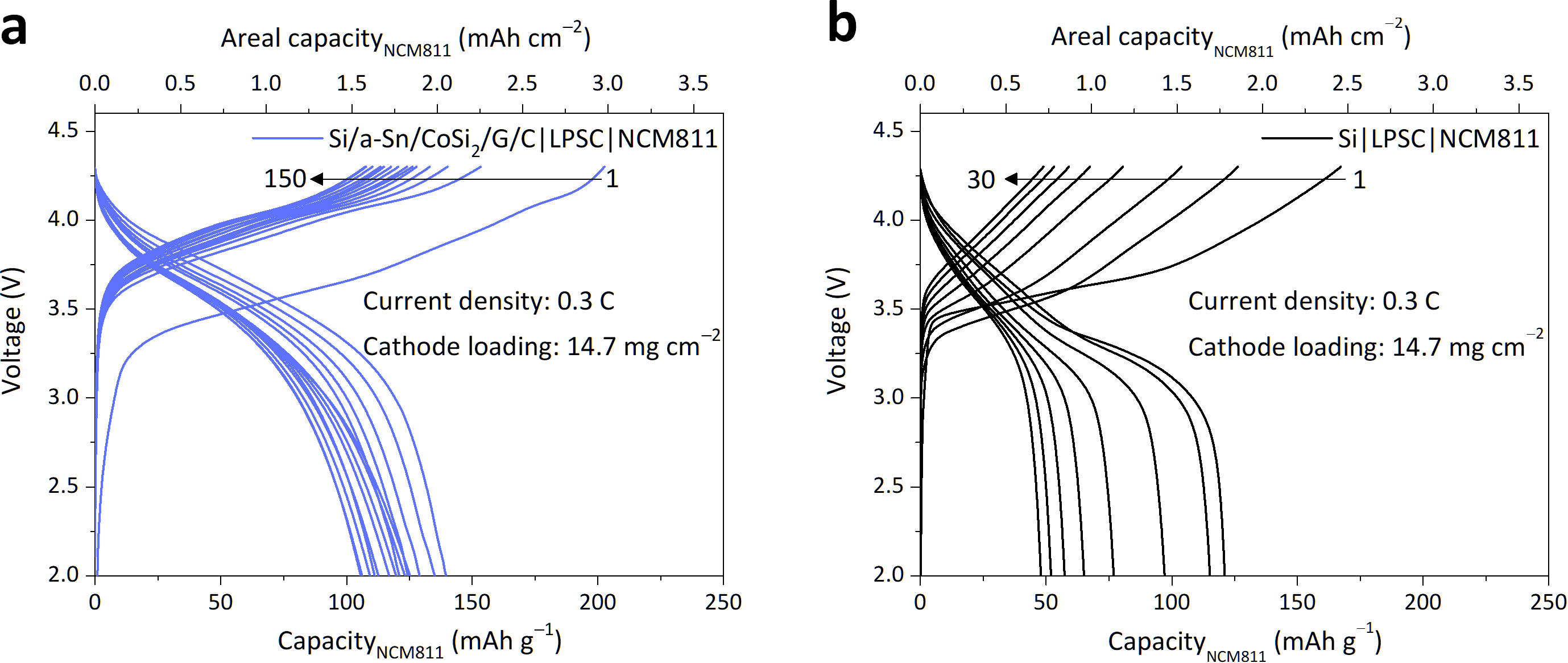


**Fig. S39** Electrochemical performances of the Si|LPSC|NCM811 and Si/a-Sn/CoSi_2_/G/C|LPSC|NCM811 full cells: **a** voltage profiles of the Si/a-Sn/CoSi_2_/G/C|LPSC|NCM811 and **b** Si|LPSC|NCM811 full cells at a current density of 0.3 C (cathode loading: 14.7 mg cm^–2^, temperature: 60 °C)


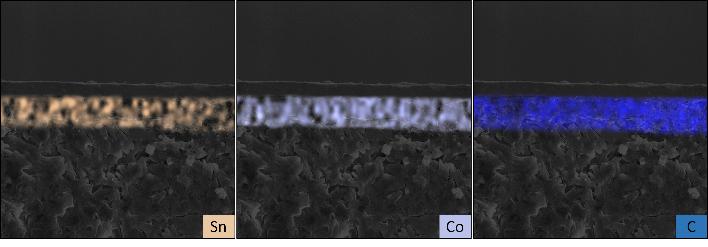


**Fig. S40** EDX elemental mapping of the Si/a-Sn/CoSi_2_/G/C anode before cycling in an ASSLB


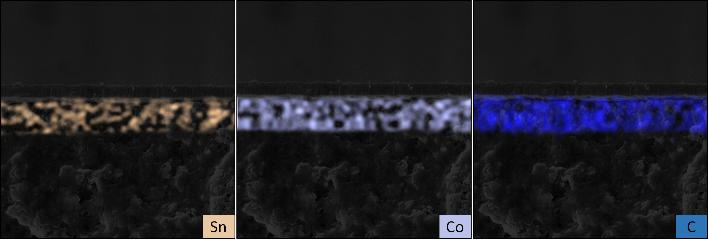


**Fig. S41** EDX elemental mapping of the Si/a-Sn/CoSi_2_/G/C anode after 100 cycles in an ASSLB

**Table S1** Electrochemical performance of pristine Si and Si/Sn composite anodes with varying Sn contents

| **Materials** | **Initial discharge capacity**  **(mAh g^–1^)** | **Initial charge capacity**  **(mAh g^–1^)** | **Initial Coulombic efficiency**  **(%)** | **Capacity retention after 30 cycles**  **(%)** |
| --- | --- | --- | --- | --- |
| Pristine Si | 3916.2 | 3275.4 | 83.6 | 3.9 |
| Si/Sn-5 | 3582.4 | 3167.9 | 88.4 | 25.0 |
| Si/Sn-10 | 3384.3 | 3129.2 | 92.5 | 33.2 |
| Si/Sn-15 | 3332.6 | 3049.8 | 91.5 | 33.8 |
| Si/Sn-20 | 3262.1 | 2955.7 | 90.6 | 34.0 |

**Table S2** Electrochemical performance of Si/a-Sn/TMS composite anodes

| **Materials** | **Initial discharge capacity**  **(mAh g^–1^)** | **Initial charge capacity**  **(mAh g^–1^)** | **Initial Coulombic efficiency**  **(%)** | **Capacity retention after 30 cycles**  **(%)** |
| --- | --- | --- | --- | --- |
| Si/a-Sn/FeSi_2_ | 2024.0 | 1763.1 | 87.1 | 74.6 |
| Si/a-Sn/CoSi_2_ | 1961.7 | 1771.5 | 90.3 | 83.2 |
| Si/a-Sn/NiSi_2_ | 2051.6 | 1756.9 | 85.6 | 74.9 |

**Table S3** Plastic and elastic deformation energies of Si and high-power MM-derived TMS particles derived from the load-displacement profiles

| **Materials** | **Plastic deformation energy**  **(10^–10^ J)** | **Elastic deformation energy**  **(10^–10^ J)** | **Total deformation energy**  **(10^–10^ J)** |
| --- | --- | --- | --- |
| Si | 30.372 | - | 30.372 |
| FeSi_2_ | 118.099 | 41.378 | 159.477 |
| CoSi_2_ | 121.098 | 56.869 | 177.967 |
| NiSi_2_ | 97.710 | 41.246 | 138.956 |

**Table S4** Comparison of the electrochemical performance of the Si/a-Sn/CoSi_2_/G/C anode with previously reported Si/TMS-based anodes for LIBs

| **Materials** | **ICE**  **(%)** | **Current density**  **(mA g^-1^)** | **Reversible capacity**  **after X^th^ cycle**  **(mAh g^-1^)** | **Capacity retention**  **(%)** | **Electrolyte** | **Refs.** |
| --- | --- | --- | --- | --- | --- | --- |
| Si/TiSi_2_ heterostructure | 59.4 | 1300 | 937  (X=100) | 79.3 | 1.0 M LiPF_6_  (EC/DEC) | [18] |
| Si@TiSi_2_@NC | 83 | 200 | 1553.3  (X=100) | 71.5 | 1.0 M LiPF_6_  (EC/DEC+5wt%FEC) | [19] |
| Porous Si@TiSi_2_ | 74.5 | 400 | 1180  (X=50) | 66.3 | 1.5 M LiPF_6_  (EC/DEC+25vol%FEC) | [20] |
| Si/FeSi_2_/CrSi_2_ | 86 | 1000 | 841  (X=100) | 87.9 | 1.0 M LiPF_6_  (EC/DEC+25vol%FEC) | [21] |
| Si-FeSi_2_-G-C | 87.0 | 100 | 925  (X=80) | 88.5 | 1 M LiPF_6_  (EC/DEC+10wt%FEC) | [22] |
| Si/FeSi_2_@C NPs | 87.6 | 200 | 1375  (X=100) | 56.1 | 1.0 M LiPF_6_  (EC/DEC/EMC+FEC) | [23] |
| Si/Co-CoSi_2_/rGO | 77.9 | 100 | 921.8  (X=80) | 98.6 | 1.3 M LiPF_6_  (EC/DMC) | [24] |
| Si@CoSi_2_/Co-NPC@CNT | 76.1 | 200 | 1258  (X=100) | 74.6 | 1.0 M LiPF_6_  (EC/DEC+5wt%FEC) | [25] |
| Si-NiSi_2_-Al_2_O_3_@C | 74.3 | 200 | 680  (X=100) | 94.4 | 1 M LiPF_6_  (EC/DEC+30vol%FEC) | [26] |
| Si/NiSi_2_/C | 76.9 | 200 | 1827.7  (X=50) | 87.6 | 1 M LiPF_6_  (EC/DMC+2wt%FEC) | [27] |
| Si/NiSi_2_/C | 78 | 1000 | 1500  (X=100) | 79.8 | 1 M LiPF_6_  (EC/DMC+2wt%FEC) | [27] |
| Cu-Si alloy nanotube | 42 | 840 | 1670  (X=100) | 77.6 | 1.0 M LiPF_6_  (EC/DEC) | [35] |
| Cu-Si alloy nanotube | 37 | 1600 | 1380  (X=100) | 77.1 | 1.0 M LiPF_6_  (EC/DEC) | [35] |
| Cu-Si alloy nanotube | 33 | 3200 | 1210  (X=100) | 76.2 | 1.0 M LiPF_6_  (EC/DEC) | [35] |
| Cu_3_Si/Si | 88.9 | 200 | 1675.4  (X=100) | 66.2 | 1.0 M LiPF_6_  (EC/DMC/EMC+5vol%FEC) | [36] |
| Si/Cu_3_Si/Al_2_O_3_ | 76 | 200 | 750  (X=100) | 89.2 | 1 M LiPF_6_  (EC/DMC+5vol%FEC) | [37] |
| Si-Cu_3_Si-CNT/G-C | 82.8 | 200 | 1084  (X=100) | 87.6 | 1.0 M LiPF_6_  (EC/DEC+10wt%FEC) | [38] |
| Cu_9_Si/Si NWs | 52.4 | 200 | 655.5  (X=100) | 55.6 | 1.0 M LiPF_6_  (EC/DMC/EMC+5vol%FEC) | [39] |
| Si/TiFeSi_2_ | 80 | 200 | 930  (X=100) | 93 | 1.3 M LiPF_6_  (EC/DEC+2%FEC) | [40] |
| **Si/a-Sn/CoSi_2_/G/C** | **80.7** | **300** | **1147.4**  **(X=100)** | **91.4** | **1.3 M LiPF_6_**  **(EC/DEC+10 wt%FEC)** | **This work** |

**Table S5** Comparison of the energy densities of graphite|NCM811 and Si/a-Sn/CoSi_2_/G/C|NCM811 full cells at various current densities

| **Anode material** | **Mass of anode**  **(mg)** | **Mass of**  **AAM**  **(mg)** | **Mass of separator (mg)** | **Mass of cathode**  **(mg)** | **Mass of NCM811**  **(mg)** | **Current density**  **(mA g^–1^)** | **Specific capacity (mAh g^–1^)** | **Average voltage**  **(V)** | **Energy**  **(Wh)** | **Energy density**  **(Wh kg^–1^)** |
| --- | --- | --- | --- | --- | --- | --- | --- | --- | --- | --- |
| Graphite | 8.61 | 6.89 | 1.76 | 15.23 | 12.18 | 18 | 179.0 | 3.63 | 0.00791 | 309.1 |
|  |  |  |  |  |  | 90 | 152.5 | 3.56 | 0.00661 | 258.3 |
|  |  |  |  |  |  | 180 | 140.2 | 3.54 | 0.00605 | 236.1 |
|  |  |  |  |  |  | 360 | 124.8 | 3.52 | 0.00535 | 209.0 |
|  |  |  |  |  |  | 540 | 112.2 | 3.50 | 0.00478 | 186.8 |
|  |  |  |  |  |  | 900 | 88.1 | 3.46 | 0.00371 | 145.0 |
| **Si/a-Sn**  **/CoSi_2_/G/C** | **2.87** | **2.01** | **1.76** | **15.23** | **12.18** | **18** | **187.4** | **3.78** | **0.00863** | **434.4** |
|  |  |  |  |  |  | **90** | **175.5** | **3.62** | **0.00774** | **389.6** |
|  |  |  |  |  |  | **180** | **174.2** | **3.54** | **0.00751** | **378.2** |
|  |  |  |  |  |  | **360** | **163.6** | **3.45** | **0.00687** | **346.2** |
|  |  |  |  |  |  | **540** | **151.9** | **3.37** | **0.00623** | **313.9** |
|  |  |  |  |  |  | **900** | **139.7** | **3.31** | **0.00563** | **283.6** |

**Table S6** Comparison of the electrochemical performance of the Si/a-Sn/CoSi_2_/G/C|NCM811 full cell with previously reported Si-based full cells

| **Anode** | **Cathode** | **Discharge capacity after X^th^ cycles**  **(mAh g^–1^)** | **Current density**  **(mA g^–1^)** | **Capacity retention**  **(%)** | **Refs** |
| --- | --- | --- | --- | --- | --- |
| carbon-coated Si nanosheets | LCO | 111.3 (X=50) | 70 | 80 | [17] |
| Si@TiSi_2_@NC | NCM622 | 143.9 (X=50) | 80 | 80.2 | [19] |
| Si-Cu_3_Si-Al_2_O_3_ | LMO | 70 (X=50) | 60 | 77.2 | [37] |
| Amorphous Columnar Si | NCM622 | 125 (X=50) | 36 | 70 | [41] |
| Amorphous Columnar Si | NCM811 | 142 (X=50) | 42 | 67 | [41] |
| Si@N-ECGB | NCM811 | 140 (X=100) | 140 | 84 | [42] |
| Si submicroncube@C | LFP | 122 (X=100) | 85 | 85.3 | [43] |
| Si-graphite | NCM523 | 119.3 (X=100) | 60 | 71 | [44] |
| Si/SiO_2_ | LCO | 149 (X=50) | 150 | 90 | [45] |
| Ag/ZnO-Si@C-PCNF | NCM513 | 135 (X=50) | 30 | 100 | [46] |
| Fe_14_Si_86_/C | NCM111 | 79 (X=150) | 75 | 54.1 | [47] |
| Fe_25_Si_75_ | NCM111 | 109 (X=150) | 75 | 77 | [47] |
| Si p-NS@TNS | LFP | 138 (X=80) | 100 | 95.2 | [48] |
| Si@graphene cage | LCO | 88 (X=30) | 135 | 87 | [49] |
| Si@C_MR_ | NCM622 | 151.3 (X=100) | 90 | 90 | [50] |
| Si@C_MR_ | NCM622 | 110.2 (X=100) | 180 | 85 | [50] |
| Si@C@MoS_2_ | LCO | 136.1 (X=60) | 150 | 91.7 | [51] |
| **Si/a-Sn/CoSi_2_/G/C** | **NCM811** | **137.1 (X=100)** | **180** | **81.2** | **This work** |
| **Si/a-Sn/CoSi_2_/G/C** | **NCM811** | **130.9 (X=100)** | **540** | **89.2** | **This work** |

**Table S7** Energy density calculation of the Si/a-Sn/CoSi_2_/G/C|LPSC|NCM811 ASSLB full cell

| Mass of anode (mg) | 2.83 |
| --- | --- |
| Mass of Si/a-Sn/CoSi_2_/G/C (mg) | 1.98 |
| Mass of 30 µm-thick LPSC SE (mg) | 4 |
| Mass of cathode (mg) | 15.60 |
| Mass of NCM811 (mg) | 12.01 |
| Current density (mA g^–1^) | 18 |
| Specific capacity (mAh g^–1^) | 162.1 |
| Average voltage (V) | 3.47 |
| Energy (Wh) | 0.00676 |
| **Energy density (Wh kg^–1^)** | **301.2** |

**Table S8** Comparison of the electrochemical performance of the Si/a-Sn/CoSi_2_/G/C|LPSC|NCM811 ASSLB full cell with previously reported Si-based ASSLB full cells

| **Anode** | **Cathode** | **Solid electrolyte** | **Cathode loading**  **(mg cm^–2^)** | **Stack pressure**  **(MPa)** | **Operating temp.**  **(°C)** | **Current density**  **(mA cm^–2^)** | **Areal capacity**  **(mAh cm^–2^)** | **Refs.** |
| --- | --- | --- | --- | --- | --- | --- | --- | --- |
| μm-Si | NCM111 | Li_3_PS_4_ | 18.3 | 75 | 30 | 0.15 | 2.20 | [53] |
|  |  |  |  |  |  | 0.3 | 1.83 |  |
|  |  |  |  |  |  | 0.75 | 1.28 |  |
|  |  |  |  |  |  | 1.5 | 0.64 |  |
|  |  |  |  |  |  | 3.0 | 0.27 |  |
|  |  |  |  |  |  | 4.5 | 0.15 |  |
|  |  |  |  |  |  | 6.0 | 0.07 |  |
| Li_6_PS_5_Cl-infiltrated Si | LCO | Li_6_PS_5_Cl | 10.0 | 140 | 30 | 0.14 | 1.04 | [54] |
|  |  |  |  |  |  | 0.28 | 0.90 |  |
| Si-SE-CB | NCM811 | Li_6_PS_5_Cl | 7.93 | 50 | 25 | 0.08 | 1.49 | [55] |
|  |  |  |  |  |  | 0.16 | 1.41 |  |
|  |  |  |  |  |  | 0.32 | 1.28 |  |
|  |  |  |  |  |  | 0.79 | 1.13 |  |
|  |  |  |  |  |  | 1.58 | 1.03 |  |
| Si-SE-C | NCM811 | Li_5.4_PS_4.4_Cl_1.6_ | 14.88 | 150 | 25 | 0.15 | 1.99 | [56] |
|  |  |  |  |  |  | 0.30 | 1.74 |  |
|  |  |  |  |  |  | 0.60 | 1.46 |  |
|  |  |  |  |  |  | 1.49 | 1.01 |  |
|  |  |  |  |  |  | 2.98 | 0.67 |  |
| Si | NCM811 | Li_3_PS_4_ | 13.6 | 75 | 30 | 0.15 | 1.77 | [57] |
|  |  |  |  |  |  | 0.30 | 1.49 |  |
|  |  |  |  |  |  | 0.75 | 1.16 |  |
|  |  |  |  |  |  | 1.5 | 0.84 |  |
|  |  |  |  |  |  | 3.0 | 0.48 |  |
|  |  |  |  |  |  | 4.5 | 0.27 |  |
|  |  |  |  |  |  | 6.0 | 0.14 |  |
| Col-Si | N_90_C_5_M_5_ | Li_6_PS_5_Cl | 12.9 | 3 N·m | 25 | 0.07 | 1.95 | [58] |
|  |  |  |  |  |  | 0.45 | 1.72 |  |
|  |  |  |  |  |  | 0.75 | 1.63 |  |
|  |  |  |  |  |  | 1.5 | 1.54 |  |
| Si@MOF | LFP | PVDF/PEO/  garnet | 15.4 | N/A | 60 | 0.1 | 2.20 | [59] |
|  |  |  |  |  |  | 0.2 | 1.40 |  |
|  |  |  |  |  |  | 0.5 | 0.80 |  |
|  |  |  | 20.2 | N/A | 60 | 0.1 | 2.60 |  |
|  |  |  |  |  |  | 0.2 | 1.80 |  |
|  |  |  |  |  |  | 0.5 | 1.10 |  |
| C-Li_1_Si-60 | NCM811 | Li_6_PS_5_Cl | 10.2 | 60 | 30 | 0.10 | 2.00 | [60] |
|  |  |  |  |  |  | 0.20 | 1.80 |  |
|  |  |  |  |  |  | 0.61 | 1.60 |  |
|  |  |  |  |  |  | 1.02 | 1.40 |  |
|  |  |  |  |  |  | 2.04 | 1.20 |  |
|  |  |  |  |  |  | 6.12 | 0.75 |  |
| In-Si | NCM90 | Li_5.5_PS_4.5_Cl_1.5_ | 8.75 | 50 | 55 | 1.23 | 1.08 | [61] |
|  |  |  |  |  |  | 4.92 | 0.94 |  |
|  |  |  |  |  |  | 7.38 | 0.86 |  |
| Li_21_Si_5_/  Si-Li_21_Si_5_ | LCO | Li_3_InCl_6_/  Li_6_PS_5_Cl | 23.4 | 0 | 45 | 0.6 | 2.32 | [62] |
|  |  |  |  |  |  | 1.3 | 1.85 |  |
|  |  |  |  |  |  | 2.5 | 1.46 |  |
|  |  |  |  |  |  | 3.8 | 1.23 |  |
|  |  |  |  |  |  | 5.0 | 1.01 |  |
|  |  |  |  |  |  | 6.3 | 0.79 |  |
|  |  |  |  |  |  | 7.6 | 0.57 |  |
| **Si/a-Sn**  **/CoSi_2_/G/C** | **NCM811** | **Li_6_PS_5_Cl** | **15.3** | **40** | **60** | **0.28** | **2.48** | **This work** |
|  |  |  |  |  |  | **0.55** | **2.32** |  |
|  |  |  |  |  |  | **1.38** | **2.02** |  |
|  |  |  |  |  |  | **1.93** | **1.82** |  |
|  |  |  |  |  |  | **2.75** | **1.63** |  |
|  |  |  |  |  |  | **5.50** | **1.38** |  |
|  |  |  |  |  |  | **8.25** | **1.13** |  |
